# Body Fat Free Mass Is Associated with the Serum Metabolite Profile in a Population-Based Study

**DOI:** 10.1371/journal.pone.0040009

**Published:** 2012-06-27

**Authors:** Carolin Jourdan, Ann-Kristin Petersen, Christian Gieger, Angela Döring, Thomas Illig, Rui Wang-Sattler, Christa Meisinger, Annette Peters, Jerzy Adamski, Cornelia Prehn, Karsten Suhre, Elisabeth Altmaier, Gabi Kastenmüller, Werner Römisch-Margl, Fabian J. Theis, Jan Krumsiek, H.-Erich Wichmann, Jakob Linseisen

**Affiliations:** 1 Institute of Epidemiology I, Helmholtz Zentrum München, German Research Center for Environmental Health, Neuherberg, Germany; 2 Institute of Genetic Epidemiology, Helmholtz Zentrum München, German Research Center for Environmental Health, Neuherberg, Germany; 3 Institute of Epidemiology II, Helmholtz Zentrum München, German Research Center for Environmental Health, Neuherberg, Germany; 4 Research Unit of Molecular Epidemiology, Helmholtz Zentrum München, German Research Center for Environmental Health, Neuherberg, Germany; 5 Institute of Experimental Genetics, Genome Analysis Center, Helmholtz Zentrum München, German Research Center for Environmental Health, Neuherberg, Germany; 6 Chair of Experimental Genetics, Technische Universität München, Munich, Germany; 7 Institute of Bioinformatics and Systems Biology, Helmholtz Zentrum München, German Research Center for Environmental Health, Neuherberg, Germany; 8 Department of Physiology and Biophysics, Weill Cornell Medical College, Education City, Qatar; 9 Institute of Medical Informatics, Biometry and Epidemiology, Chair of Epidemiology, Ludwig-Maximilians-Universität München, Neuherberg, Germany; 10 Klinikum Großhadern, Munich, Germany; University of Texas Health Science Center at San Antonio, United States of America

## Abstract

**Objective:**

To characterise the influence of the fat free mass on the metabolite profile in serum samples from participants of the population-based KORA (Cooperative Health Research in the Region of Augsburg) S4 study.

**Subjects and Methods:**

Analyses were based on metabolite profile from 965 participants of the S4 and 890 weight-stable subjects of its seven-year follow-up study (KORA F4). 190 different serum metabolites were quantified in a targeted approach including amino acids, acylcarnitines, phosphatidylcholines (PCs), sphingomyelins and hexose. Associations between metabolite concentrations and the fat free mass index (FFMI) were analysed using adjusted linear regression models. To draw conclusions on enzymatic reactions, intra-metabolite class ratios were explored. Pairwise relationships among metabolites were investigated and illustrated by means of Gaussian graphical models (GGMs).

**Results:**

We found 339 significant associations between FFMI and various metabolites in KORA S4. Among the most prominent associations (p-values 4.75×10^−16^–8.95×10^−06^) with higher FFMI were increasing concentrations of the branched chained amino acids (BCAAs), ratios of BCAAs to glucogenic amino acids, and carnitine concentrations. For various PCs, a decrease in chain length or in saturation of the fatty acid moieties could be observed with increasing FFMI, as well as an overall shift from acyl-alkyl PCs to diacyl PCs. These findings were reproduced in KORA F4. The established GGMs supported the regression results and provided a comprehensive picture of the relationships between metabolites. In a sub-analysis, most of the discovered associations did not exist in obese subjects in contrast to non-obese subjects, possibly indicating derangements in skeletal muscle metabolism.

**Conclusion:**

A set of serum metabolites strongly associated with FFMI was identified and a network explaining the relationships among metabolites was established. These results offer a novel and more complete picture of the FFMI effects on serum metabolites in a data-driven network.

## Introduction

The skeletal muscle mass is a major determinant of energy requirement of the body. It is a predictor of basal metabolic rate and energy turn-over during physical activity. In addition, it has recently been identified as an endocrine organ; producing and releasing myokines which exhibit various biological effects on the muscle tissue itself and beyond [1]. Among the most important effects of muscle mass and activity with respect to chronic diseases are enhanced fat oxidation, improved insulin sensitivity, and a reduced body fat mass. The effects of myokines may also be one explanation for the favourable effects that physical activity exerts on human health, e.g. by modulating the immune response. The skeletal muscle mass accounts for one-third to one-half of total body protein, depending on gender, age, and health status, and represents the largest fraction of the fat free body mass [2]. As pure skeletal muscle mass is difficult to measure in epidemiological studies, data on fat free mass was used as a proxy instead. Similar to the body mass index (BMI, kg/m^2^), the fat free mass index (FFMI, kg/m^2^) allows for height-independent interpretations and comparisons between studies [3].

So far, with an enormous effort over 4000 serum and plasma metabolites belonging to more than 50 different chemical classes have been identified, validated and characterised in the Serum Metabolome Database [4]. As this number exceeds the scope of most studies, a targeted metabolomics approach was chosen for our studies. The concept of targeted metabolomics is the quantification of a defined set of metabolites in a body fluid, representing an image of the current metabolic state of the organism [5]. It has been shown previously that this method has the power to identify perturbations of the body’s metabolic homeostasis and allows for the identification of and access to biomarkers of metabolic pathways that are impacted for example by diseases [6]–[11].

In this study, we took a targeted quantitative metabolomics approach to identify fat free mass/muscle mass related changes on human metabolism. To this end, the associations between FFMI and up to 190 serum metabolite concentrations including amino acids, acylcarnitines, phosphatidylcholines (PCs), sphingomyelins as well as hexose and biogenic amines measured by means of two different kits, were investigated in participants of the population-based studies KORA S4 and its seven-year follow-up KORA F4. As the biogenic amines are not part of the KORA F4 metabolomics dataset, the KORA S4 results were not discussed. However, they are shown in the Table S2. Furthermore, we computed Gaussian graphical models (GGMs), which have previously been shown to detect directly related metabolites in metabolomics data [12]. The resulting partial correlations in combination with the results from the linear regression models then provide a comprehensive picture of FFMI effects in a data-driven metabolic network.

## Materials and Methods

### Ethics Statement

The study was approved by the ethics committee of the Bavarian Medical Association. Written informed consent was obtained from each participant in accordance with institutional requirements and the Declaration of Helsinki Principles.

### Study Design and Population

The present analyses are based on subsamples of the metabolically characterised participants of the KORA S4 study and its seven-year follow-up KORA F4 study. KORA (Cooperative Health Research in the Region of Augsburg) is a research platform performing population-based surveys and subsequent follow-ups in the region of Augsburg in Southern Germany and has been described extensively [13]. The metabolic profiles of 1614 subjects in KORA S4 (ageing 54–75 years at the time of examination) and 3061 subjects in KORA F4 (ageing 31–82 years) were determined. The bioelectrical impedance analysis measurements of the participants’ body composition (fat free mass and body fat mass) which are part of the present analyses were only conducted in KORA S4. In order to account for this, only weight-stable participants of the KORA F4 population were included in the present study sample. A person was defined as weight-stable if their weight gain or loss did not exceed more than 0.5% per year since their body weight was measured in KORA S4 [14]. Further, subjects with a known history of myocardial infarction, stroke, diabetes or cancer were excluded as well as subjects taking ACE inhibitors or anti-lipidemic drugs. This resulted in a sample size of n = 965 for KORA S4 and n = 890 for KORA F4 with an overlap of n = 725 subjects.

### Blood Sampling

In both studies, fasting serum samples for metabolic analysis were collected during study centre visits. For KORA S4, the blood drawing occurred after a period of overnight-fasting (minimum of 8 hours) using S-Monovette® serum tubes (SARSTEDT AG & Co., Nümbrecht, Germany). Tubes were inverted two to three times, spent five minutes on the universal shaker (SARSTEDT AG & Co., Nümbrecht, Germany) before being allowed to rest for 40 minutes at 4°C for total coagulation. Later on, tubes were centrifuged for 15 minutes at 2,660 g, serum was separated and filled into synthetic straws which were stored in liquid nitrogen (−196°C) until analysis. The collection and procedure for the KORA F4 samples has previously been described [15].

### Metabolite Quantification

Metabolic characterisation of the KORA F4 serum samples was done in 2009 in three batches of approximately 1000 samples at three different time points with a recalibration of the equipment in between; whereas the complete KORA S4 set was characterised in 2011 in one batch.

The targeted metabolomics approach for KORA F4 and S4 was based on ESI-(LC-) MS/MS measurements by the Absolute*IDQ*™ p150 kit and p180 kit (BIOCRATES, Life Sciences AG, Innsbruck, Austria), respectively. The assays allow simultaneous quantification of 163 (kit p150; F4) or 186 (kit p180; S4) metabolites out of 10 µl serum in each case. The Absolute*IDQ*™ p150 kit has previously been described in detail [15], [16]. The p180 kit is an extension of it, using additional LC-MS/MS separation. For both kits, sample handling was performed by a Hamilton Micro Lab Star robot (Hamilton Bonaduz AG, Bonaduz, Switzerland) and a nitrogen evaporator (Porvair, Ultravap). Mass spectrometry (MS) analyses were done on a 4000 QTRAP mass spectrometer (AB Sciex) coupled to Promincence HPLC (Shimadzu) apparatus (F4) and an API 4000 LC-MS/MS System (AB Sciex Deutschland GmbH, Darmstadt, Germany) equipped with an Agilent 1200 Series HPLC and a HTC PAL auto sampler (CTC Analytics, Zwingen, Switzerland) (S4) controlled by the software Analyst 1.4 for kit p150 (F4) 1.5.1 for kit p180 (S4). Data evaluation for quantification of metabolite concentrations and quality assessment was performed with the Met*IQ*™ software package, which is an integral part of the Absolute*IDQ*™ kits. Internal standards serve as reference for the calculation of metabolite concentrations. The methods of the Absolute*IDQ*™ p150 and p180 kits have been proven to be in conformance with the FDA Guideline [17], which implies proof of reproducibility within an given error range. Measurements were performed as described in the manufacturer manuals. Concentrations are reported in µmol/l.

### Metabolite Spectrum

In total, up to 190 different metabolites were quantified. Kit p150 (KORA F4; 163 metabolites) contains 14 amino acids (13 proteinogenic + ornithine), hexose (sum of hexoses – about 90–95% glucose), free carnitine and 40 acylcarnitines (C*x*:*y*), 15 sphingomyelins (SM*x*:*y*), 77 phosphatidylcholines (PCs, aa = diacyl, ae = acyl-alkyl) and 15 lyso-phosphatidylcholines (lysoPCs). The lipid side chain composition is abbreviated as C*x*:*y*, with *x* denoting the number of carbons in the side chain and *y* denoting the number of double-bonds. Kit p180 (KORA S4; 186 metabolites) includes 21 amino acids (19 proteinogenic + citrulline + ornithine), hexose, free carnitine, 39 acylcarnitines, 15 sphingomyelins, 90 phosphatidylcholines (14 lysoPC and 76 PCs) as well as 19 biogenic amines. The overlap of both kits is 159 metabolites. Full biochemical names and abbreviations are provided in Table S1.

### Quality Control of the Metabolomics Dataset

The quality control of the metabolomics dataset of KORA F4 was done in a two-step procedure. First, the quality of all metabolites was assessed using a reference blood which was measured five times on ten plates. With this data, a coefficient of variation was calculated for every metabolite and plate. All metabolites having a mean coefficient of variation over all ten plates bigger than 25% were removed from the dataset (eleven in total). One further metabolite was excluded as the number of missing values exceeded 5%. In the second step, the dataset was controlled for outliers. A subject’s metabolite concentration was defined as an outlier if the concentration was greater or less than the mean ± five standard deviations of the particular metabolite over the whole population. All subjects having more than three independent outlying metabolite concentrations were excluded from the dataset. An outlier was defined as independent if the correlation with all other outliers was less than 70%. If there were three or less independent outliers, only the data points were removed. All missing values were imputed with the R package “mice” which uses a linear regression approach. For the quality control of the KORA S4 metabolite dataset, a new coefficient of variation was calculated using the reference blood which was on all KORA S4 plates. The same quality criteria were applied to the KORA S4 sample, resulting in 20 metabolites being excluded from the dataset. This left us with 151 metabolites for the KORA F4 dataset and 166 metabolites for KORA S4, with an overlap of 141 metabolites. As the metabolic profile in KORA F4 samples was assessed in three batches a so called batch variable was included in all analyses of the F4 metabolomics dataset in order to avoid possible effects due to technical issues or different time points of analyses. This step was not necessary for the KORA S4 dataset.

### Anthropometric and Body Composition Assessment

Height and weight were measured to the nearest 0.1 cm and 0.1 kg, respectively. Waist circumference was measured to the closest 0.1 cm at the smallest position between the lower rip and the upper margin of the iliac crest. Hip size was determined exactly to 0.1 cm as the widest circumference measured between the upper margin of the iliac crest and the crotch. For the assessment of body composition, two bioelectrical impedance analysis measurements of resistance (R), reactance (Xc) and the phase angle (α) were taken between the dominant hand wrist and dorsum and the dominant foot angle and dorsum (placement of the electrodes) by means of a body impedance analyser (BIA 2000-S; Data Input GmbH, Frankfurt, Germany) while subjects were spreading their arms and legs and lying in a relaxed and supine position on a nonconductive surface with 50 kHz. Fat free mass, fat mass, and the appendicular skeletal muscle mass were then calculated by means of Kyle’s equations [18], [19] on which the following indices are based: FFMI (fat free mass in kg/(height in m)^2^), body fat mass index (body fat mass in kg/(height in m)^2^) and the appendicular skeletal muscle mass index (appendicular skeletal muscle mass/(height in m)^2^.

### Genotyping, Imputation and SNP Selection

In KORA F4, genotyping was done by means of Affymetrix Human SNP Array 6.0. HapMap CEU version 22 was used as population reference and as reference for the imputation of the genotyped SNPs with IMPUTE v0.4.2. The complete procedure has been described in more detail [15]. The present analysis includes 170 SNPs. The selection of these SNPs is based on an extensive literature research of genome-wide association studies with an anthropometric characteristic as outcome. A list of the selected SNPs can be found in Table S4.

### Statistical Analyses

The descriptive data is presented as mean and standard deviation for the continuous variables and as absolute quantities and percentages for the discrete parameters. Sports activity was defined by the amount of regular leisure time exercise per week; more than one hour equals active; less equals inactive. The KORA S4 and F4 samples were analysed as independent cross-sectional studies. Besides the absolute metabolite concentrations, all pairs of intra-metabolite class ratios (n = 4629 for KORA S4 and n = 4518 for KORA F4) were part of the metabolomics datasets. Associations between metabolite concentrations or metabolite ratios and the FFMI were assessed by means of linear regression models. First, metabolite variables (absolute concentrations and ratios) were standardised with an inverse log-normal transformation to allow for comparison of the estimates derived from the linear regression models. Then, linear regression models were applied with the metabolite variables as dependent variables and the FFMI as explanatory variable. The models for the KORA S4 metabolomics data were adjusted for sex and age, whereas the models for KORA F4 were adjusted for age, sex, and batch. To control for multiple testing and with regard to the dependencies among the various metabolites and ratios, a p-value of 3.12×10^−04^ for KORA S4 and a p-value of 3.5×10^−04^ for KORA F4 were considered statistically significant at α = 5%. For an association with a metabolite ratio to be regarded as statistically significant, an additional criterion (the p-gain) had to be fulfilled next to a significant p-value. The p-gain is defined as the fold decrease in the p-value of association for the pair of metabolites compared to the lowest of the two p-values for the single metabolites [6]. Thus, an association between a metabolite ratio (M1/M2) and FFMI is considered to be significant, if the p-value of this association is significant and the p-gain exceeds a certain value. To calculate the p-gain we first have to determine the minimum p-value of the associations between metabolite M1 and FFMI as well as metabolite M2 and FFMI. The p-gain is the quotient of this minimum to the p-value of the association between the metabolite ratio and FFMI. This p-gain had to exceed 170 for KORA S4 and 150 for KORA F4. These cut-offs are seen as Bonferroni-related corrections in order to identify metabolite concentration pairs for which the strength of association improves considerably by using ratios [6].

#### Stratified analyses

Different sub-analyses were performed. First, a different main explanatory variable was used. Instead of FFMI, the appendicular skeletal muscle mass index was included in the linear regression models with the same adjustments. Second, stratified analyses were conducted with age, sex, sports activity and BMI as potential confounders or effect modifiers.

#### GGMs

In addition to the linear regression analyses, we investigated the relationships between metabolites as well as the propagation of FFMI effects through the metabolic network by means of Gaussian graphical modelling. In order to obtain a GGM, the partial correlation coefficients between all pairs of metabolites were calculated [12]. For KORA S4 each partial correlation coefficient was controlled for age, sex, FFMI, and the other 164 metabolites and tested for significance. Bonferroni correction was applied, maintaining a significance level of alpha = 0.01. In order to focus on particularly strong effects between metabolites a cut-off of r = 0.3 (partial correlation coefficient) was applied to the network. Each node represents a metabolite, whereas edges represent significant partial correlations. Nodes were coloured according to the β-estimate and the p-value from the linear models (red  =  positive estimate; blue  =  negative estimate; white  =  not significant estimate). The same procedures were applied to the KORA F4 metabolite concentrations including the batch variable as a confounder.

#### Genetic analyses

The associations between serum metabolite concentrations or ratios and SNPs which are found to be associated with anthropometric characteristic were analysed using linear models with the assumption of additive genetic effects. For the metabolite concentrations of the genotyped KORA S4 subjects (n = 668), models were adjusted for age and sex. For the analysis with KORA F4 metabolic data (n = 890), adjustments were made for age, sex and batch. A p-value of 4.73×10^−06^ for S4 and 5.3×10^−06^ for F4 was considered as statistically significant at α = 5%.

All statistical analyses were performed with the R software version 2.12.0 [R Development Core Team, 2010, http://www.r-project.org]. The GGMs were visualised by the yEd graph editor version 3.6.1.1 [yWorks Gmbh, http://www.yWorks.com].

## Results

**Figure 1:**
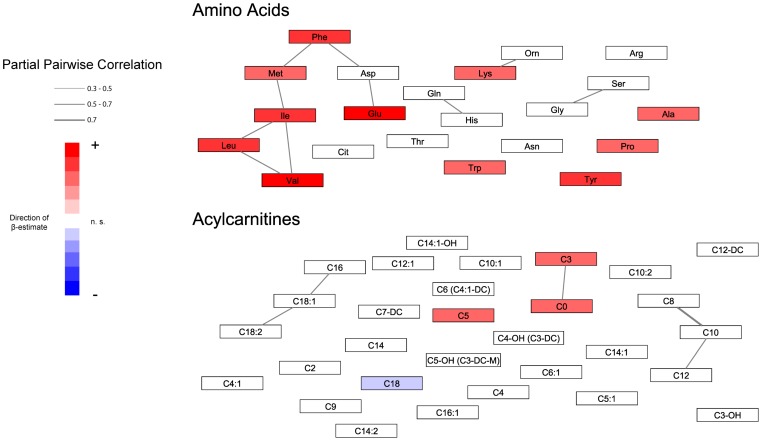
Gaussian graphical model of serum amino acids and acylcarnitine metabolite concentrations in KORA S4. Each node represents a metabolite, whereas edges represent significant partial correlations. Nodes were coloured according to the β-estimate and the p-value from the linear models (red  =  positive association with FFMI; blue  =  negative association with FFMI; white  =  not significant association with FFMI).

**Figure 2:**
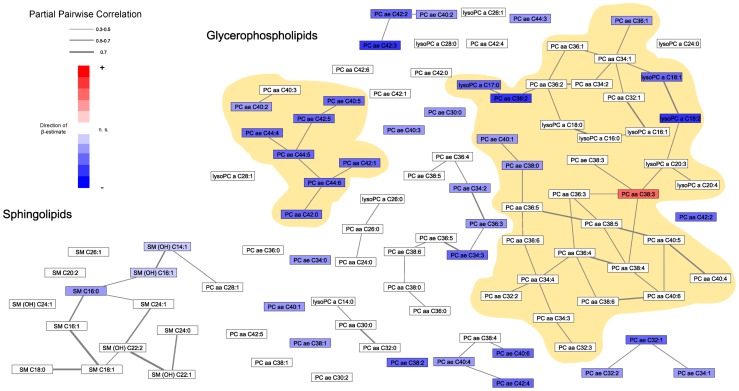
Gaussian graphical model of serum glycerophospholipids and sphingolipid metabolite concentrations in KORA S4. Each node represents a metabolite, whereas edges represent significant partial correlations. Nodes were coloured according to the β-estimate and the p-value from the linear models (red  =  positive association with FFMI; blue  =  negative association with FFMI; white  =  not significant association with FFMI). Yellow highlights both large clusters within the glycerophospholipid metabolites.

Table 1 summarises anthropometric data of the KORA S4 and F4 participants. Selected statistically significant associations of FFMI with different metabolite concentrations for the KORA S4 population are given in Table 2. Different parameters such as the direction of the beta estimate derived from the adjusted linear model are given, as is the agreement between the KORA S4 and F4 results. The full list of statistically significant associations obtained in KORA S4 and F4 can be found in Tables S2 and S3, respectively. The GGMs established in KORA S4 and F4 are displayed in Figure 1, 2, and S1, completing the results of the linear regression models by illustrating the underlying relationships between the metabolite concentrations.

**Table 1:** Characteristics (mean ± SD; % or n absolute) of male and female subjects in the analysed samples of KORA S4 and F4.

		KORA S4 (n = 965)		KORA F4 (n = 890)	
		Men	Women	Men	Women
		(n = 485)	(n = 480)	(n = 423)	(n = 467)
Parameter	Unit	mean ± SD	mean ± SD	mean ± SD	mean ± SD
Age	years	63.21±5.53	63.34±5.30	53.82±12.02	54.66±12.48
Weight	kg	83.19±11.36	71.59±12.03	83.19±11.37	69.04±13.11
Height	cm	172.26±6.35	159.33±5.96	176.69±7.03	162.86±6.58
Body Mass Index	kg/m^2^	28.04±3.61	28.23±4.68	26.64±3.25	26.04±4.72
Waist	cm	100.01±9.39	89.60±11.05	95.89±9.91	85.19±12.63
Hip	cm	104.70±6.49	107.19±9.90	104.17±6.15	104.19±9.37
Waist to Hip Ratio		0.95±0.05	0.83±0.06	0.92±0.06	0.82±0.07
Fat Free Mass^a^	kg	58.48±5.74	43.11±5.06	60.82±6.05	43.58±5.39
Body Fat Mass^a^	%	29.25±4.66	39.19±4.79	26.32±4.95	35.95±5.66
FFMIa, b	kg/m^2^	19.71±1.66	16.99±1.85	19.59±1.55	16.50±1.76
BFMIa, c	kg/m^2^	8.34±2.29	11.24±3.08	7.17±2.07	9.61±3.15
Parameter	Category	% (n absolute)	% (n absolute)	% (n absolute)	% (n absolute)
Body Mass Index	<18.5	0 (0)	0 (0)	0.24 (1)	0.64 (3)
	18.5–<25	17.11 (83)	25.62 (123)	30.50 (129)	45.82 (214)
	25–<30	56.49 (274)	43.33 (208)	52.72 (223)	34.90 (163)
	≥30	26.19 (127)	30.63 (147)	16.55 (70)	18.63 (87)
Sports Activity^d^	active	38.76 (188)	46.67 (224)	59.81 (253)	61.24 (286)
	inactive	60.41 (293)	52.92 (254)	40.00 (169)	38.76 (181)
Age Groups	31–40 y	–	–	14.66 (62)	15.42 (72)
	41–50 y	–	–	29.55 (125)	24.2 (113)
	51–60 y	38.14 (185)	35.42 (170)	23.88 (101)	26.98 (126)
	61–70 y	48.45 (235)	52.08 (250)	21.99 (93)	21.2 (99)
	71–80 y	13.4 (65)	12.5 (60)	9.46 (40)	11.56 (54)
	≥81 y	–	–	0.47 (2)	0.64 (3)

a Parameters derived from the bioelectrical impedance analysis measurements which were only conducted in KORA S4;

b Fat Free Mass Index;

c Body Fat Mass Index;

d Sports (active: >1 h leisure time of sports per week on a regular basis; inactive: less than 1 h sports per week).

**Table 2:** Selected metabolic traits significantly associated with FFMI^a^ in a linear regression model adjusted for age and sex in the KORA S4 sample.

Trait	Mean	SD	Dir.^b^	adj. P-value^c^	R^2^ adj.^d^	P-gain^e^	F4^f^
	(µmol/l)						
Val	227.26	53.13	pos.	4.75×10^−16^	0.16		*
Glu	80.13	32.04	pos.	1.22×10^−15^	0.11		n.a.
Ile	72.14	20.22	pos.	1.96×10^−11^	0.22		#
Leu	160.51	44.33	pos.	2.57×10^−08^	0.18		#
Ala	417.99	101.4	pos.	8.95×10^−06^	0.03		n.a.
Tyr	72.09	20.06	pos.	4.77×10^−10^	0.06		*
Phe	76.73	17.19	pos.	2.53×10^−07^	0.05		n.s.
Σ aromatic AAs	208.98	43.75	pos.	3.37×10^−09^	0.07		*
ΣBCAAs	459.91	113.06	pos.	2.07×10^−13^	0.19		*
Ile/Gly	0.29	0.11	pos.	1.61×10^−14^	0.30	1.22×10^+03^	#
Leu/Gly	0.65	0.25	pos.	1.46×10^−12^	0.29	1.75×10^+04^	#
Σ BCAAs/Σ glucogenic AAs	0.58	0.14	pos.	1.49×10^−08^	0.26		*
C5	0.16	0.06	pos.	3.49×10^−05^	0.17		*
C3	0.47	0.15	pos.	4.85×10^−05^	0.13		*
C0	40.52	8.49	pos.	1.19×10^−02^	0.13		*
C18	0.06	0.01	neg.	3.96×10^−02^	0.10		n.s.
C18/C5	0.37	0.14	neg.	1.44×10^−09^	0.08	2.42×10^+04^	n.s.
C18/C3	0.13	0.05	neg.	3.65×10^−09^	0.05	1.33×10^+04^	n.s.
C18/C0	0.001	0.0004	neg.	5.44×10^−07^	0.05	2.19×10^+04^	n.s.
PC aa C38:3	57.77	14.01	pos.	7.02×10^−06^	0.06		*
PC ae C42:3	0.85	0.19	neg.	5.10×10^−18^	0.10		*
PC ae C36:2	15.33	3.88	neg.	2.42×10^−15^	0.19		*
lysoPC a C18:2	28.46	9.04	neg.	8.19×10^−16^	0.16		*
lysoPC a C18:1	21.61	6.1	neg.	2.30×10^−10^	0.11		*
Σ PC ae	181.45	30.52	neg.	1.38×10^−04^	0.10		*
Σ lysoPC	229.29	47.21	neg.	4.36×10^−04^	0.07		*
PC aa C38:3/PC aa C42:6	97.71	21.87	pos.	1.04×10^−17^	0.09	6.73×10^+11^	*
PC aa C38:3/PC aa C42:1	228.35	80.97	pos.	6.09×10^−15^	0.07	1.15×10^+07^	*
PC aa C38:3/PC aa C42:0	111.66	41.58	pos.	1.06×10^−15^	0.07	3.68×10^+06^	*
PC aa C38:3/PC aa C42:2	297.65	96.51	pos.	1.72×10^−16^	0.10	1.69×10^+09^	*
lysoPC a C14:0/lysoPC a C18:2	0.24	0.08	pos.	1.04×10^−20^	0.18	7.88×10^+04^	n.s.
PC ae C36:4/PC ae C40:1	12.99	3.34	pos.	1.70×10^−07^	0.04	3.57×10^+02^	*
PC aa C38:3/lysoPC a C18:1	2.27	0.99	pos.	3.37×10^−20^	0.19	2.43×10^+04^	*
PC aa C38:3/lysoPC a C18:2	2.87	1.03	pos.	1.38×10^−19^	0.17	1.66×10^+09^	*
PC aa C38:3/PC ae C42:3	70.43	21.88	pos.	3.12×10^−26^	0.12	1.63×10^+08^	*
PC aa C38:3/PC ae C36:2	3.98	1.36	pos.	8.17×10^−25^	0.13	2.96×10^+09^	*
PC aa C38:3/PC ae C42:2	93.08	24.28	pos.	3.73×10^−23^	0.11	7.97×10^+15^	*
SM C16:0/SM C16:1	6.4	0.76	neg.	1.52×10^−11^	0.29	3.13×10^+08^	*
SM (OH) C16:1/SM C18:1	0.31	0.06	neg.	3.51×10^−10^	0.05	4.94×10^+07^	*
SM C16:0/SM C18:1	9.36	1.83	neg.	5.71×10^−08^	0.24	8.33×10^+04^	*
SM (OH) C16:1/SM C18:0	0.16	0.03	neg.	3.28×10^−10^	0.10	5.27×10^+07^	*
SM (OH) C22:2/SM C18:1	1.00	0.19	neg.	4.14×10^−07^	0.05	2.93×10^+05^	*

a Fat Free Mass Index;

b direction of the association (positive or negative);

c for multiple testing adjusted p-value;

d adjusted R^2^ of the linear model;

e p-gain, fold decrease in the p-value of association for the pair of metabolites, compared to the lowest of two p-values for the single metabolites;

f confirmed in KORA F4, for * significance and direction; # confirmed for xLeu in F4, n.a., metabolite was not available in F4; AAs, amino acids; Σ aromatic amino acids, sum of tyrosine, phenylalanine, and tryptophan; Σ BCAAs, sum of valine, isoleucine, and leucine; Σ glucogenic amino acids, sum of alanine, glycine, and serine.

The full results are given in Tables S2 (KORA S4) and S3 (KORA F4).

### Amino Acids

**Figure 3:**
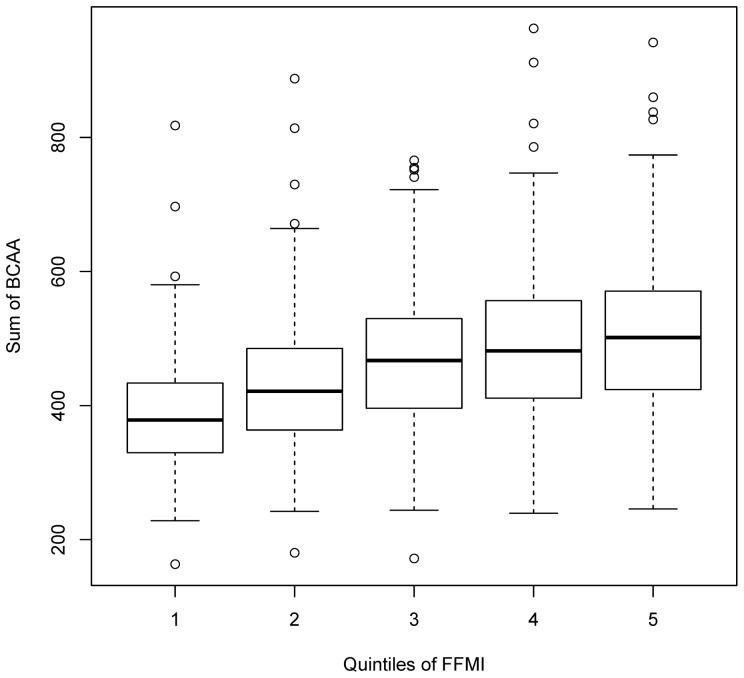
Boxplot of serum BCAA concentrations (µmol/l), by quintiles of the FFMI, for the KORA S4 population.

With higher FFMI, increasing serum concentrations of the branched chained amino acids (BCAAs) valine, isoleucine, and leucine as well as of the sum of BCAAs were observed. Furthermore, other serum amino acids increased with higher FFMI, including the glucogenic amino acid alanine, and the aromatic amino acids tyrosine and phenylalanine. With respect to metabolite ratios, strong positive associations were found between FFMI and the ratios of isoleucine/glycine and leucine/glycine. In addition, the ratio of all BCAAs/all glucogenic amino acids (sum of alanine, glycine, and serine) was positively related to FFMI, supporting the notion of increasing BCAAs concentrations in relationship to glucogenic amino acids in serum samples of subjects with higher FFMI. Inspecting the GGM results (Figure 1), we also observe these strong correlations between BCAAs. Based on these results, we illustrated the association between the sum of BCAAs and FFMI (by FFMI quintiles) in Figure 3, and described the anthropometric characteristics of the S4 participants by BCAAs quintiles (Table 3). We were able to replicate these findings in the KORA F4 sample (Table S3).

**Table 3:** Different characteristics of the KORA S4 population stratified by quintiles of the serum BCAA^a^ concentration, given as mean and SD as well as percentage and absolute quantity.

	Quintiles of the sum of BCAAs				
Quintile	1	2	3	4	5
(µmol/l)	(163.5–363.7)	(364.1–418)	(418.2–476.9)	(477–542.2)	(542.3–963)
Age	63.08±5.41	63.27±5.38	63.5±5.62	63.6±5.42	62.94±5.29
Weight	69.45±11.77	75.5±2.72	76.11±11.18	81.64±12.39	84.32±11.93
Height	161.35±7.48	164.65±8.73	165.77±9.13	168.86±8.77	168.51±8.38
Body Mass Index	26.64±4.08	27.85±4.27	27.75±4	28.63±3.75	29.79±4.15
Fat Free Mass (kg)	44.13±7.34	49.41±9.08	50.19±8.72	54.75±8.83	55.72±8.12
Body Fat Mass (%)	36.1±6.34	34.37±6.88	34±7.15	32.73±6.82	33.74±6.68
FFMI^b^	16.87±2.02	18.11±2.25	18.15±2.05	19.09±1.89	19.55±1.89
BFMI^c^	9.77±2.88	9.74±3.08	9.6±3.15	9.54±2.97	10.25±3.24
FFM/BFM^d^	1.89±0.81	2.05±0.73	2.08±0.69	2.19±0.67	2.09±0.66
Male	19.69% (38)	42.49% (82)	49.74% (96)	67.88% (131)	71.5% (138)
Female	80.31% (155)	57.51% (111)	50.26% (97)	32.12% (62)	28.5% (55)
Sports activity^e^	47.15% (91)	35.75% (69)	52.33% (101)	40.93% (79)	37.31% (72)

a BCAAs, sum of valine, isoleucine, and leucine;

b Fat Free Mass Index;

c Body Fat Mass Index;

d Fat Free Mass divided by Body Fat Mass;

e Sports (active: >1 h leisure time of sports per week on a regular basis; inactive: less than 1 h sports per week).

### Acylcarnitines

Serum concentrations of free carnitine and short-chain odd-numbered acylcarnitines, such as propionylcarnitine (C3) and valerylcarnitine (C5), were found to be positively associated with FFMI while octadecanoylcarnitine (C18) decreased with increasing FFMI. This is also reflected in the results for the ratios of C18 to C0, C3, or C5. These findings in S4 are supported by the KORA F4 results. The acylcarnitines form a separate group within the partial correlation networks and a particularly strong correlation exists between the metabolites C0 and C3.

### Phosphatidylcholines

The group of phosphatidylcholines consists of different PC diacyl (aa), PC acyl-alkyl (ae), and lysoPC acyl (a) compounds. Numerous associations between FFMI and PCs or PC ratios could be observed. An increase in FFMI was associated with (i) a higher serum concentration of PC aa in relation to PC ae, (ii) a decrease in chain length of the fatty acid residues, and (iii) a decrease in saturation (i.e., a higher number of double bonds) of the fatty acid moieties. Significant results for single PC compounds were all negatively associated with FFMI, such as PC ae C42:3 or PC ae C36:2, lysoPC a C18:2 or lysoPC a C18:1; the only exception is PC aa C38:3 which increased with higher FFMI. Ratios within subgroups (aa/aa, ae/ae, lyso/lyso) demonstrated that increasing FFMI is associated with a shift towards shorter fatty acids and fatty acids with more double bonds. Examples are ratios of PC aa C38:3/PC aa C42:6, PC aa C38:3/PC aa C42:2, PC aa C38:3/PC aa C42:0, PC ae C36:4/PC ae C40:1 or lysoPC a C14:0/lysoPC a C18:2. This is most likely a consequence of the associations observed for the single compounds (e.g., PC aa C38:3 increased and lysoPC a C18:1 decreased with increasing FFMI). Ratios of PC aa to PC ae were significantly associated with FFMI, e.g., PC aa C38:3/PC ae C42:3 or PC aa C38:3/PC ae 42:2.

There are two very large groups of PCs which were identified in the network analyses (Figure 2). PC aa C38:3 is the centre of one cluster which includes mainly PC aa and shorter PC ae; the other cluster consists of mostly long-chain PCs.

These findings are also supported by the results obtained in the KORA F4 sample.

### Sphingomyelins

With higher FFMI an increased concentration of sphingomyelins (SM) as compared to hydroxysphingomyelins (SM (OH)) was observed. This is reflected e.g., by the ratios of SM (OH) C16:1/SM C18:1, SM (OH) C16:1/SM C18:0 or SM (OH) C22:2/SM C18:1. Also, the results of SM ratios, such as SM C16:0/SM C16:1 or SM 16:0/SM C18:1, demonstrate a decrease in saturation of the fatty acids with increasing FFMI. The same associations are found in KORA F4. The GGM results show that almost all SM are interrelated and the strong relationship between SM (OH) C16:1 and SM (OH) C14:1 is present in both GGMs (Figure 2 and Figure S1).

### Appendicular Skeletal Muscle Mass Index

For both, KORA S4 and F4, the results for the appendicular skeletal muscle mass index were largely comparable to the results with FFMI (data not shown). However, the p-values in the linear regression models did not decrease and also the adjusted R^2^ did not improve as compared to the FFMI-based results. Therefore, FFMI remained as primary explanatory variable.

### Stratifications

In stratified analyses, very similar results could be observed for men and women. With respect to sports activity, there was only a slight difference between both groups (active versus inactive participants) for the acylcarnitine metabolites. Most of the statistically significant associations with FFMI and the acylcarnitines were not present in the inactive group. Regarding age (>65 years vs. ≤65 years; S4), there were no distinct differences between groups.

With respect to the associations between FFMI and the metabolites in obese and non-obese subjects in KORA S4 we did observe significant differences. Most associations described for the whole population and also noted for the non-obese subgroup are not present in obese subjects (Table 4).

**Table 4:** Selected metabolic traits significantly associated with FFMI^a^ in a linear regression model adjusted for age, and sex for the non-obese and obese participants in the KORA S4 sample.

	Obese (BMI ≥30 kg/m^2^)(n = 274)			Non-obese (BMI<30 kg/m^2^)(n = 691)		
	beta	adj. P-value^b^	R^2^ adj.^c^	beta	adj. P -value^b^	R^2^ adj.^c^
Val	0.08	1.00	0.03	0.14	1.70×10^−04^	0.17
Ile	0.06	1.00	0.12	0.13	3.94×10^−04^	0.25
Leu	0.04	1.00	0.08	0.13	3.77×10^−04^	0.22
Ala	0.02	1.00	0.00	0.12	0.0409	0.02
Tyr	0.04	1.00	0.00	0.14	2.09×10^−03^	0.05
Phe	0.06	1.00	0.00	0.10	0.2377	0.04
Σ aromatic AAs	0.05	1.00	0.02	0.12	0.014	0.06
Σ BCAAs	0.06	1.00	0.07	0.15	5.10×10^−05^	0.21
Ile/Gly	0.03	1.00	0.26	0.13	2.42×10^−04^	0.30
Leu/Gly	0.02	1.00	0.22	0.13	9.29×10^−05^	0.30
Σ BCAAs/glucogenic AAs	0.05	1.00	0.15	0.10	0.0353	0.30
C5	0.06	1.00	0.13	0.14	1.34×10^−04^	0.19
C3	0.06	1.00	0.06	0.13	2.52×10^−03^	0.16
C0	0.06	1.00	0.08	0.09	0.255	0.16
PC aa C38.3	0.00	1.00	0.02	0.08	1.00	0.04
PC ae C42.3	−0.14	0.31	0.04	−0.16	7.30×10^−05^	0.06
PC ae C36.2	−0.07	1.00	0.13	−0.09	0.418	0.17
lysoPC a C18.1	−0.08	1.00	0.08	−0.07	1.00	0.05
lysoPC a C18.2	−0.14	0.21	0.13	−0.07	1.00	0.08
Σ PC ae	−0.04	1.00	0.05	−0.05	1.00	0.09
Σ lysoPC	−0.04	1.00	0.06	−0.02	1.00	0.04
PC aa C38:3/PC aa C42:6	0.03	1.00	0.01	0.18	2.69×10^−06^	0.05
PC aa C38:3/PC aa C42:2	0.05	1.00	0.03	0.14	7.49×10^−04^	0.05
lysoPC a C14:0/lysoPC a C18:2	0.18	0.003	0.17	0.10	0.113	0.09
PC aa C38:3/lysoPC a C18:1	0.07	1.00	0.11	0.11	0.0377	0.10
PC aa C38:3/lysoPC a C18:2	0.10	1.00	0.13	0.10	0.158	0.11
PC aa C38:3/PC ae C42:3	0.10	1.00	0.01	0.18	8.77×10^−07^	0.05
PC aa C38:3/PC ae C36:2	0.06	1.00	0.04	0.14	1.48×10^−03^	0.06
PC aa C38:3/PC ae C42:2	0.07	1.00	0.00	0.17	1.02×10^−05^	0.04
SM C16:0/SM C16:1	−0.02	1.00	0.23	−0.06	1.00	0.27
SM (OH) C16:1/SM C 18:1	0.01	1.00	0.01	−0.08	1.00	0.02
SM C 16:0/SM C 18:1	−0.02	1.00	0.17	−0.06	1.00	0.23
SM (OH) C16:1/SM C 18:0	0.02	1.00	0.17	−0.10	0.170	0.05
SM (OH) C22:2/SM C 18:1	0.00	1.00	0.05	−0.07	1.00	0.01

a Fat Free Mass Index;

b for multiple testing adjusted p-value;

c adjusted R^2^ of the linear model; AAs, amino acids; Σ aromatic amino acids, sum of tyrosine, phenylalanine, and tryptophan; Σ BCAAs, sum of valine, isoleucine, and leucine; Σ glucogenic amino acids, sum of alanine, glycine, and serine.

### Genetic Analyses

No significant associations were found between the selected genetic variants (Table S4) and the serum metabolite concentrations (KORA S4 or KORA F4).

## Discussion

In this population-based study, strong associations between FFMI and serum metabolite concentrations were found in KORA S4 and reproduced in KORA F4. With higher FFMI, BCAA serum concentrations and the ratio of BCAAs to glucogenic amino acids increased. Free carnitine levels were also positively associated with FFMI, and for the various PCs we found a decrease in chain length and/or saturation of the fatty acid residues in combination with higher concentrations of PC aa in expense of PC ae and lysoPC. In obese subjects of KORA S4, however, these associations were lacking.

The human serum metabolome is currently characterised in many studies using different analytic approaches [4], including the method applied here. To the best of our knowledge, the association of FFMI/fat free mass and serum metabolites was not explored before in an epidemiologic setting. Rather, metabolomics signatures of exercise (before, during and afterwards) in human plasma were investigated showing that subjects who were in better shape exhibited more lipolysis during and after exercise than did the less fit participants [20]. This leads to the hypothesis that a sedentary lifestyle leads to derangements of skeletal muscle metabolism that favour the development of obesity and metabolic diseases. In fact, our observation that associations found between FFMI and serum metabolites were largely missing in obese subjects at baseline (S4) fits well to the hypothesis of a deranged skeletal muscle metabolism. As observed by Lewis et al. [20], a decreased capability to stimulate lipid oxidation during exercise could be reflected in the missing associations between FFMI and the various lipid metabolites in obese subjects. For the non-obese participants, we find evidence for an enhanced ß-oxidation with higher FFMI which is reflected in the carnitine and PC metabolite results.

In contrast to a study with physical activity intervention, it was not clear how strong associations between FFMI and serum concentrations of amino acids, acylcarnitines, glycerophospholipids, and sphingolipids could be in a cross-sectional study with collection of serum samples after short-term fasting of at least eight hours. However, our results fit well to biological pathways that are related to skeletal muscle metabolism, namely amino acid metabolism and fatty acid metabolism (reflected by acylcarnitines and glycerophospholipids).

BCAAs are described as a preferred substrates for muscle tissue, and BCAA supplementation can suppress protein degradation [21]. During short-term fasting, glucose released from glycogen stores of the liver is provided as fuel for tissues that need glucose (e.g. brain). Muscle protein degradation provides amino acids that are used by the liver for gluconeogenesis [22], with alanine being the most important one. BCAAs are a major source of nitrogen for muscle synthesis of glucogenic amino acids such as alanine [23], [24]. This has been shown in studies with 1–3 days of starvation. Levels of BCAAs rise in fasting state parallel to an increased protein degradation. In this state, the only source of BCAAs is appearance from protein degradation, which is a key process for maintenance of protein quality and repair process of tissues. Thus, during overnight fasting, BCAA serum concentrations are increased proportional to the muscle mass of the body. Furthermore, alanine concentration levels rise with increasing protein degradation during starving [21]. Thus, our findings of increasing serum concentrations of BCAAs and alanine that are strongly associated with FFMI are in line with the expectations.

Several other studies reported that acylcarnitines are also found in circulation [4]. Acylcarnitines are intra-cellularly synthesized for the transport of fatty acids into the mitochondria for β-oxidation. In the present analyses an increase of acylcarnitine concentrations could be observed which argues for an upregulated β-oxidation of fatty acids in subjects with higher muscle mass. One explanation for the origin of propionylcarnitine is oxidative degradation (β-oxidation, γ-oxidation) of BCAAs, releasing propionylcarnitine units. Another source of propionyl residues may be the intestinal production by gut microbiota; however a relationship with plasma propionylcarnitine or FFMI is not established.

Our phospholipid results point towards a decrease in chain length and degree of saturation of the lipid side chains. The concentrations of lysoPC and PC ae decrease with increasing FFMI and PC aa C38:3 is the only PC positively associated with higher FFMI. Considering the GGM results, PC aa C38:3 is central in one cluster of PC metabolites, while the other cluster consists of long chain PCs. Overall, our results implicate that FFMI is associated with lower plasma concentrations of very long chain PCs (GGM cluster 2), while the concentration of PC aa C38:3 increased. Thus, a higher FFMI might indicate a higher activity of enzymes involved in fatty acid oxidation, especially in the oxidation of very-long chain fatty acids. In addition, an altered activity of enzymes involved in chain elongation (ELOVL) or desaturation (FADS) could contribute to these findings. Earlier studies using metabolomics and genetic data could demonstrated that genetic effects (mediated by the expression of enzymes) are reflected in the serum metabolic profile, thus lending credibility to the approach of identifying enzyme activities by means of serum metabolites or ratios of serum metabolites [15], [25]. The shift from PC ae and lysoPC towards PC aa could also be explained by modifications in expression or activity of relevant enzymes [15]. However, analyses of the association between selected SNPs which are known to be associated with anthropometric characteristics did not reveal any significant relationship with metabolite ratios.

In addition, a higher FFMI was found to be associated with an increased concentration of sphingomyelins as compared to hydroxysphingomyelins. Recently, Wang-Sattler et al. [26] reported a reduced concentration of SM and an increased concentration of SM (OH) in smokers. This finding was explained as a consequence of smoking on the activity of the peroxisomal enzyme alkylglycerone phosphate synthase (alkyl-DHAP). Following the same line of argumentation, FFMI is probably associated with an increase of the activity of alkyl-DHAP. The negative association of the SM C16∶0/SM C16∶1 ratio with FFMI might be explained by regulatory processes in the fatty acid biosynthesis pathway. In particular, stearoyl-CoA desaturase (SCD), which catalyses the desaturation of C16 and C18 fatty acids, might be a target enzyme/gene affected by greater muscle mass and/or activity.

### Strength and Limitations

The participants of our studies represent random samples of the underlying population. In such a population with a heterogeneous metabolic makeup influenced by different environmental factors, genetic predispositions, and lifestyles, the interpretation of metabolic profiling is quite ambitious [6]. The metabolite concentrations which are reported in the present paper were identified by means of two slightly different Ablsolute*IDQ*™ kits p150 and p180. These two kits are not identical (190 different metabolites were measured with an overlap of 141) and the measurement method of the amino acids slightly differs. However, this technology allows the automated quantification of hundreds of metabolites for many samples at a time which is very helpful for future studies [27]. Furthermore, the side chain composition of the different PCs, lysoPCs, sphingomyelins and acylcarnitines carries many information on the different fatty acid pools in the human body, such as n−3 and n−6 polyunsaturated fatty acids or saturated and mono-saturated short and medium chain fatty acids [28]. Thus, the kit is well oriented to map out the human lipidome and therefore particular for the purpose of this study.

The bioelectrical impedance analysis measurements did only take place in KORA S4. For the analyses of associations between FFMI and the metabolite concentrations in KORA F4 only the results of the bioelectrical impedance analysis measurements at baseline examination (KORA S4) were available. Assuming that the participants’ body composition is fairly stable in weight-stable subjects, the KORA F4 based analyses were restricted to the weight-stable subjects in order to account for this.

For the current analysis, subjects with a diagnosis of diabetes or hypertension or medication for both diseases were excluded. However, obese subjects are more likely to develop derangements in glucose or lipid metabolism as compared to normal-weight subjects which are in a subclinical state and have not led to a clinical diagnosis yet. An analysis of serum glucose (hexose) and triacylglycerol indicated significantly different means between non-obese and obese subjects. In non-obese subjects, mean ± SD serum glucose concentration was 5.18±0.76 mmol/l and mean serum triacylglycerol concentration was 1.4±0.81 mmol/l; the corresponding values in obese subjects were 5.6±1.11 mmol/l and 1.70±1.05 mmol/l, respectively. However, the higher glucose and triacylglycerol concentrations in obese do not indicate a catabolic status and thus are not sufficient to explain the missing association between FFMI and serum metabolites.

Stratified analyses by gender, age, and sports activity were conducted in order to detect potential confounders and effect modifiers. Similar results were found for men and women separately. P-values increased, as the sample size within the strata (slightly more for men) was smaller, but there was no improvement of the model fit, represented by the adjusted R^2^. In addition, the FFMI is independent of height [3] thus taking one difference between men and women into account. Furthermore, analysing the whole sample increases the power and we were able to cover a wider range of the FFMI. With respect to sports activity, slight differences were found for the acylcarnitine metabolite group. There, these effects were no longer present within the inactive subjects. However, as sports activity and FFMI are correlated and FFMI covers the long-term sports effect as it represents the muscle mass, sports activity was not included as a covariate in the main analyses.

The GGMs present low levels of linkage between the metabolites which is in line with findings from Krumsiek et al. [12] who demonstrated that GGMs omit indirect correlations. Nevertheless, the GGMs complete the linear regression results with the underlying relationships between the metabolites.

### Conclusion

We found strong associations between serum amino acids, acylcarnitines, and glycerophospholipids with the FFMI in a population-based sample. These findings were stable as they could be reproduced in a follow-up study of this population. Most interestingly, such associations were largely missing in obese subjects. The latter finding supports the hypothesis that a sedentary lifestyle associated with accumulation of fat tissue may be accompanied by a derangement in skeletal muscle metabolism, especially a limited inducibility of fatty acid oxidation.

## Supporting Information

Figure S1 **Gaussian graphical model of serum metabolite concentrations of KORA F4.** Each node represents a metabolite, whereas edges represent significant partial correlations. Nodes were coloured according to the β-estimate and the p-value from the linear models (red  =  positive association with fat free mass index; blue  =  negative association with fat free mass index; white  =  not significant association with fat free mass index).
(TIF)

Table S1 **Full biochemical names, abbreviation, mean ± standard deviation of all metabolite concentrations measured in µmol/l with the Biocrates Absolute** ***IDQ*** **kits p150 (KORA F4, n = 890) and p180 (KORA S4, n = 965).**
(DOC)

Table S2 **Metabolic traits significantly associated with fat free mass index in linear regression models adjusted for age, and sex (α = 5%, p-gain>170) in the KORA S4 sample.**
(DOC)

Table S3 **Metabolic traits significantly associated with fat free mass index in linear regression models adjusted for age, sex and batch (α = 5%, p-gain>150) in the KORA F4 weight-stable sample.**
(DOC)

Table S4 **List of selected SNP, which were significantly associated in genome wide association studies with anthropometric characteristic as outcomes and tested in the present study for associations with metabolomics data.**
(DOC)
