# Supplementary figures and images for: Body Fat Free Mass Is Associated with the Serum Metabolite Profile in a Population-Based Study
Source: PLoS One. 2012 Jun 27;7(6):e40009. doi: 10.1371/journal.pone.0040009 (PMC3384624; doi:10.1371/journal.pone.0040009)

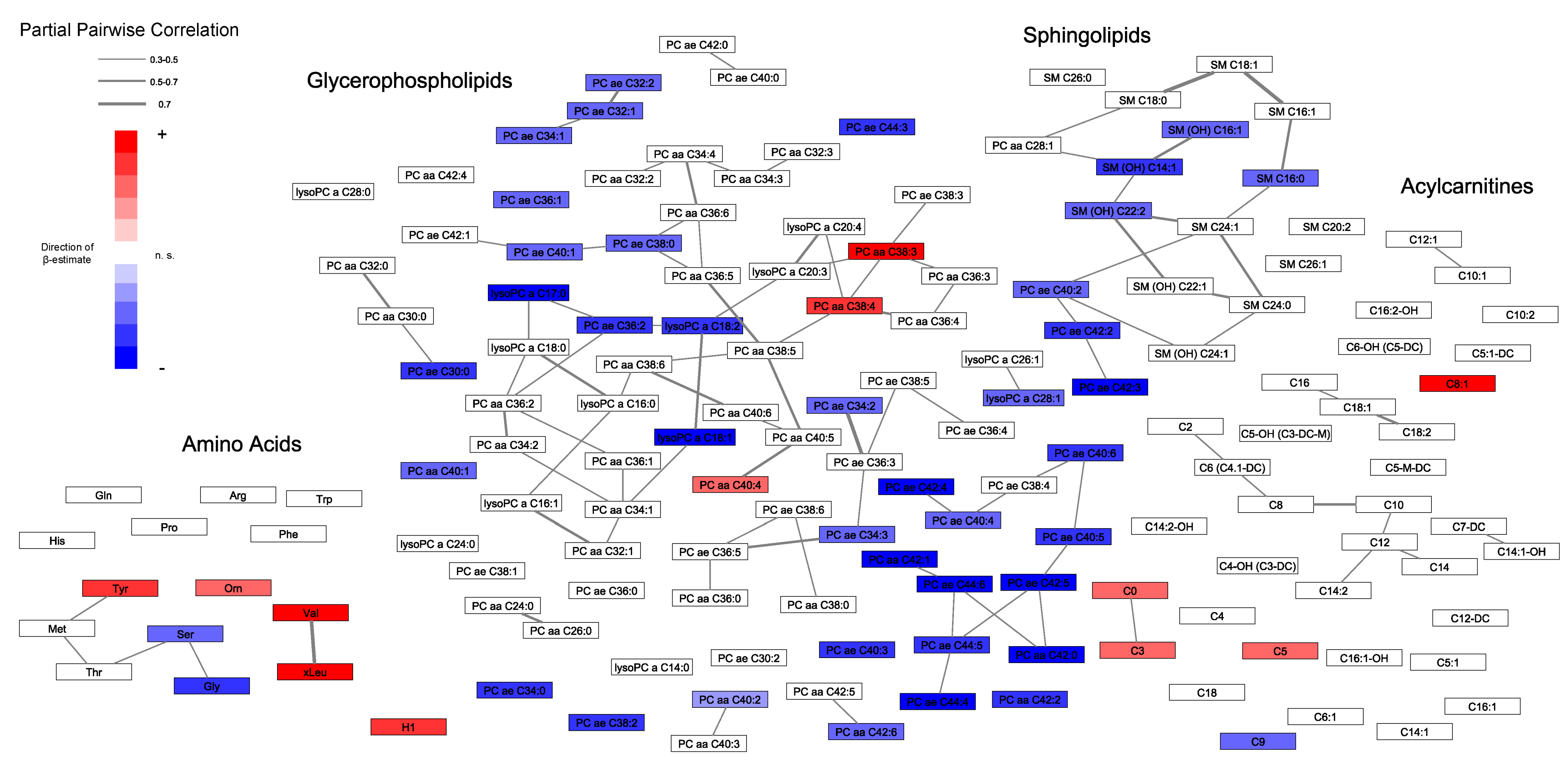

Supplement: Figure S1 — Gaussian graphical model of serum metabolite concentrations of KORA F4. Each node represents a metabolite, whereas edges represent significant partial correlations. Nodes were coloured according to the β-estimate and the p-value from the linear models (red = positive association with fat free mass index; blue = negative association with fat free mass index; white = not significant association with fat free mass index). (TIF) [file pone.0040009.s001.tif]
