# Supplementary material for: Body Fat Free Mass Is Associated with the Serum Metabolite Profile in a Population-Based Study
Source: PLoS One. 2012 Jun 27;7(6):e40009. doi: 10.1371/journal.pone.0040009 (PMC3384624; doi:10.1371/journal.pone.0040009)
Supplement: Table S1 — Full biochemical names, abbreviation, mean ± standard deviation of all metabolite concentrations measured in µmol/l with the Biocrates Absolute IDQ kits p150 (KORA F4, n = 890) and p180 (KORA S4, n = 965). (DOC) [file pone.0040009.s002.doc]

**Table S1**: Full biochemical names, abbreviation, mean ± standard deviation of all metabolite concentrations measured in µmol/l with the Biocrates Absolute*IDQ* kits p150 (KORA F4, n=890) and p180 (KORA S4, n=965).

| Abbrevation | Full biochemical name | Mean ± SD  (KORA S4) | Mean ± SD  (KORA F4) |
| --- | --- | --- | --- |
| C0 | Carnitine | 40.52 ± 8.49 | 35.05 ± 7.36 |
| C2 | Acetylcarnitine | 8.62 ± 2.86 | 8.08 ± 2.78 |
| C3 | Propionylcarnitine | 0.47 ± 0.15 | 0.38 ± 0.12 |
| C3‐OH | Hydroxypropionylcarnitine | 0.12 ± 0.06 | * |
| C3:1 | Propenonylcarnitine | * | * |
| C4 | Butyrylcarnitine | 0.22 ± 0.1 | 0.22 ± 0.09 |
| C4‐OH (C3‐DC) | Hydroxybutyrylcarnitine | 0.06 ± 0.02 | 0.09 ± 0.05 |
| C4:1 | Butenylcarnitine | 0.02 ± 0.01 | * |
| C5 | Valerylcarnitine | 0.16 ± 0.06 | 0.11 ± 0.04 |
| C5‐DC (C6‐OH) | Glutarylcarnitine | * | 0.03 ± 0.01 |
| C5‐M‐DC | Methylglutarylcarnitine | * | 0.03 ± 0.01 |
| C5‐OH (C3‐DC‐M) | Hydroxyvalerylcarnitine (Methylmalonylcarnitine) | 0.03 ± 0.01 | 0.04 ± 0.02 |
| C5:1 | Tiglylcarnitine | 0.05 ± 0.01 | 0.03 ± 0.01 |
| C5:1‐DC | Glutaconylcarnitine | * | 0.02 ± 0.01 |
| C6 (C4:1‐DC) | Hexanoylcarnitine | 0.09 ± 0.03 | 0.07 ± 0.03 |
| C6:1 | Hexenoylcarnitine | 0.02 ± 0.01 | 0.02 ± 0.01 |
| C7‐DC | Pimelylcarnitine | 0.05 ± 0.02 | 0.05 ± 0.02 |
| C8 | Octanoylcarnitine | 0.26 ± 0.1 | 0.22 ± 0.09 |
| C8:1 | Octenoylcarnitine | ~ | 0.09 ± 0.04 |
| C9 | Nonaylcarnitine | 0.04 ± 0.02 | 0.05 ± 0.02 |
| C10 | Decanoylcarnitine | 0.38 ± 0.17 | 0.36 ± 0.15 |
| C10:1 | Decenoylcarnitine | 0.18 ± 0.06 | 0.17 ± 0.06 |
| C10:2 | Decadienylcarnitine | 0.04 ± 0.01 | 0.04 ± 0.01 |
| C12 | Dodecanoylcarnitine | 0.16 ± 0.06 | 0.13 ± 0.05 |
| C12‐DC | Dodecanedioylcarnitine | 0.07 ± 0.01 | 0.06 ± 0.01 |
| C12:1 | Dodecenoylcarnitine | 0.17 ± 0.06 | 0.15 ± 0.05 |
| C14 | Tetradecanoylcarnitine | 0.06 ± 0.02 | 0.05 ± 0.01 |
| C14:1 | Tetradecenoylcarnitine | 0.15 ± 0.05 | 0.15 ± 0.05 |
| C14:1‐OH | Hydroxytetradecenoylcarnitine | 0.02 ± 0.01 | 0.01 ± 0 |
| C14:2 | Tetradecadienylcarnitine | 0.04 ± 0.02 | 0.03 ± 0.01 |
| C14:2‐OH | Hydroxytetradecadienylcarnitine | * | 0.01 ± 0 |
| C16 | Hexadecanoylcarnitine | 0.14 ± 0.03 | 0.12 ± 0.03 |
| C16‐OH | Hydroxyhexadecanoylcarnitine | * | * |
| C16:1 | Hexadecenoylcarnitine | 0.04 ± 0.01 | 0.04 ± 0.01 |
| C16:1‐OH | Hydroxyhexadecenoylcarnitine | * | 0.01 ± 0 |
| C16:2 | Hexadecadienylcarnitine | * | * |
| C16:2‐OH | Hydroxyhexadecadienylcarnitine | * | 0.01 ± 0 |
| C18 | Octadecanoylcarnitine | 0.06 ± 0.01 | 0.05 ± 0.01 |
| C18:1 | Octadecenoylcarnitine | 0.14 ± 0.04 | 0.13 ± 0.04 |
| C18:1‐OH | Hydroxyoctadecenoylcarnitine | * | * |
| C18:2 | Octadecadienylcarnitine | 0.05 ± 0.02 | 0.05 ± 0.01 |
| Ala | Alanine | 417.99 ± 101.4 | ~ |
| Arg | Arginine | 126.87 ± 26.78 | 115.67 ± 17.45 |
| Asn | Asparagine | 46.18 ± 8.31 | ~ |
| Asp | Aspartate | 29.76 ± 9.77 | ~ |
| Cit | Citrulline | 34.64 ± 9.16 | ~ |
| Gln | Glutamine | 581.2 ± 113.95 | 627.31 ± 91.44 |
| Glu | Glutamate | 80.13 ± 32.04 | ~ |
| Gly | Glycine | 264.35 ± 73.19 | 314.88 ± 83.47 |
| His | Histidine | 82.87 ± 14.75 | 99.86 ± 16.47 |
| Ile | Isoleucine | 72.14 ± 20.22 | ~ |
| Leu | Leucine | 160.51 ± 44.33 | ~ |
| Lys | Lysine | 166.44 ± 36.59 | ~ |
| Met | Methionine | 23.86 ± 5.21 | 32.01 ± 5.69 |
| Orn | Ornithine | 58.97 ± 14.83 | 81.5 ± 18.59 |
| Phe | Phenylalanine | 76.73 ± 17.19 | 61.17 ± 10.2 |
| Pro | Proline | 193.96 ± 58.92 | 171.51 ± 51.25 |
| Ser | Serine | 127.36 ± 28.85 | 130.82 ± 24.44 |
| Thr | Threonine | 120.75 ± 31.79 | 107.56 ± 24.41 |
| Trp | Tryptophan | 60.17 ± 12.43 | 83.28 ± 9.85 |
| Tyr | Tyrosine | 72.09 ± 20.06 | 84.6 ± 17.27 |
| Val | Valine | 227.26 ± 53.13 | 273.25 ± 59.21 |
| xLeu | Leucine/Isoleucine | ~ | 210.42 ± 43.19 |
| Ac Orn | Acetylornithine | 0.74 ± 0.41 | ~ |
| ADMA | Asymmetric dimethylarginine ^#^ | 0.56 ± 0.13 | ~ |
| SDMA | Symmetric Dimethylarginine | 0.76 ± 0.24 | ~ |
| total DMA | Sum of ADMA and SDMA | 1.21 ± 0.23 | ~ |
| alpha AAA | alpha-Aminoadipic acid | 0.68 ± 0.27 | ~ |
| Carnosine | Carnosine | * | ~ |
| Creatinine | Creatinine | 75.04 ± 18.99 | ~ |
| Histamine | Histamine ^#^ | 0.4 ± 0.17 | ~ |
| Kynurenine | Kynurenine | 2.88 ± 0.69 | ~ |
| Met SO | Methioninesulfoxide | 0.78 ± 0.24 | ~ |
| Nitro-Tyr | Nitrotyrosine | * | ~ |
| OH-Pro | Hydroxyproline | * | ~ |
| PEA | Phenylethylamine | * | ~ |
| Putrescine | Putrescine ^#^ | 0.15 ± 0.05 | ~ |
| Sarcosine | Sarcosine | * | ~ |
| Serotonin | Serotonin ^#^ | 0.7 ± 0.34 | ~ |
| Spermidine | Spermidine | 0.27 ± 0.07 | ~ |
| Spermine | Spermine | * | ~ |
| Taurine | Taurine | 93.66 ± 23.95 | ~ |
| PC aa C24:0 | Phosphatidylcholine diacyl C24:0 ^#^ | 0.09 ± 0.04 | 0.15 ± 0.08 |
| PC aa C26:0 | Phosphatidylcholine diacyl C26:0 | 0.74 ± 0.24 | 1.06 ± 0.49 |
| PC aa C28:1 | Phosphatidylcholine diacyl C28:1 | 3.62 ± 0.84 | 3.43 ± 0.79 |
| PC aa C30:0 | Phosphatidylcholine diacyl C30:0 | 5.95 ± 1.67 | 4.83 ± 1.54 |
| PC aa C30:2 | Phosphatidylcholine diacyl C30:2 | * | * |
| PC aa C32:0 | Phosphatidylcholine diacyl C32:0 | 14.98 ± 2.78 | 15.39 ± 3.43 |
| PC aa C32:1 | Phosphatidylcholine diacyl C32:1 | 21.17 ± 10.62 | 21.95 ± 11.38 |
| PC aa C32:2 | Phosphatidylcholine diacyl C32:2 | 4.42 ± 1.63 | 4.05 ± 1.72 |
| PC aa C32:3 | Phosphatidylcholine diacyl C32:3 | 0.55 ± 0.14 | 0.49 ± 0.12 |
| PC aa C34:1 | Phosphatidylcholine diacyl C34:1 | 223.91 ± 46.33 | 243.79 ± 61.88 |
| PC aa C34:2 | Phosphatidylcholine diacyl C34:2 | 367.86 ± 48.54 | 400.11 ± 93.65 |
| PC aa C34:3 | Phosphatidylcholine diacyl C34:3 | 18.5 ± 5.14 | 18.42 ± 5.45 |
| PC aa C34:4 | Phosphatidylcholine diacyl C34:4 | 2.21 ± 0.77 | 2.29 ± 0.82 |
| PC aa C36:0 | Phosphatidylcholine diacyl C36:0 | 3 ± 0.77 | 2.76 ± 0.86 |
| PC aa C36:1 | Phosphatidylcholine diacyl C36:1 | 54.06 ± 13.95 | 54.48 ± 13.27 |
| PC aa C36:2 | Phosphatidylcholine diacyl C36:2 | 256.73 ± 42.39 | 235.95 ± 42.37 |
| PC aa C36:3 | Phosphatidylcholine diacyl C36:3 | 153.12 ± 29.82 | 151.14 ± 32.7 |
| PC aa C36:4 | Phosphatidylcholine diacyl C36:4 | 208.91 ± 44.39 | 219.15 ± 54.41 |
| PC aa C36:5 | Phosphatidylcholine diacyl C36:5 | 30.8 ± 14.81 | 29.88 ± 14.32 |
| PC aa C36:6 | Phosphatidylcholine diacyl C36:6 | 1.13 ± 0.43 | 1.15 ± 0.46 |
| PC aa C38:0 | Phosphatidylcholine diacyl C38:0 | 3.37 ± 0.87 | 3.3 ± 0.89 |
| PC aa C38:1 | Phosphatidylcholine diacyl C38:1 | 1.38 ± 0.41 | * |
| PC aa C38:3 | Phosphatidylcholine diacyl C38:3 | 57.77 ± 14.01 | 53.25 ± 12.82 |
| PC aa C38:4 | Phosphatidylcholine diacyl C38:4 | 117.93 ± 29.14 | 117.39 ± 28.78 |
| PC aa C38:5 | Phosphatidylcholine diacyl C38:5 | 63.1 ± 14.94 | 63.04 ± 15.64 |
| PC aa C38:6 | Phosphatidylcholine diacyl C38:6 | 90.3 ± 26.3 | 91.23 ± 27.32 |
| PC aa C40:1 | Phosphatidylcholine diacyl C40:1 | 0.42 ± 0.09 | 0.47 ± 0.1 |
| PC aa C40:2 | Phosphatidylcholine diacyl C40:2 | 0.37 ± 0.1 | 0.36 ± 0.1 |
| PC aa C40:3 | Phosphatidylcholine diacyl C40:3 | 0.69 ± 0.15 | 0.66 ± 0.15 |
| PC aa C40:4 | Phosphatidylcholine diacyl C40:4 | 4.16 ± 1.2 | 4.13 ± 1.16 |
| PC aa C40:5 | Phosphatidylcholine diacyl C40:5 | 12.79 ± 3.53 | 11.52 ± 3.14 |
| PC aa C40:6 | Phosphatidylcholine diacyl C40:6 | 32.3 ± 10 | 28.37 ± 9.35 |
| PC aa C42:0 | Phosphatidylcholine diacyl C42:0 | 0.56 ± 0.16 | 0.61 ± 0.17 |
| PC aa C42:1 | Phosphatidylcholine diacyl C42:1 | 0.27 ± 0.07 | 0.3 ± 0.08 |
| PC aa C42:2 | Phosphatidylcholine diacyl C42:2 | 0.2 ± 0.05 | 0.22 ± 0.06 |
| PC aa C42:4 | Phosphatidylcholine diacyl C42:4 | 0.21 ± 0.04 | 0.22 ± 0.05 |
| PC aa C42:5 | Phosphatidylcholine diacyl C42:5 | 0.44 ± 0.13 | 0.43 ± 0.13 |
| PC aa C42:6 | Phosphatidylcholine diacyl C42:6 | 0.6 ± 0.13 | 0.63 ± 0.14 |
| PC ae C30:0 | Phosphatidylcholine acyl-akyl C30:0 | 0.46 ± 0.13 | 0.48 ± 0.14 |
| PC ae C30:1 | Phosphatidylcholine acyl-akyl C30:1 | * | * |
| PC ae C30:2 | Phosphatidylcholine acyl-akyl C30:2 | 0.13 ± 0.04 | 0.16 ± 0.04 |
| PC ae C32:1 | Phosphatidylcholine acyl-akyl C32:1 | 2.83 ± 0.55 | 2.95 ± 0.62 |
| PC ae C32:2 | Phosphatidylcholine acyl-akyl C32:2 | 0.72 ± 0.16 | 0.77 ± 0.17 |
| PC ae C34:0 | Phosphatidylcholine acyl-akyl C34:0 | 1.69 ± 0.43 | 1.77 ± 0.46 |
| PC ae C34:1 | Phosphatidylcholine acyl-akyl C34:1 | 10.58 ± 2.21 | 10.83 ± 2.39 |
| PC ae C34:2 | Phosphatidylcholine acyl-akyl C34:2 | 12.19 ± 2.95 | 13.16 ± 3.39 |
| PC ae C34:3 | Phosphatidylcholine acyl-akyl C34:3 | 7.66 ± 2.14 | 8.74 ± 2.45 |
| PC ae C36:0 | Phosphatidylcholine acyl-akyl C36:0 | 0.93 ± 0.25 | 1.08 ± 0.34 |
| PC ae C36:1 | Phosphatidylcholine acyl-akyl C36:1 | 8.91 ± 2.08 | 8.51 ± 1.96 |
| PC ae C36:2 | Phosphatidylcholine acyl-akyl C36:2 | 15.33 ± 3.88 | 15.72 ± 4 |
| PC ae C36:3 | Phosphatidylcholine acyl-akyl C36:3 | 8.04 ± 1.85 | 8.83 ± 2.1 |
| PC ae C36:4 | Phosphatidylcholine acyl-akyl C36:4 | 20.11 ± 4.74 | 21.07 ± 5.53 |
| PC ae C36:5 | Phosphatidylcholine acyl-akyl C36:5 | 13.02 ± 3.2 | 14.02 ± 3.6 |
| PC ae C38:0 | Phosphatidylcholine acyl-akyl C38:0 | 2.24 ± 0.67 | 2.55 ± 0.78 |
| PC ae C38:1 | Phosphatidylcholine acyl-akyl C38:1 | 0.63 ± 0.26 | 0.83 ± 0.29 |
| PC ae C38:2 | Phosphatidylcholine acyl-akyl C38:2 | 2.14 ± 0.48 | 2.2 ± 0.51 |
| PC ae C38:3 | Phosphatidylcholine acyl-akyl C38:3 | 4.29 ± 0.99 | 4.35 ± 0.94 |
| PC ae C38:4 | Phosphatidylcholine acyl-akyl C38:4 | 15.49 ± 3.16 | 15.88 ± 3.38 |
| PC ae C38:5 | Phosphatidylcholine acyl-akyl C38:5 | 19.36 ± 3.83 | 20.17 ± 4.55 |
| PC ae C38:6 | Phosphatidylcholine acyl-akyl C38:6 | 8.77 ± 2.11 | 8.82 ± 2.21 |
| PC ae C40:0 | Phosphatidylcholine acyl-akyl C40:0 ^#^ | ~ | 10.31 ± 1.66 |
| PC ae C40:1 | Phosphatidylcholine acyl-akyl C40:1 | 1.6 ± 0.37 | 1.72 ± 0.4 |
| PC ae C40:2 | Phosphatidylcholine acyl-akyl C40:2 | 2.14 ± 0.49 | 2.12 ± 0.48 |
| PC ae C40:3 | Phosphatidylcholine acyl-akyl C40:3 | 1.2 ± 0.24 | 1.16 ± 0.23 |
| PC ae C40:4 | Phosphatidylcholine acyl-akyl C40:4 | 2.71 ± 0.51 | 2.62 ± 0.48 |
| PC ae C40:5 | Phosphatidylcholine acyl-akyl C40:5 | 3.71 ± 0.7 | 3.62 ± 0.65 |
| PC ae C40:6 | Phosphatidylcholine acyl-akyl C40:6 | 5.48 ± 1.35 | 5.16 ± 1.31 |
| PC ae C42:0 | Phosphatidylcholine acyl-akyl C42:0 | 0.52 ± 0.1 | 0.51 ± 0.12 |
| PC ae C42:1 | Phosphatidylcholine acyl-akyl C42:1 | 0.38 ± 0.09 | 0.38 ± 0.09 |
| PC ae C42:2 | Phosphatidylcholine acyl-akyl C42:2 | 0.64 ± 0.14 | 0.69 ± 0.15 |
| PC ae C42:3 | Phosphatidylcholine acyl-akyl C42:3 | 0.85 ± 0.19 | 0.9 ± 0.2 |
| PC ae C42:4 | Phosphatidylcholine acyl-akyl C42:4 | 0.96 ± 0.22 | 1.04 ± 0.24 |
| PC ae C42:5 | Phosphatidylcholine acyl-akyl C42:5 | 2.21 ± 0.46 | 2.4 ± 0.49 |
| PC ae C44:3 | Phosphatidylcholine acyl-akyl C44:3 | 0.13 ± 0.04 | 0.11 ± 0.03 |
| PC ae C44:4 | Phosphatidylcholine acyl-akyl C44:4 | 0.38 ± 0.1 | 0.44 ± 0.11 |
| PC ae C44:5 | Phosphatidylcholine acyl-akyl C44:5 | 1.75 ± 0.47 | 2.16 ± 0.54 |
| PC ae C44:6 | Phosphatidylcholine acyl-akyl C44:6 | 1.26 ± 0.34 | 1.4 ± 0.37 |
| lysoPC a C14:0 | lysoPhosphatidylcholine acyl C14:0 ^#^ | 6.29 ± 0.79 | 3.23 ± 0.86 |
| lysoPC a C16:0 | lysoPhosphatidylcholine acyl C16:0 | 122.98 ± 25.91 | 96.65 ± 20.18 |
| lysoPC a C16:1 | lysoPhosphatidylcholine acyl C16:1 | 3.72 ± 1.17 | 2.97 ± 1.04 |
| lysoPC a C17:0 | lysoPhosphatidylcholine acyl C17:0 | 2.1 ± 0.68 | 1.81 ± 0.52 |
| lysoPC a C18:0 | lysoPhosphatidylcholine acyl C18:0 | 32.73 ± 8.09 | 26.91 ± 6.16 |
| lysoPC a C18:1 | lysoPhosphatidylcholine acyl C18:1 | 21.61 ± 6.1 | 20.26 ± 5.87 |
| lysoPC a C18:2 | lysoPhosphatidylcholine acyl C18:2 | 28.46 ± 9.04 | 29.29 ± 9.85 |
| lysoPC a C6:0 | lysoPhosphatidylcholine acyl C6:0 | ~ | * |
| lysoPC a C20:3 | lysoPhosphatidylcholine acyl C20:3 | 2.28 ± 0.66 | 2.46 ± 0.73 |
| lysoPC a C20:4 | lysoPhosphatidylcholine acyl C20:4 | 6.14 ± 1.83 | 6.94 ± 2.19 |
| lysoPC a C24:0 | lysoPhosphatidylcholine acyl C24:0 | 0.2 ± 0.06 | 0.37 ± 0.11 |
| lysoPC a C26:0 | lysoPhosphatidylcholine acyl C26:0 ^#^ | 0.3 ± 0.14 | * |
| lysoPC a C26:1 | lysoPhosphatidylcholine acyl C26:1 ^#^ | 1.67 ± 0.17 | 2.02 ± 0.25 |
| lysoPC a C28:0 | lysoPhosphatidylcholine acyl C28:0 ^#^ | 0.33 ± 0.11 | 0.5 ± 0.21 |
| lysoPC a C28:1 | lysoPhosphatidylcholine acyl C28:1 | 0.47 ± 0.15 | 0.63 ± 0.22 |
| SM C16:0 | Sphingomyeline C16:0 | 151.19 ± 23.84 | 108.15 ± 21.38 |
| SM C16:1 | Sphingomyeline C16:1 | 23.93 ± 4.67 | 16.07 ± 3.61 |
| SM C18:0 | Sphingomyeline C18:0 | 33.23 ± 6.89 | 23.23 ± 5 |
| SM C18:1 | Sphingomyeline C18:1 | 16.75 ± 4.16 | 11.25 ± 2.98 |
| SM C20:2 | Sphingomyeline C20:2 | 0.67 ± 0.23 | 0.38 ± 0.12 |
| SM C22:3 | Sphingomyeline C22:3 | * | * |
| SM C24:0 | Sphingomyeline C24:0 | 30.53 ± 5.71 | 22.14 ± 5.13 |
| SM C24:1 | Sphingomyeline C24:1 | 76.96 ± 14.39 | 53.14 ± 12.02 |
| SM C26:0 | Sphingomyeline C26:0 ^#^ | * | 0.18 ± 0.05 |
| SM C26:1 | Sphingomyeline C26:1 | 0.64 ± 0.19 | 0.42 ± 0.13 |
| SM (OH) C14:1 | Hydroxysphingomyeline C14:1 | 9.54 ± 2.56 | 6.38 ± 1.86 |
| SM (OH) C16:1 | Hydroxysphingomyeline C16:1 | 5.19 ± 1.37 | 3.42 ± 0.89 |
| SM (OH) C22:1 | Hydroxysphingomyeline C22:1 | 20.42 ± 4.61 | 13.83 ± 3.5 |
| SM (OH) C22:2 | Hydroxysphingomyeline C22:2 | 16.56 ± 4.17 | 11.74 ± 3.14 |
| SM (OH) C24:1 | Hydroxysphingomyeline C24:1 | 2 ± 0.49 | 1.37 ± 0.36 |
| H1 | Hexose | 5300.07 ± 891.29 | 5005.34 ± 670.72 |

Metabolites excluded from the analyses are marked with * and their mean and SD are not given. Metabolites marked with ~ were not part of the other kit. ^#^ results with these metabolites require careful interpretation since the number of samples below detection and/or the coefficient of variation across all plates is relatively high
