# Supplementary material for: Body Fat Free Mass Is Associated with the Serum Metabolite Profile in a Population-Based Study
Source: PLoS One. 2012 Jun 27;7(6):e40009. doi: 10.1371/journal.pone.0040009 (PMC3384624; doi:10.1371/journal.pone.0040009)
Supplement: Table S2 — Metabolic traits significantly associated with fat free mass index in linear regression models adjusted for age, and sex (α = 5%, p-gain>170) in the KORA S4 sample. (DOC) [file pone.0040009.s003.doc]

**Table S2:** Metabolic traits significantly associated with FFMI^a^ in linear regression models adjusted for age, and sex (α = 5%, p-gain > 170) in the KORA S4 sample.

| Trait | Mean (µmol/l) | SD | Dir.^b^ | Beta | P-value | adj. P-value^c^ | R^2^ adj.^d^ | P-gain^e^ |
| --- | --- | --- | --- | --- | --- | --- | --- | --- |
| Val | 227.26 | 53.13 | pos. | 0.15 | 2.97x10^-18^ | 4.75x10^-16^ | 0.16 |  |
| Glu | 80.13 | 32.04 | pos. | 0.15 | 7.60x10^-18^ | 1.22x10^-15^ | 0.11 |  |
| Ile | 72.14 | 20.22 | pos. | 0.12 | 1.23x10^-13^ | 1.96x10^-11^ | 0.22 |  |
| Tyr | 72.09 | 20.06 | pos. | 0.13 | 2.98x10^-12^ | 4.77x10^-10^ | 0.06 |  |
| Leu | 160.51 | 44.33 | pos. | 0.11 | 1.61x10^-10^ | 2.57x10^-08^ | 0.18 |  |
| Phe | 76.73 | 17.19 | pos. | 0.11 | 1.58x10^-09^ | 2.53x10^-07^ | 0.05 |  |
| Ala | 417.99 | 101.4 | pos. | 0.10 | 5.59x10^-08^ | 8.95x10^-06^ | 0.03 |  |
| Trp | 60.17 | 12.43 | pos. | 0.07 | 4.85x10^-05^ | 7.77x10^-03^ | 0.07 |  |
| Pro | 193.96 | 58.92 | pos. | 0.08 | 1.23x10^-05^ | 1.97x10^-03^ | 0.14 |  |
| Met | 23.86 | 5.21 | pos. | 0.07 | 1.17x10^-04^ | 1.86x10^-02^ | 0.08 |  |
| Lys | 166.44 | 36.59 | pos. | 0.07 | 1.18x10^-04^ | 1.88x10^-02^ | 0.01 |  |
| ΣBCAAs | 459.91 | 113.06 | pos. | 0.13 | 1.30x10^-15^ | 2.07x10^-13^ | 0.19 |  |
| Σaromatic AAs | 208.98 | 43.75 | pos. | 0.12 | 2.11x10^-11^ | 3.37x10^-09^ | 0.07 |  |
| Gln/Val | 2.64 | 0.60 | neg. | -0.19 | 4.48x10^-28^ | 7.17x10^-26^ | 0.18 | 6.63x10^+09^ |
| Asn/Val | 0.21 | 0.05 | neg. | -0.18 | 1.12x10^-26^ | 1.80x10^-24^ | 0.17 | 2.65x10^+08^ |
| His/Val | 0.38 | 0.07 | neg. | -0.17 | 1.22x10^-23^ | 1.95x10^-21^ | 0.21 | 2.43x10^+05^ |
| Glu/Gly | 0.33 | 0.17 | pos. | 0.16 | 4.77x10^-22^ | 7.64x10^-20^ | 0.19 | 1.59x10^+04^ |
| Asn/Ile | 0.68 | 0.19 | neg. | -0.16 | 5.17x10^-22^ | 8.27x10^-20^ | 0.23 | 2.37x10^+08^ |
| Asn/Glu | 0.67 | 0.29 | neg. | -0.17 | 1.17x10^-21^ | 1.87x10^-19^ | 0.11 | 6.49x10^+03^ |
| Gln/Tyr | 8.47 | 2.16 | neg. | -0.17 | 1.83x10^-21^ | 2.93x10^-19^ | 0.10 | 1.63x10^+09^ |
| Asn/Tyr | 0.68 | 0.18 | neg. | -0.17 | 6.16x10^-21^ | 9.85x10^-19^ | 0.10 | 4.85x10^+08^ |
| Gln/Ile | 8.53 | 2.37 | neg. | -0.15 | 1.20x10^-20^ | 1.92x10^-18^ | 0.23 | 1.02x10^+07^ |
| Asn/Leu | 0.30 | 0.08 | neg. | -0.15 | 1.75x10^-18^ | 2.81x10^-16^ | 0.19 | 9.15x10^+07^ |
| Gly/Tyr | 3.89 | 1.38 | neg. | -0.15 | 7.44x10^-18^ | 1.19x10^-15^ | 0.17 | 4.01x10^+05^ |
| Gly/Phe | 3.55 | 1.08 | neg. | -0.14 | 1.03x10^-17^ | 1.65x10^-15^ | 0.19 | 1.53x10^+08^ |
| Gln/Leu | 3.81 | 1.02 | neg. | -0.14 | 3.20x10^-17^ | 5.12x10^-15^ | 0.19 | 5.02x10^+06^ |
| Asn/Phe | 0.62 | 0.14 | neg. | -0.15 | 4.14x10^-17^ | 6.62x10^-15^ | 0.08 | 3.82x10^+07^ |
| Gly/Ile | 3.96 | 1.64 | neg. | -0.13 | 1.00x10^-16^ | 1.61x10^-14^ | 0.30 | 1.22x10^+03^ |
| His/Tyr | 1.20 | 0.27 | neg. | -0.14 | 5.94x10^-16^ | 9.51x10^-14^ | 0.09 | 5.02x10^+03^ |
| Ala/Asn | 9.20 | 2.38 | pos. | 0.15 | 8.82x10^-16^ | 1.41x10^-13^ | 0.07 | 6.34x10^+07^ |
| Gly/Leu | 1.76 | 0.69 | neg. | -0.12 | 9.16x10^-15^ | 1.46x10^-12^ | 0.29 | 1.75x10^+04^ |
| Gln/Phe | 7.83 | 1.85 | neg. | -0.14 | 3.55x10^-14^ | 5.69x10^-12^ | 0.06 | 4.44x10^+04^ |
| Asn/Met | 1.98 | 0.37 | neg. | -0.13 | 5.83x10^-13^ | 9.32x10^-11^ | 0.11 | 2.00x10^+08^ |
| Ala/Gly | 1.68 | 0.57 | pos. | 0.12 | 7.14x10^-12^ | 1.14x10^-09^ | 0.11 | 7.83x10^+03^ |
| Gln/Lys | 3.57 | 0.71 | neg. | -0.12 | 7.34x10^-12^ | 1.17x10^-09^ | 0.05 | 1.60x10^+07^ |
| Asn/Lys | 0.29 | 0.06 | neg. | -0.12 | 7.36x10^-11^ | 1.18x10^-08^ | 0.04 | 1.60x10^+06^ |
| Ala/Gln | 0.74 | 0.20 | pos. | 0.12 | 9.21x10^-11^ | 1.47x10^-08^ | 0.04 | 6.07x10^+02^ |
| Gly/Trp | 4.53 | 1.43 | neg. | -0.11 | 1.91x10^-10^ | 3.06x10^-08^ | 0.20 | 2.54x10^+05^ |
| Gln/Met | 24.99 | 5.09 | neg. | -0.11 | 2.70x10^-10^ | 4.31x10^-08^ | 0.09 | 4.32x10^+05^ |
| Gln/Pro | 3.19 | 0.87 | neg. | -0.11 | 3.22x10^-10^ | 5.16x10^-08^ | 0.13 | 3.82x10^+04^ |
| Asn/Trp | 0.79 | 0.17 | neg. | -0.11 | 3.76x10^-10^ | 6.02x10^-08^ | 0.06 | 1.29x10^+05^ |
| Gly/Lys | 1.64 | 0.50 | neg. | -0.11 | 5.33x10^-10^ | 8.52x10^-08^ | 0.10 | 2.21x10^+05^ |
| Gly/Pro | 1.47 | 0.59 | neg. | -0.10 | 7.17x10^-10^ | 1.15x10^-07^ | 0.24 | 1.72x10^+04^ |
| Gly/Met | 11.46 | 3.64 | neg. | -0.10 | 7.94x10^-10^ | 1.27x10^-07^ | 0.22 | 1.47x10^+05^ |
| Asn/Pro | 0.25 | 0.07 | neg. | -0.11 | 8.12x10^-10^ | 1.30x10^-07^ | 0.13 | 1.52x10^+04^ |
| Gln/Trp | 9.91 | 2.26 | neg. | -0.11 | 1.32x10^-09^ | 2.12x10^-07^ | 0.07 | 3.67x10^+04^ |
| Asp/Gly | 0.12 | 0.04 | pos. | 0.10 | 7.07x10^-09^ | 1.13x10^-06^ | 0.06 | 6.18x10^+04^ |
| Asn/Asp | 1.72 | 0.67 | neg. | -0.09 | 1.66x10^-06^ | 2.66x10^-04^ | 0.03 | 9.48x10^+02^ |
| Gln/Orn | 10.22 | 2.28 | neg. | -0.08 | 2.22x10^-05^ | 3.55x10^-03^ | 0.02 | 6.32x10^+02^ |
| Gln/His | 7.07 | 1.08 | neg. | -0.08 | 3.01x10^-05^ | 4.82x10^-03^ | 0.03 | 4.66x10^+02^ |
| Arg/Gln | 0.22 | 0.04 | pos. | 0.07 | 5.45x10^-05^ | 8.72x10^-03^ | 0.02 | 2.58x10^+02^ |
| Asn/Orn | 0.82 | 0.20 | neg. | -0.07 | 8.86x10^-05^ | 1.42x10^-02^ | 0.03 | 4.40x10^+02^ |
| Arg/Asn | 2.80 | 0.64 | pos. | 0.07 | 1.37x10^-04^ | 2.19x10^-02^ | 0.02 | 2.85x10^+02^ |
| ΣBCAAs/ Σglucogenic AAs | 0.58 | 0.14 | pos. | 0.10 | 9.34x10^-11^ | 1.49x10^-08^ | 0.26 |  |
| C5 | 0.16 | 0.06 | pos. | 0.09 | 2.18x10^-07^ | 3.49x10^-05^ | 0.17 |  |
| C3 | 0.47 | 0.15 | pos. | 0.09 | 3.03x10^-07^ | 4.85x10^-05^ | 0.13 |  |
| C0 | 40.52 | 8.49 | pos. | 0.07 | 7.45x10^-05^ | 1.19x10^-02^ | 0.13 |  |
| C18 | 0.06 | 0.01 | neg. | -0.06 | 2.47x10^-04^ | 3.96x10^-02^ | 0.10 |  |
| C18/C5 | 0.37 | 0.14 | neg. | -0.12 | 9.00x10^-12^ | 1.44x10^-09^ | 0.08 | 2.42x10^+04^ |
| C18/C3 | 0.13 | 0.05 | neg. | -0.12 | 2.28x10^-11^ | 3.65x10^-09^ | 0.05 | 1.33x10^+04^ |
| C0/C18 | 769.01 | 221.78 | pos. | 0.11 | 3.40x10^-09^ | 5.44x10^-07^ | 0.05 | 2.19x10^+04^ |
| C18/C5:1 | 1.22 | 0.43 | neg. | -0.09 | 1.31x10^-06^ | 2.10x10^-04^ | 0.05 | 1.89x10^+02^ |
| Single PC |  |  |  |  |  |  |  |  |
| PC aa C42:0 | 0.56 | 0.16 | neg. | -0.12 | 2.43x10^-11^ | 3.89x10^-09^ | 0.06 |  |
| PC aa C42:1 | 0.27 | 0.07 | neg. | -0.11 | 4.40x10^-10^ | 7.04x10^-08^ | 0.05 |  |
| PC aa C42:2 | 0.20 | 0.05 | neg. | -0.11 | 1.82x10^-09^ | 2.91x10^-07^ | 0.03 |  |
| PC aa C38:3 | 57.77 | 14.01 | pos. | 0.10 | 4.39x10^-08^ | 7.02x10^-06^ | 0.06 |  |
| PC aa C40:1 | 0.42 | 0.09 | neg. | -0.08 | 1.55x10^-05^ | 2.47x10^-03^ | 0.02 |  |
| PC aa C40:2 | 0.37 | 0.10 | neg. | -0.08 | 1.57x10^-05^ | 2.52x10^-03^ | 0.02 |  |
| PC ae C42:3 | 0.85 | 0.19 | neg. | -0.16 | 3.19x10^-20^ | 5.10x10^-18^ | 0.10 |  |
| PC ae C36:2 | 15.33 | 3.88 | neg. | -0.14 | 1.51x10^-17^ | 2.42x10^-15^ | 0.19 |  |
| PC ae C42:4 | 0.96 | 0.22 | neg. | -0.12 | 5.26x10^-12^ | 8.42x10^-10^ | 0.06 |  |
| PC ae C34:3 | 7.66 | 2.14 | neg. | -0.12 | 6.28x10^-12^ | 1.00x10^-09^ | 0.12 |  |
| PC ae C40:6 | 5.48 | 1.35 | neg. | -0.12 | 2.60x10^-11^ | 4.16x10^-09^ | 0.09 |  |
| PC ae C44:6 | 1.26 | 0.34 | neg. | -0.12 | 3.18x10^-11^ | 5.09x10^-09^ | 0.05 |  |
| PC ae C38:2 | 2.14 | 0.48 | neg. | -0.11 | 8.21x10^-11^ | 1.31x10^-08^ | 0.10 |  |
| PC ae C40:5 | 3.71 | 0.70 | neg. | -0.11 | 5.67x10^-10^ | 9.08x10^-08^ | 0.06 |  |
| PC ae C42:5 | 2.21 | 0.46 | neg. | -0.11 | 1.09x10^-09^ | 1.75x10^-07^ | 0.05 |  |
| PC ae C42:2 | 0.64 | 0.14 | neg. | -0.11 | 1.86x10^-09^ | 2.97x10^-07^ | 0.06 |  |
| PC ae C32:1 | 2.83 | 0.55 | neg. | -0.10 | 3.65x10^-09^ | 5.84x10^-07^ | 0.09 |  |
| PC ae C44:4 | 0.38 | 0.10 | neg. | -0.11 | 4.23x10^-09^ | 6.76x10^-07^ | 0.04 |  |
| PC ae C36:1 | 8.91 | 2.08 | neg. | -0.10 | 1.19x10^-08^ | 1.91x10^-06^ | 0.15 |  |
| PC ae C44:5 | 1.75 | 0.47 | neg. | -0.10 | 1.22x10^-08^ | 1.96x10^-06^ | 0.03 |  |
| PC ae C40:3 | 1.20 | 0.24 | neg. | -0.10 | 1.46x10^-08^ | 2.33x10^-06^ | 0.17 |  |
| PC ae C32:2 | 0.72 | 0.16 | neg. | -0.09 | 1.81x10^-08^ | 2.90x10^-06^ | 0.19 |  |
| PC ae C34:2 | 12.19 | 2.95 | neg. | -0.10 | 2.29x10^-08^ | 3.67x10^-06^ | 0.14 |  |
| PC ae C34:1 | 10.58 | 2.21 | neg. | -0.09 | 8.47x10^-08^ | 1.35x10^-05^ | 0.14 |  |
| PC ae C34:0 | 1.69 | 0.43 | neg. | -0.09 | 1.28x10^-07^ | 2.04x10^-05^ | 0.08 |  |
| PC ae C44:3 | 0.13 | 0.04 | neg. | -0.10 | 2.03x10^-07^ | 3.24x10^-05^ | 0.03 |  |
| PC ae C40:1 | 1.60 | 0.37 | neg. | -0.09 | 3.80x10^-07^ | 6.08x10^-05^ | 0.03 |  |
| PC ae C40:4 | 2.71 | 0.51 | neg. | -0.08 | 6.01x10^-06^ | 9.62x10^-04^ | 0.05 |  |
| PC ae C40:2 | 2.14 | 0.49 | neg. | -0.08 | 7.35x10^-06^ | 1.18x10^-03^ | 0.09 |  |
| PC ae C30:0 | 0.46 | 0.13 | neg. | -0.08 | 1.50x10^-05^ | 2.40x10^-03^ | 0.09 |  |
| PC ae C38:1 | 0.63 | 0.26 | neg. | -0.08 | 2.00x10^-05^ | 3.19x10^-03^ | 0.02 |  |
| PC ae C36:3 | 8.04 | 1.85 | neg. | -0.07 | 2.08x10^-05^ | 3.33x10^-03^ | 0.10 |  |
| PC ae C38:0 | 2.24 | 0.67 | neg. | -0.07 | 1.16x10^-04^ | 1.86x10^-02^ | 0.07 |  |
| ΣPC ae | 181.45 | 30.52 | neg. | -0.09 | 8.61x10^-07^ | 1.38x10^-04^ | 0.10 |  |
| lysoPC a C18:2 | 28.46 | 9.04 | neg. | -0.15 | 5.12x10^-18^ | 8.19x10^-16^ | 0.16 |  |
| lysoPC a C17:0 | 2.10 | 0.68 | neg. | -0.13 | 2.68x10^-13^ | 4.29x10^-11^ | 0.07 |  |
| lysoPC a C18:1 | 21.61 | 6.10 | neg. | -0.13 | 1.44x10^-12^ | 2.30x10^-10^ | 0.11 |  |
| ΣlysoPC a | 229.29 | 47.21 | neg. | -0.08 | 2.72x10^-06^ | 4.36x10^-04^ | 0.07 |  |
|  |  |  |  |  |  |  |  |  |
| PC aa/PC aa |  |  |  |  |  |  |  |  |
| PC aa C38:3/PC aa C42:6 | 97.71 | 21.87 | pos. | 0.16 | 6.52x10^-20^ | 1.04x10^-17^ | 0.09 | 6.73x10^+11^ |
| PC aa C38:3/PC aa C42:2 | 297.65 | 96.51 | pos. | 0.16 | 1.07x10^-18^ | 1.72x10^-16^ | 0.10 | 1.69x10^+09^ |
| PC aa C36:2/PC aa C38:3 | 4.59 | 0.87 | neg. | -0.16 | 1.65x10^-18^ | 2.65x10^-16^ | 0.09 | 2.65x10^+10^ |
| PC aa C38:3/PC aa C42:0 | 111.66 | 41.58 | pos. | 0.16 | 6.60x10^-18^ | 1.06x10^-15^ | 0.07 | 3.68x10^+06^ |
| PC aa C38:3/PC aa C42:1 | 228.35 | 80.97 | pos. | 0.15 | 3.81x10^-17^ | 6.09x10^-15^ | 0.07 | 1.15x10^+07^ |
| PC aa C36:1/PC aa C38:3 | 0.95 | 0.19 | neg. | -0.15 | 6.36x10^-16^ | 1.02x10^-13^ | 0.08 | 6.90x10^+07^ |
| PC aa C38:3/PC aa C40:3 | 85.86 | 19.98 | pos. | 0.14 | 8.33x10^-16^ | 1.33x10^-13^ | 0.08 | 5.27x10^+07^ |
| PC aa C38:3/PC aa C42:4 | 282.32 | 70.15 | pos. | 0.14 | 3.18x10^-15^ | 5.08x10^-13^ | 0.09 | 1.38x10^+07^ |
| PC aa C32:0/PC aa C38:3 | 0.27 | 0.06 | neg. | -0.14 | 3.42x10^-15^ | 5.47x10^-13^ | 0.09 | 1.28x10^+07^ |
| PC aa C38:3/PC aa C40:2 | 164.64 | 48.82 | pos. | 0.14 | 8.28x10^-15^ | 1.32x10^-12^ | 0.07 | 5.30x10^+06^ |
| PC aa C34:2/PC aa C38:3 | 6.64 | 1.37 | neg. | -0.14 | 8.98x10^-15^ | 1.44x10^-12^ | 0.08 | 4.88x10^+06^ |
| PC aa C34:3/PC aa C38:3 | 0.33 | 0.09 | neg. | -0.14 | 1.09x10^-14^ | 1.75x10^-12^ | 0.07 | 4.01x10^+06^ |
| PC aa C36:3/PC aa C38:3 | 2.71 | 0.40 | neg. | -0.14 | 1.52x10^-14^ | 2.43x10^-12^ | 0.07 | 2.89x10^+06^ |
| PC aa C34:1/PC aa C38:3 | 3.99 | 0.83 | neg. | -0.13 | 7.06x10^-14^ | 1.13x10^-11^ | 0.09 | 6.21x10^+05^ |
| PC aa C38:4/PC aa C42:1 | 462.94 | 156.72 | pos. | 0.14 | 7.51x10^-14^ | 1.20x10^-11^ | 0.06 | 5.86x10^+03^ |
| PC aa C38:4/PC aa C42:0 | 227.74 | 87.12 | pos. | 0.14 | 9.35x10^-14^ | 1.50x10^-11^ | 0.06 | 2.60x10^+02^ |
| PC aa C38:4/PC aa C42:2 | 607.90 | 202.09 | pos. | 0.13 | 4.40x10^-13^ | 7.04x10^-11^ | 0.06 | 4.13x10^+03^ |
| PC aa C38:3/PC aa C38:5 | 0.94 | 0.21 | pos. | 0.13 | 1.16x10^-12^ | 1.86x10^-10^ | 0.07 | 3.77x10^+04^ |
| PC aa C38:3/PC aa C40:1 | 143.14 | 43.12 | pos. | 0.13 | 1.80x10^-12^ | 2.88x10^-10^ | 0.06 | 2.44x10^+04^ |
| PC aa C32:3/PC aa C38:3 | 0.01 | 0.00 | neg. | -0.12 | 5.26x10^-12^ | 8.42x10^-10^ | 0.12 | 8.34x10^+03^ |
| PC aa C38:4/PC aa C42:6 | 199.88 | 47.26 | pos. | 0.12 | 2.45x10^-11^ | 3.92x10^-09^ | 0.04 | 1.96x10^+07^ |
| PC aa C38:3/PC aa C42:5 | 138.06 | 37.74 | pos. | 0.12 | 4.17x10^-11^ | 6.68x10^-09^ | 0.05 | 1.05x10^+03^ |
| PC aa C38:4/PC aa C38:5 | 1.89 | 0.33 | pos. | 0.12 | 4.73x10^-11^ | 7.57x10^-09^ | 0.04 | 1.01x10^+07^ |
| PC aa C38:0/PC aa C38:3 | 0.06 | 0.02 | neg. | -0.12 | 4.91x10^-11^ | 7.85x10^-09^ | 0.04 | 8.94x10^+02^ |
| PC aa C38:3/PC aa C40:5 | 4.62 | 0.83 | pos. | 0.12 | 8.49x10^-11^ | 1.36x10^-08^ | 0.09 | 5.17x10^+02^ |
| PC aa C36:0/PC aa C38:3 | 0.05 | 0.02 | neg. | -0.12 | 9.96x10^-11^ | 1.59x10^-08^ | 0.05 | 4.41x10^+02^ |
| PC aa C38:4/PC aa C42:4 | 573.40 | 132.08 | pos. | 0.12 | 1.08x10^-10^ | 1.72x10^-08^ | 0.06 | 4.46x10^+06^ |
| PC aa C28:1/PC aa C38:3 | 0.07 | 0.02 | neg. | -0.12 | 1.72x10^-10^ | 2.74x10^-08^ | 0.06 | 2.56x10^+02^ |
| PC aa C38:4/PC aa C40:2 | 335.71 | 99.57 | pos. | 0.11 | 3.34x10^-10^ | 5.35x10^-08^ | 0.04 | 4.70x10^+04^ |
| PC aa C38:4/PC aa C40:1 | 291.96 | 89.08 | pos. | 0.10 | 2.26x10^-08^ | 3.62x10^-06^ | 0.03 | 6.82x10^+02^ |
| PC aa C40:4/PC aa C42:6 | 6.99 | 1.64 | pos. | 0.10 | 2.43x10^-08^ | 3.89x10^-06^ | 0.04 | 2.22x10^+04^ |
| PC aa C38:4/PC aa C40:3 | 175.95 | 44.68 | pos. | 0.10 | 4.75x10^-08^ | 7.59x10^-06^ | 0.03 | 1.01x10^+04^ |
| PC aa C32:0/PC aa C38:4 | 0.13 | 0.03 | neg. | -0.10 | 5.40x10^-08^ | 8.64x10^-06^ | 0.04 | 8.88x10^+03^ |
| PC aa C36:2/PC aa C38:4 | 2.27 | 0.50 | neg. | -0.10 | 7.44x10^-08^ | 1.19x10^-05^ | 0.03 | 6.45x10^+03^ |
| PC aa C38:0/PC aa C38:4 | 0.03 | 0.01 | neg. | -0.10 | 7.96x10^-08^ | 1.27x10^-05^ | 0.03 | 6.02x10^+03^ |
| PC aa C36:0/PC aa C38:4 | 0.03 | 0.01 | neg. | -0.10 | 1.02x10^-07^ | 1.63x10^-05^ | 0.03 | 4.71x10^+03^ |
| PC aa C34:3/PC aa C34:4 | 8.81 | 2.14 | neg. | -0.10 | 1.08x10^-07^ | 1.73x10^-05^ | 0.03 | 3.80x10^+05^ |
| PC aa C36:4/PC aa C42:6 | 355.68 | 78.78 | pos. | 0.10 | 1.76x10^-07^ | 2.81x10^-05^ | 0.03 | 3.07x10^+03^ |
| PC aa C38:4/PC aa C38:6 | 1.38 | 0.41 | pos. | 0.09 | 2.82x10^-07^ | 4.52x10^-05^ | 0.02 | 1.70x10^+03^ |
| PC aa C40:4/PC aa C42:4 | 20.11 | 4.91 | pos. | 0.09 | 5.52x10^-07^ | 8.83x10^-05^ | 0.02 | 4.26x10^+03^ |
| PC aa C34:2/PC aa C38:4 | 3.27 | 0.75 | neg. | -0.09 | 6.59x10^-07^ | 1.05x10^-04^ | 0.03 | 7.28x10^+02^ |
| PC aa C36:4/PC aa C42:4 | 1020.1 | 217.04 | pos. | 0.09 | 8.45x10^-07^ | 1.35x10^-04^ | 0.05 | 2.78x10^+03^ |
| PC aa C32:3/PC aa C38:4 | 0.00 | 0.00 | neg. | -0.08 | 1.17x10^-06^ | 1.87x10^-04^ | 0.12 | 4.09x10^+02^ |
| PC aa C32:2/PC aa C34:4 | 2.04 | 0.48 | neg. | -0.09 | 1.36x10^-06^ | 2.17x10^-04^ | 0.04 | 4.18x10^+04^ |
| PC aa C38:6/PC aa C40:6 | 2.84 | 0.40 | neg. | -0.09 | 1.40x10^-06^ | 2.25x10^-04^ | 0.05 | 9.23x10^+03^ |
| PC aa C38:4/PC aa C42:5 | 281.77 | 77.17 | pos. | 0.09 | 1.73x10^-06^ | 2.76x10^-04^ | 0.02 | 2.78x10^+02^ |
| PC aa C32:0/PC aa C36:4 | 0.07 | 0.01 | neg. | -0.09 | 1.75x10^-06^ | 2.79x10^-04^ | 0.06 | 1.02x10^+04^ |
| PC aa C34:3/PC aa C36:3 | 0.12 | 0.02 | neg. | -0.08 | 3.56x10^-06^ | 5.70x10^-04^ | 0.07 | 1.15x10^+04^ |
| PC aa C30:0/PC aa C34:4 | 2.83 | 0.72 | neg. | -0.08 | 4.20x10^-06^ | 6.72x10^-04^ | 0.05 | 1.35x10^+04^ |
| PC aa C36:4/PC aa C38:5 | 3.37 | 0.53 | pos. | 0.08 | 6.38x10^-06^ | 1.02x10^-03^ | 0.03 | 5.28x10^+03^ |
| PC aa C36:0/PC aa C36:4 | 0.01 | 0.00 | neg. | -0.08 | 1.22x10^-05^ | 1.95x10^-03^ | 0.02 | 2.35x10^+02^ |
| PC aa C34:3/PC aa C40:4 | 4.63 | 1.35 | neg. | -0.08 | 1.97x10^-05^ | 3.15x10^-03^ | 0.09 | 1.11x10^+03^ |
| PC aa C32:1/PC aa C34:3 | 1.12 | 0.38 | pos. | 0.08 | 2.24x10^-05^ | 3.59x10^-03^ | 0.03 | 1.83x10^+03^ |
| PC aa C32:0/PC aa C40:4 | 3.79 | 0.93 | neg. | -0.08 | 2.52x10^-05^ | 4.03x10^-03^ | 0.02 | 7.09x10^+02^ |
| PC aa C40:3/PC aa C40:4 | 0.17 | 0.05 | neg. | -0.08 | 2.66x10^-05^ | 4.26x10^-03^ | 0.02 | 4.62x10^+02^ |
| PC aa C32:3/PC aa C34:4 | 0.27 | 0.09 | neg. | -0.08 | 2.77x10^-05^ | 4.43x10^-03^ | 0.05 | 5.10x10^+02^ |
| PC aa C30:0/PC aa C32:1 | 0.31 | 0.09 | neg. | -0.08 | 3.55x10^-05^ | 5.68x10^-03^ | 0.02 | 2.52x10^+03^ |
| PC aa C34:4/PC aa C36:6 | 2.05 | 0.53 | pos. | 0.08 | 3.97x10^-05^ | 6.35x10^-03^ | 0.02 | 1.43x10^+03^ |
| PC aa C38:5/PC aa C40:4 | 15.69 | 3.54 | neg. | -0.07 | 4.71x10^-05^ | 7.54x10^-03^ | 0.04 | 4.65x10^+02^ |
| PC aa C36:4/PC aa C38:6 | 2.45 | 0.66 | pos. | 0.07 | 4.86x10^-05^ | 7.77x10^-03^ | 0.02 | 2.67x10^+02^ |
| PC aa C36:4/PC aa C40:3 | 312.80 | 74.28 | pos. | 0.07 | 5.46x10^-05^ | 8.74x10^-03^ | 0.02 | 2.25x10^+02^ |
| PC aa C36:2/PC aa C36:3 | 1.70 | 0.22 | neg. | -0.07 | 6.29x10^-05^ | 1.01x10^-02^ | 0.02 | 1.08x10^+03^ |
| PC aa C34:2/PC aa C36:4 | 1.81 | 0.32 | neg. | -0.07 | 6.39x10^-05^ | 1.02x10^-02^ | 0.03 | 5.27x10^+02^ |
| PC aa C34:3/PC aa C36:4 | 0.09 | 0.02 | neg. | -0.07 | 7.40x10^-05^ | 1.18x10^-02^ | 0.06 | 4.55x10^+02^ |
| PC aa C36:2/PC aa C40:4 | 65.02 | 15.19 | neg. | -0.07 | 8.91x10^-05^ | 1.43x10^-02^ | 0.02 | 2.46x10^+02^ |
| PC aa C34:2/PC aa C36:3 | 2.45 | 0.30 | neg. | -0.07 | 1.05x10^-04^ | 1.68x10^-02^ | 0.04 | 4.74x10^+02^ |
| PC aa C40:4/PC aa C42:5 | 9.87 | 2.77 | pos. | 0.07 | 1.22x10^-04^ | 1.95x10^-02^ | 0.02 | 1.80x10^+02^ |
| PC aa C34:4/PC aa C38:5 | 0.04 | 0.01 | pos. | 0.07 | 2.83x10^-04^ | 4.53x10^-02^ | 0.05 | 2.00x10^+02^ |
|  |  |  |  |  |  |  |  |  |
| PC ae/PC ae |  |  |  |  |  |  |  |  |
| PC ae C34:3/PC ae C36:5 | 0.60 | 0.14 | neg. | -0.14 | 1.21x10^-14^ | 1.94x10^-12^ | 0.12 | 5.19x10^+02^ |
| PC ae C34:2/PC ae C36:4 | 0.62 | 0.14 | neg. | -0.13 | 1.56x10^-14^ | 2.50x10^-12^ | 0.17 | 1.47x10^+06^ |
| PC ae C38:6/PC ae C40:6 | 1.63 | 0.28 | pos. | 0.13 | 7.66x10^-14^ | 1.22x10^-11^ | 0.08 | 3.40x10^+02^ |
| PC ae C36:4/PC ae C40:5 | 5.50 | 1.20 | pos. | 0.13 | 2.22x10^-13^ | 3.55x10^-11^ | 0.08 | 2.56x10^+03^ |
| PC ae C38:5/PC ae C40:5 | 5.28 | 0.81 | pos. | 0.13 | 9.13x10^-13^ | 1.46x10^-10^ | 0.08 | 6.21x10^+02^ |
| PC ae C36:3/PC ae C36:4 | 0.41 | 0.08 | neg. | -0.12 | 6.91x10^-12^ | 1.11x10^-09^ | 0.13 | 3.01x10^+06^ |
| PC ae C36:4/PC ae C40:1 | 12.99 | 3.34 | pos. | 0.11 | 1.06x10^-09^ | 1.70x10^-07^ | 0.04 | 3.57x10^+02^ |
| PC ae C36:4/PC ae C40:4 | 7.51 | 1.62 | pos. | 0.11 | 1.13x10^-09^ | 1.81x10^-07^ | 0.07 | 5.32x10^+03^ |
| PC ae C36:4/PC ae C38:4 | 1.31 | 0.21 | pos. | 0.11 | 1.99x10^-09^ | 3.19x10^-07^ | 0.10 | 3.28x10^+06^ |
| PC ae C36:4/PC ae C38:5 | 1.04 | 0.10 | pos. | 0.09 | 1.99x10^-06^ | 3.19x10^-04^ | 0.04 | 4.50x10^+04^ |
| PC ae C36:4/PC ae C42:1 | 53.76 | 13.14 | pos. | 0.08 | 7.87x10^-06^ | 1.26x10^-03^ | 0.03 | 2.90x10^+02^ |
| PC ae C36:4/PC ae C38:6 | 2.34 | 0.43 | pos. | 0.07 | 3.84x10^-05^ | 6.14x10^-03^ | 0.05 | 2.33x10^+03^ |
|  |  |  |  |  |  |  |  |  |
| lysoPC/lysoPC |  |  |  |  |  |  |  |  |
| lysoPC a C14:0/lysoPC a C18:2 | 0.24 | 0.08 | pos. | 0.17 | 6.49x10^-23^ | 1.04x10^-20^ | 0.18 | 7.88x10^+04^ |
| lysoPC a C14:0/lysoPC a C18:1 | 0.31 | 0.08 | pos. | 0.15 | 2.91x10^-18^ | 4.65x10^-16^ | 0.13 | 4.94x10^+05^ |
| lysoPC a C14:0/lysoPC a C17:0 | 3.30 | 1.14 | pos. | 0.16 | 4.72x10^-18^ | 7.55x10^-16^ | 0.09 | 5.69x10^+04^ |
| lysoPC a C16:0/lysoPC a C18:1 | 5.87 | 1.01 | pos. | 0.14 | 1.63x10^-15^ | 2.60x10^-13^ | 0.08 | 8.83x10^+02^ |
| lysoPC a C14:0/lysoPC a C16:0 | 0.05 | 0.01 | pos. | 0.08 | 4.87x10^-06^ | 7.80x10^-04^ | 0.07 | 1.95x10^+03^ |
| lysoPC a C14:0/lysoPC a C18:0 | 0.20 | 0.05 | pos. | 0.08 | 6.15x10^-06^ | 9.85x10^-04^ | 0.03 | 2.56x10^+02^ |
|  |  |  |  |  |  |  |  |  |
| PC aa/PC ae |  |  |  |  |  |  |  |  |
| PC aa C38:3/PC ae C42:3 | 70.43 | 21.88 | pos. | 0.20 | 1.95x10^-28^ | 3.12x10^-26^ | 0.12 | 1.63x10^+08^ |
| PC aa C38:3/PC ae C36:2 | 3.98 | 1.36 | pos. | 0.19 | 5.11x10^-27^ | 8.17x10^-25^ | 0.13 | 2.96x10^+09^ |
| PC aa C38:3/PC ae C38:2 | 27.89 | 7.48 | pos. | 0.19 | 1.47x10^-25^ | 2.35x10^-23^ | 0.11 | 5.58x10^+14^ |
| PC aa C38:3/PC ae C42:2 | 93.08 | 24.28 | pos. | 0.19 | 2.33x10^-25^ | 3.73x10^-23^ | 0.11 | 7.97x10^+15^ |
| PC aa C38:3/PC ae C36:1 | 6.70 | 1.85 | pos. | 0.18 | 1.55x10^-24^ | 2.48x10^-22^ | 0.12 | 7.70x10^+15^ |
| PC aa C38:3/PC ae C34:1 | 5.59 | 1.42 | pos. | 0.17 | 3.86x10^-22^ | 6.18x10^-20^ | 0.10 | 1.14x10^+14^ |
| PC aa C36:3/PC ae C36:2 | 10.47 | 2.95 | pos. | 0.17 | 1.00x10^-21^ | 1.60x10^-19^ | 0.14 | 1.51x10^+04^ |
| PC aa C38:3/PC ae C40:3 | 49.43 | 12.96 | pos. | 0.17 | 2.25x10^-21^ | 3.60x10^-19^ | 0.10 | 6.48x10^+12^ |
| PC ae C36:2/PC ae C38:3 | 3.60 | 0.57 | neg. | -0.17 | 7.48x10^-21^ | 1.20x10^-18^ | 0.08 | 2.02x10^+03^ |
| PC aa C38:3/PC ae C40:5 | 15.98 | 4.41 | pos. | 0.17 | 1.10x10^-20^ | 1.77x10^-18^ | 0.09 | 5.14x10^+10^ |
| PC aa C38:3/PC ae C40:6 | 11.11 | 3.71 | pos. | 0.17 | 5.16x10^-20^ | 8.26x10^-18^ | 0.08 | 5.04x10^+08^ |
| PC aa C38:3/PC ae C40:1 | 37.45 | 10.56 | pos. | 0.16 | 5.48x10^-20^ | 8.77x10^-18^ | 0.10 | 8.01x10^+11^ |
| PC aa C38:3/PC ae C42:4 | 63.03 | 21.22 | pos. | 0.16 | 7.50x10^-20^ | 1.20x10^-17^ | 0.08 | 7.02x10^+07^ |
| PC aa C38:3/PC ae C32:1 | 20.97 | 5.75 | pos. | 0.16 | 1.21x10^-19^ | 1.93x10^-17^ | 0.08 | 3.02x10^+10^ |
| PC aa C38:3/PC ae C34:0 | 35.71 | 10.67 | pos. | 0.16 | 1.53x10^-19^ | 2.44x10^-17^ | 0.08 | 2.87x10^+11^ |
| PC aa C38:3/PC ae C34:3 | 8.12 | 3.05 | pos. | 0.16 | 1.56x10^-19^ | 2.49x10^-17^ | 0.09 | 4.03x10^+07^ |
| PC aa C38:3/PC ae C38:3 | 13.77 | 3.21 | pos. | 0.16 | 3.61x10^-19^ | 5.77x10^-17^ | 0.12 | 1.22x10^+11^ |
| PC aa C38:4/PC ae C40:1 | 75.56 | 17.61 | pos. | 0.16 | 5.26x10^-19^ | 8.42x10^-17^ | 0.08 | 7.22x10^+11^ |
| PC aa C38:3/PC ae C44:4 | 158.91 | 53.81 | pos. | 0.16 | 8.21x10^-19^ | 1.31x10^-16^ | 0.08 | 5.15x10^+09^ |
| PC aa C38:3/PC ae C32:2 | 83.58 | 24.52 | pos. | 0.16 | 1.87x10^-18^ | 3.00x10^-16^ | 0.10 | 9.68x10^+09^ |
| PC aa C38:3/PC ae C42:1 | 153.87 | 36.76 | pos. | 0.16 | 3.50x10^-18^ | 5.60x10^-16^ | 0.10 | 1.25x10^+10^ |
| PC aa C38:3/PC ae C44:3 | 477.53 | 161.71 | pos. | 0.16 | 8.53x10^-18^ | 1.36x10^-15^ | 0.08 | 5.15x10^+09^ |
| PC aa C38:4/PC ae C40:5 | 32.36 | 8.09 | pos. | 0.15 | 1.65x10^-17^ | 2.65x10^-15^ | 0.07 | 3.43x10^+07^ |
| PC aa C38:3/PC ae C44:6 | 49.21 | 18.31 | pos. | 0.15 | 2.27x10^-17^ | 3.63x10^-15^ | 0.08 | 1.40x10^+06^ |
| PC aa C38:3/PC ae C36:3 | 7.44 | 2.10 | pos. | 0.15 | 2.27x10^-17^ | 3.64x10^-15^ | 0.08 | 1.93x10^+09^ |
| PC ae C34:3/PC ae C36:4 | 0.39 | 0.11 | neg. | -0.15 | 3.97x10^-17^ | 6.35x10^-15^ | 0.13 | 1.58x10^+05^ |
| PC aa C36:4/PC ae C40:1 | 133.83 | 26.17 | pos. | 0.15 | 6.61x10^-17^ | 1.06x10^-14^ | 0.07 | 5.75x10^+09^ |
| PC aa C38:3/PC ae C34:2 | 4.99 | 1.69 | pos. | 0.15 | 7.01x10^-17^ | 1.12x10^-14^ | 0.08 | 3.27x10^+08^ |
| PC aa C38:3/PC ae C42:5 | 27.15 | 8.60 | pos. | 0.15 | 8.34x10^-17^ | 1.33x10^-14^ | 0.07 | 1.31x10^+07^ |
| PC aa C38:3/PC ae C40:4 | 21.79 | 5.83 | pos. | 0.15 | 1.48x10^-16^ | 2.37x10^-14^ | 0.07 | 2.97x10^+08^ |
| PC aa C38:4/PC ae C40:6 | 22.55 | 7.14 | pos. | 0.15 | 2.40x10^-16^ | 3.84x10^-14^ | 0.08 | 1.08x10^+05^ |
| PC aa C38:3/PC ae C44:5 | 35.38 | 12.76 | pos. | 0.15 | 5.16x10^-16^ | 8.25x10^-14^ | 0.08 | 2.37x10^+07^ |
| PC aa C36:3/PC ae C38:2 | 73.66 | 15.81 | pos. | 0.14 | 1.41x10^-15^ | 2.25x10^-13^ | 0.08 | 5.82x10^+04^ |
| PC aa C38:4/PC ae C42:2 | 190.68 | 52.74 | pos. | 0.14 | 2.65x10^-15^ | 4.24x10^-13^ | 0.06 | 7.01x10^+05^ |
| PC aa C38:3/PC ae C38:0 | 27.33 | 8.44 | pos. | 0.14 | 3.53x10^-15^ | 5.65x10^-13^ | 0.07 | 1.24x10^+07^ |
| PC aa C38:4/PC ae C34:3 | 16.57 | 6.42 | pos. | 0.14 | 6.57x10^-15^ | 1.05x10^-12^ | 0.08 | 9.56x10^+02^ |
| PC aa C38:4/PC ae C44:6 | 99.78 | 36.38 | pos. | 0.14 | 1.29x10^-14^ | 2.06x10^-12^ | 0.06 | 2.47x10^+03^ |
| PC aa C38:3/PC ae C40:2 | 28.29 | 9.38 | pos. | 0.14 | 2.55x10^-14^ | 4.09x10^-12^ | 0.06 | 1.72x10^+06^ |
| PC aa C38:4/PC ae C38:2 | 57.49 | 18.01 | pos. | 0.14 | 2.89x10^-14^ | 4.62x10^-12^ | 0.07 | 2.84x10^+03^ |
| PC aa C38:4/PC ae C42:1 | 312.06 | 66.19 | pos. | 0.14 | 4.18x10^-14^ | 6.68x10^-12^ | 0.06 | 1.15x10^+10^ |
| PC aa C38:4/PC ae C36:1 | 13.80 | 4.35 | pos. | 0.13 | 1.34x10^-13^ | 2.15x10^-11^ | 0.09 | 8.89x10^+04^ |
| PC aa C38:4/PC ae C42:5 | 55.06 | 16.83 | pos. | 0.13 | 1.53x10^-13^ | 2.44x10^-11^ | 0.05 | 7.17x10^+03^ |
| PC aa C38:3/PC ae C42:0 | 113.47 | 30.57 | pos. | 0.13 | 3.88x10^-13^ | 6.21x10^-11^ | 0.07 | 1.13x10^+05^ |
| PC aa C38:3/PC ae C38:4 | 3.82 | 1.01 | pos. | 0.13 | 4.01x10^-13^ | 6.41x10^-11^ | 0.05 | 1.10x10^+05^ |
| PC aa C38:3/PC ae C30:0 | 135.46 | 48.03 | pos. | 0.13 | 3.22x10^-13^ | 5.16x10^-11^ | 0.06 | 1.36x10^+05^ |
| PC aa C36:3/PC ae C36:1 | 17.74 | 4.05 | pos. | 0.13 | 5.23x10^-13^ | 8.37x10^-11^ | 0.10 | 2.28x10^+04^ |
| PC aa C38:4/PC ae C40:4 | 44.19 | 10.85 | pos. | 0.13 | 5.51x10^-13^ | 8.81x10^-11^ | 0.05 | 1.09x10^+07^ |
| PC aa C38:4/PC ae C44:5 | 71.81 | 25.07 | pos. | 0.13 | 8.75x10^-13^ | 1.40x10^-10^ | 0.05 | 1.40x10^+04^ |
| PC aa C38:3/PC ae C38:1 | 109.71 | 77.67 | pos. | 0.13 | 8.97x10^-13^ | 1.44x10^-10^ | 0.05 | 4.89x10^+04^ |
| PC aa C36:4/PC ae C40:5 | 57.60 | 13.47 | pos. | 0.13 | 9.00x10^-13^ | 1.44x10^-10^ | 0.06 | 6.30x10^+02^ |
| PC aa C38:4/PC ae C32:2 | 170.96 | 52.95 | pos. | 0.13 | 1.34x10^-12^ | 2.14x10^-10^ | 0.10 | 1.35x10^+04^ |
| PC aa C38:4/PC ae C32:1 | 42.98 | 12.90 | pos. | 0.13 | 1.67x10^-12^ | 2.68x10^-10^ | 0.06 | 2.18x10^+03^ |
| PC aa C38:4/PC ae C44:3 | 975.91 | 336.09 | pos. | 0.13 | 1.89x10^-12^ | 3.03x10^-10^ | 0.05 | 1.07x10^+05^ |
| PC aa C36:4/PC ae C42:2 | 338.33 | 84.46 | pos. | 0.13 | 2.21x10^-12^ | 3.54x10^-10^ | 0.05 | 8.40x10^+02^ |
| PC aa C38:4/PC ae C38:4 | 7.71 | 1.67 | pos. | 0.12 | 6.82x10^-12^ | 1.09x10^-09^ | 0.05 | 7.02x10^+07^ |
| PC aa C40:4/PC ae C42:2 | 6.70 | 1.99 | pos. | 0.12 | 7.82x10^-12^ | 1.25x10^-09^ | 0.06 | 2.37x10^+02^ |
| PC aa C36:3/PC ae C42:2 | 247.25 | 55.47 | pos. | 0.12 | 8.01x10^-12^ | 1.28x10^-09^ | 0.05 | 2.32x10^+02^ |
| PC aa C38:4/PC ae C40:3 | 101.79 | 30.82 | pos. | 0.12 | 1.02x10^-11^ | 1.63x10^-09^ | 0.08 | 1.43x10^+03^ |
| PC aa C38:4/PC ae C34:1 | 11.54 | 3.54 | pos. | 0.12 | 1.25x10^-11^ | 1.99x10^-09^ | 0.07 | 6.79x10^+03^ |
| PC aa C38:4/PC ae C44:4 | 327.70 | 125.99 | pos. | 0.12 | 1.32x10^-11^ | 2.10x10^-09^ | 0.04 | 3.21x10^+02^ |
| PC aa C38:4/PC ae C34:0 | 73.64 | 25.41 | pos. | 0.12 | 1.47x10^-11^ | 2.35x10^-09^ | 0.06 | 8.67x10^+03^ |
| PC aa C36:3/PC ae C34:1 | 14.79 | 2.93 | pos. | 0.12 | 1.59x10^-11^ | 2.55x10^-09^ | 0.09 | 5.31x10^+03^ |
| PC aa C40:4/PC ae C36:1 | 0.49 | 0.17 | pos. | 0.12 | 1.64x10^-11^ | 2.63x10^-09^ | 0.12 | 7.28x10^+02^ |
| PC aa C36:4/PC ae C36:1 | 24.42 | 6.87 | pos. | 0.12 | 2.28x10^-11^ | 3.65x10^-09^ | 0.11 | 5.24x10^+02^ |
| PC aa C38:4/PC ae C34:2 | 10.24 | 3.81 | pos. | 0.12 | 6.44x10^-11^ | 1.03x10^-08^ | 0.07 | 3.56x10^+02^ |
| PC aa C36:4/PC ae C32:2 | 302.36 | 82.77 | pos. | 0.11 | 1.01x10^-10^ | 1.61x10^-08^ | 0.13 | 1.80x10^+02^ |
| PC aa C38:3/PC ae C36:0 | 64.84 | 18.88 | pos. | 0.12 | 1.33x10^-10^ | 2.12x10^-08^ | 0.06 | 3.30x10^+02^ |
| PC aa C34:4/PC ae C40:1 | 1.40 | 0.41 | pos. | 0.11 | 2.10x10^-10^ | 3.36x10^-08^ | 0.08 | 1.81x10^+03^ |
| PC aa C38:4/PC ae C38:0 | 55.75 | 17.01 | pos. | 0.11 | 2.51x10^-10^ | 4.01x10^-08^ | 0.06 | 4.65x10^+05^ |
| PC aa C36:4/PC ae C44:3 | 1727.9 | 547.87 | pos. | 0.12 | 2.58x10^-10^ | 4.13x10^-08^ | 0.04 | 7.85x10^+02^ |
| PC aa C40:4/PC ae C34:1 | 0.40 | 0.13 | pos. | 0.11 | 3.95x10^-10^ | 6.32x10^-08^ | 0.10 | 2.14x10^+02^ |
| PC aa C40:4/PC ae C40:1 | 2.68 | 0.81 | pos. | 0.11 | 4.30x10^-10^ | 6.88x10^-08^ | 0.04 | 8.84x10^+02^ |
| PC aa C36:4/PC ae C34:0 | 129.92 | 39.18 | pos. | 0.11 | 4.34x10^-10^ | 6.94x10^-08^ | 0.08 | 2.94x10^+02^ |
| PC aa C34:4/PC ae C34:0 | 1.34 | 0.44 | pos. | 0.11 | 4.61x10^-10^ | 7.37x10^-08^ | 0.05 | 2.77x10^+02^ |
| PC aa C36:3/PC ae C34:0 | 94.54 | 24.27 | pos. | 0.11 | 5.19x10^-10^ | 8.31x10^-08^ | 0.06 | 2.46x10^+02^ |
| PC aa C40:4/PC ae C34:0 | 2.58 | 0.92 | pos. | 0.11 | 5.78x10^-10^ | 9.26x10^-08^ | 0.08 | 2.21x10^+02^ |
| PC aa C36:3/PC ae C40:1 | 99.08 | 23.05 | pos. | 0.11 | 6.12x10^-10^ | 9.79x10^-08^ | 0.05 | 6.21x10^+02^ |
| PC aa C38:4/PC ae C36:3 | 15.34 | 5.11 | pos. | 0.11 | 2.21x10^-09^ | 3.54x10^-07^ | 0.05 | 9.40x10^+03^ |
| PC aa C38:4/PC ae C40:2 | 57.90 | 19.88 | pos. | 0.11 | 2.45x10^-09^ | 3.92x10^-07^ | 0.05 | 3.00x10^+03^ |
| PC aa C40:4/PC ae C42:1 | 10.94 | 2.43 | pos. | 0.11 | 2.95x10^-09^ | 4.72x10^-07^ | 0.04 | 7.75x10^+05^ |
| PC aa C36:4/PC ae C42:1 | 555.46 | 109.61 | pos. | 0.11 | 4.33x10^-09^ | 6.94x10^-07^ | 0.04 | 5.27x10^+05^ |
| PC aa C36:4/PC ae C40:4 | 78.61 | 17.85 | pos. | 0.11 | 6.18x10^-09^ | 9.89x10^-07^ | 0.05 | 9.73x10^+02^ |
| PC aa C40:4/PC ae C40:4 | 1.56 | 0.47 | pos. | 0.10 | 7.98x10^-09^ | 1.28x10^-06^ | 0.05 | 7.53x10^+02^ |
| PC aa C38:4/PC ae C38:1 | 226.20 | 162.23 | pos. | 0.10 | 1.06x10^-08^ | 1.70x10^-06^ | 0.03 | 1.88x10^+03^ |
| PC aa C36:4/PC ae C38:0 | 98.56 | 26.41 | pos. | 0.10 | 1.76x10^-08^ | 2.82x10^-06^ | 0.07 | 6.61x10^+03^ |
| PC aa C36:3/PC ae C36:3 | 19.62 | 4.36 | pos. | 0.10 | 2.17x10^-08^ | 3.47x10^-06^ | 0.05 | 9.60x10^+02^ |
| PC aa C38:4/PC ae C42:0 | 231.10 | 60.62 | pos. | 0.10 | 3.25x10^-08^ | 5.20x10^-06^ | 0.04 | 1.47x10^+04^ |
| PC aa C34:4/PC ae C38:0 | 1.02 | 0.32 | pos. | 0.10 | 6.42x10^-08^ | 1.03x10^-05^ | 0.03 | 1.81x10^+03^ |
| PC aa C38:4/PC ae C30:0 | 281.12 | 116.99 | pos. | 0.10 | 7.20x10^-08^ | 1.15x10^-05^ | 0.05 | 2.08x10^+02^ |
| PC aa C36:4/PC ae C38:4 | 13.73 | 2.81 | pos. | 0.09 | 2.19x10^-07^ | 3.50x10^-05^ | 0.05 | 2.99x10^+04^ |
| PC aa C38:4/PC ae C38:3 | 28.55 | 8.61 | pos. | 0.09 | 3.21x10^-07^ | 5.13x10^-05^ | 0.08 | 1.49x10^+03^ |
| PC aa C40:4/PC ae C38:4 | 0.27 | 0.08 | pos. | 0.09 | 1.18x10^-06^ | 1.89x10^-04^ | 0.05 | 5.55x10^+03^ |
| PC aa C38:4/PC ae C38:6 | 13.87 | 3.63 | pos. | 0.09 | 1.81x10^-06^ | 2.89x10^-04^ | 0.02 | 2.65x10^+02^ |
| PC aa C38:4/PC ae C38:5 | 6.15 | 1.27 | pos. | 0.09 | 1.88x10^-06^ | 3.01x10^-04^ | 0.03 | 2.54x10^+02^ |
| PC aa C38:0/PC ae C36:4 | 0.17 | 0.05 | neg. | -0.08 | 4.24x10^-06^ | 6.78x10^-04^ | 0.04 | 3.54x10^+02^ |
| PC aa C40:4/PC ae C38:3 | 1.00 | 0.34 | pos. | 0.08 | 8.62x10^-06^ | 1.38x10^-03^ | 0.12 | 1.48x10^+03^ |
| PC aa C36:3/PC ae C42:1 | 409.17 | 84.37 | pos. | 0.08 | 9.88x10^-06^ | 1.58x10^-03^ | 0.03 | 2.31x10^+02^ |
| PC aa C34:4/PC ae C42:1 | 5.83 | 1.84 | pos. | 0.08 | 1.10x10^-05^ | 1.75x10^-03^ | 0.07 | 2.08x10^+02^ |
| PC aa C36:3/PC ae C38:3 | 36.65 | 7.41 | pos. | 0.07 | 3.22x10^-05^ | 5.15x10^-03^ | 0.11 | 3.96x10^+02^ |
| PC aa C36:4/PC ae C36:0 | 234.40 | 62.39 | pos. | 0.07 | 1.82x10^-04^ | 2.91x10^-02^ | 0.01 | 1.78x10^+02^ |
|  |  |  |  |  |  |  |  |  |
| PC/lysoPC |  |  |  |  |  |  |  |  |
| PC aa C38:3/lysoPC a C18:2 | 2.27 | 0.99 | pos. | 0.17 | 2.10x10^-22^ | 3.37x10^-20^ | 0.19 | 2.43x10^+04^ |
| PC aa C38:3/lysoPC a C18:1 | 2.87 | 1.03 | pos. | 0.16 | 8.65x10^-22^ | 1.38x10^-19^ | 0.17 | 1.66x10^+09^ |
| PC aa C38:3/lysoPC a C17:0 | 30.75 | 14.02 | pos. | 0.16 | 1.73x10^-19^ | 2.77x10^-17^ | 0.08 | 1.55x10^+06^ |
| PC aa C38:4/lysoPC a C18:1 | 5.85 | 2.10 | pos. | 0.14 | 3.80x10^-16^ | 6.09x10^-14^ | 0.12 | 3.77x10^+03^ |
| PC aa C38:3/lysoPC a C18:0 | 1.85 | 0.56 | pos. | 0.13 | 1.56x10^-13^ | 2.49x10^-11^ | 0.10 | 2.82x10^+05^ |
| PC aa C42:2/lysoPC a C26:1 | 0.12 | 0.03 | neg. | -0.12 | 7.58x10^-12^ | 1.21x10^-09^ | 0.05 | 2.40x10^+02^ |
| PC aa C38:3/lysoPC a C16:0 | 0.49 | 0.14 | pos. | 0.12 | 1.67x10^-11^ | 2.67x10^-09^ | 0.12 | 2.63x10^+03^ |
| PC aa C38:4/lysoPC a C20:4 | 20.19 | 5.51 | pos. | 0.10 | 4.73x10^-10^ | 7.57x10^-08^ | 0.19 | 1.01x10^+06^ |
| PC aa C38:4/lysoPC a C18:0 | 3.76 | 1.14 | pos. | 0.11 | 5.19x10^-09^ | 8.30x10^-07^ | 0.06 | 9.24x10^+04^ |
| PC aa C40:1/lysoPC a C26:1 | 0.25 | 0.06 | neg. | -0.10 | 5.03x10^-08^ | 8.05x10^-06^ | 0.04 | 3.07x10^+02^ |
| PC aa C36:4/lysoPC a C20:4 | 35.84 | 9.27 | pos. | 0.09 | 3.29x10^-07^ | 5.26x10^-05^ | 0.18 | 3.68x10^+04^ |
| PC aa C38:4/lysoPC a C16:0 | 0.99 | 0.28 | pos. | 0.09 | 7.55x10^-07^ | 1.21x10^-04^ | 0.07 | 6.35x10^+02^ |
| PC aa C42:6/lysoPC a C14:0 | 0.10 | 0.02 | neg. | -0.09 | 1.04x10^-06^ | 1.66x10^-04^ | 0.03 | 5.22x10^+02^ |
| PC aa C40:4/lysoPC a C18:0 | 0.13 | 0.04 | pos. | 0.08 | 4.76x10^-06^ | 7.62x10^-04^ | 0.02 | 3.31x10^+02^ |
| PC aa C40:4/lysoPC a C20:4 | 0.72 | 0.24 | pos. | 0.07 | 3.62x10^-05^ | 5.79x10^-03^ | 0.09 | 3.35x10^+02^ |
| PC aa C36:4/lysoPC a C16:0 | 1.75 | 0.44 | pos. | 0.07 | 5.22x10^-05^ | 8.35x10^-03^ | 0.06 | 1.82x10^+02^ |
| PC ae C38:2/lysoPC a C14:0 | 0.34 | 0.08 | neg. | -0.14 | 1.64x10^-14^ | 2.62x10^-12^ | 0.11 | 5.01x10^+03^ |
| PC ae C36:1/lysoPC a C14:0 | 1.43 | 0.33 | neg. | -0.12 | 7.20x10^-13^ | 1.15x10^-10^ | 0.16 | 1.66x10^+04^ |
| PC ae C34:0/lysoPC a C14:0 | 0.27 | 0.06 | neg. | -0.13 | 8.82x10^-13^ | 1.41x10^-10^ | 0.12 | 1.45x10^+05^ |
| PC ae C42:2/lysoPC a C14:0 | 0.10 | 0.02 | neg. | -0.13 | 1.45x10^-12^ | 2.32x10^-10^ | 0.07 | 1.28x10^+03^ |
| PC ae C34:1/lysoPC a C14:0 | 1.69 | 0.35 | neg. | -0.12 | 1.08x10^-11^ | 1.73x10^-09^ | 0.15 | 7.85x10^+03^ |
| PC ae C40:1/lysoPC a C14:0 | 0.26 | 0.06 | neg. | -0.12 | 1.54x10^-10^ | 2.47x10^-08^ | 0.05 | 2.46x10^+03^ |
| PC ae C30:0/lysoPC a C14:0 | 0.07 | 0.02 | neg. | -0.11 | 6.13x10^-10^ | 9.80x10^-08^ | 0.12 | 2.44x10^+04^ |
| PC ae C42:0/lysoPC a C26:1 | 0.31 | 0.06 | neg. | -0.09 | 1.86x10^-06^ | 2.98x10^-04^ | 0.02 | 3.42x10^+02^ |
| PC ae C36:4/lysoPC a C20:4 | 3.46 | 0.97 | pos. | 0.07 | 2.73x10^-05^ | 4.37x10^-03^ | 0.12 | 4.44x10^+02^ |
| SM C16:0 | 151.19 | 23.84 | neg. | -0.07 | 2.97x10^-05^ | 4.76x10^-03^ | 0.07 |  |
| SM (OH) C14:1 | 9.54 | 2.56 | neg. | -0.07 | 9.83x10^-05^ | 1.57x10^-02^ | 0.18 |  |
| SM (OH) C16:1 | 5.19 | 1.37 | neg. | -0.06 | 1.08x10^-04^ | 1.73x10^-02^ | 0.18 |  |
| SM C16:0/SM C16:1 | 6.40 | 0.76 | neg. | -0.12 | 9.51x10^-14^ | 1.52x10^-11^ | 0.29 | 3.13x10^+08^ |
| SM (OH) C16:1/SM C18:0 | 0.16 | 0.03 | neg. | -0.12 | 2.05x10^-12^ | 3.28x10^-10^ | 0.10 | 5.27x10^+07^ |
| SM (OH) C16:1/SM C18:1 | 0.31 | 0.06 | neg. | -0.13 | 2.19x10^-12^ | 3.51x10^-10^ | 0.05 | 4.94x10^+07^ |
| SM C16:0/SM C18:0 | 4.64 | 0.71 | neg. | -0.11 | 3.20x10^-10^ | 5.11x10^-08^ | 0.09 | 9.30x10^+04^ |
| SM C16:0/SM C18:1 | 9.36 | 1.83 | neg. | -0.10 | 3.57x10^-10^ | 5.71x10^-08^ | 0.24 | 8.33x10^+04^ |
| SM (OH) C22:2/SM C18:1 | 1.00 | 0.19 | neg. | -0.11 | 2.59x10^-09^ | 4.14x10^-07^ | 0.05 | 2.93x10^+05^ |
| SM (OH) C14:1/SM C18:1 | 0.58 | 0.14 | neg. | -0.10 | 3.40x10^-08^ | 5.45x10^-06^ | 0.03 | 2.89x10^+03^ |
| SM (OH) C14:1/SM C18:0 | 0.29 | 0.06 | neg. | -0.10 | 6.07x10^-08^ | 9.71x10^-06^ | 0.06 | 1.62x10^+03^ |
| SM (OH) C22:2/SM C16:1 | 0.69 | 0.12 | neg. | -0.09 | 3.32x10^-07^ | 5.31x10^-05^ | 0.10 | 2.29x10^+03^ |
| SM (OH) C22:2/SM C18:0 | 0.50 | 0.10 | neg. | -0.09 | 3.59x10^-07^ | 5.74x10^-05^ | 0.15 | 2.12x10^+03^ |
| SM (OH) C14:1/SM C16:1 | 0.40 | 0.08 | neg. | -0.09 | 4.60x10^-07^ | 7.36x10^-05^ | 0.03 | 2.14x10^+02^ |
| SM C18:1/SM C26:1 | 27.63 | 8.81 | pos. | 0.08 | 9.87x10^-06^ | 1.58x10^-03^ | 0.14 | 3.76x10^+02^ |
| SM (OH) C24:1/SM C18:1 | 0.12 | 0.03 | neg. | -0.08 | 1.21x10^-05^ | 1.94x10^-03^ | 0.08 | 4.27x10^+02^ |
| SM (OH) C24:1/SM C18:0 | 0.06 | 0.01 | neg. | -0.08 | 1.59x10^-05^ | 2.54x10^-03^ | 0.02 | 3.26x10^+02^ |
| SM C18:0/SM C26:1 | 54.77 | 15.49 | pos. | 0.08 | 2.17x10^-05^ | 3.47x10^-03^ | 0.06 | 1.71x10^+02^ |
| SM C18:1/SM C24:1 | 0.22 | 0.05 | pos. | 0.06 | 7.06x10^-05^ | 1.13x10^-02^ | 0.23 | 4.92x10^+02^ |
| H1 | 5300.1 | 891.29 | pos. | 0.17 | 2.67x10^-21^ | 4.27x10^-19^ | 0.14 |  |
| Kynurenine | 2.88 | 0.69 | pos. | 0.13 | 1.11x10^-13^ | 1.78x10^-11^ | 0.11 |  |
| alpha AAA | 0.68 | 0.27 | pos. | 0.09 | 8.51x10^-07^ | 1.36x10^-04^ | 0.05 |  |
| Serotonin | 0.70 | 0.34 | neg. | -0.08 | 2.21x10^-05^ | 3.54x10^-03^ | 0.03 |  |
| Kynurenine/total DMA | 2.43 | 0.65 | pos. | 0.15 | 5.16x10^-18^ | 8.25x10^-16^ | 0.09 | 2.16x10^+04^ |
| Serotonin/alpha AAA | 2.85 | 29.44 | neg. | -0.11 | 1.24x10^-09^ | 1.98x10^-07^ | 0.06 | 6.89x10^+02^ |
| alpha AAA/total DMA | 0.58 | 0.26 | pos. | 0.11 | 1.59x10^-09^ | 2.54x10^-07^ | 0.06 | 5.37x10^+02^ |
| Met SO/SDMA | 1.19 | 1.78 | pos. | 0.08 | 3.47x10^-06^ | 5.55x10^-04^ | 0.02 | 1.35x10^+03^ |
| Met SO/total DMA | 0.66 | 0.23 | pos. | 0.08 | 8.03x10^-06^ | 1.28x10^-03^ | 0.02 | 5.00x10^+02^ |

^a^ Fat Free Mass Index; ^b^ direction of the association (positive or negative); ^c^ for multiple testing adjusted p-value; ^d^ adjusted R^2^ of the linear model; ^e^ p-gain, fold decrease in the P value of association for the pair of metabolites, compared to the lowest of two p values for the single metabolites; AAs amino acids; Σ aromatic amino acids is the sum of tyrosine, phenylalanine, and tryptophan; Σ BCAAs is the sum of valine, isoleucine, and leucine; Σ glucogenic amino acids is the sum of alanine, glycine, and serine.
