# Supplementary material for: Body Fat Free Mass Is Associated with the Serum Metabolite Profile in a Population-Based Study
Source: PLoS One. 2012 Jun 27;7(6):e40009. doi: 10.1371/journal.pone.0040009 (PMC3384624; doi:10.1371/journal.pone.0040009)
Supplement: Table S3 — Metabolic traits significantly associated with fat free mass index in linear regression models adjusted for age, sex and batch (α = 5%, p-gain>150) in the KORA F4 weight-stable sample. (DOC) [file pone.0040009.s004.doc]

**Table S3:** Metabolic traits significantly associated with FFMI^a^ in linear regression models adjusted for age, sex and batch (α = 5%, p-gain > 150) in the KORA F4 weight-stable sample.

| Trait | Mean (µmol/l) | SD | Dir.^b^ | Beta | P-value | adj. P-value^c^ | R^2^ adj.^d^ | P-gain^e^ |
| --- | --- | --- | --- | --- | --- | --- | --- | --- |
| xLeu | 210.42 | 43.19 | pos. | 0.13 | 4.75x10^-16^ | 6.80x10^-14^ | 0.39 |  |
| Gly | 314.88 | 83.47 | neg. | -0.13 | 6.53x10^-12^ | 9.33x10^-10^ | 0.14 |  |
| Val | 273.25 | 59.21 | pos. | 0.11 | 2.05x10^-11^ | 2.94x10^-09^ | 0.35 |  |
| Tyr | 84.60 | 17.27 | pos. | 0.10 | 1.19x10^-07^ | 1.70x10^-05^ | 0.10 |  |
| Ser | 130.82 | 24.44 | neg. | -0.09 | 1.65x10^-05^ | 2.36x10^-03^ | 0.04 |  |
| Orn | 81.50 | 18.59 | pos. | 0.07 | 1.17x10^-04^ | 1.67x10^-02^ | 0.14 |  |
| ΣBCAAs | 483.67 | 97.47 | pos. | 0.13 | 1.51x10^-14^ | 2.15x10^-12^ | 0.38 |  |
| Σaromatic AAs | 229.04 | 32.96 | pos. | 0.08 | 5.37x10^-05^ | 7.68x10^-03^ | 0.11 |  |
| Gly/xLeu | 1.57 | 0.57 | neg. | -0.17 | 6.01x10^-27^ | 8.60x10^-25^ | 0.42 | 7.90x10^+10^ |
| Gly/Val | 1.22 | 0.46 | neg. | -0.16 | 3.71x10^-22^ | 5.31x10^-20^ | 0.39 | 1.76x10^+10^ |
| Gly/Tyr | 3.87 | 1.30 | neg. | -0.17 | 5.22x10^-21^ | 7.47x10^-19^ | 0.25 | 1.25x10^+09^ |
| Ser/xLeu | 0.65 | 0.18 | neg. | -0.16 | 9.84x10^-21^ | 1.41x10^-18^ | 0.35 | 4.83x10^+04^ |
| Ser/Val | 0.50 | 0.13 | neg. | -0.15 | 2.37x10^-20^ | 3.38x10^-18^ | 0.36 | 8.68x10^+08^ |
| Gln/xLeu | 3.07 | 0.62 | neg. | -0.16 | 9.30x10^-20^ | 1.33x10^-17^ | 0.27 | 5.11x10^+03^ |
| Gly/Orn | 4.05 | 1.40 | neg. | -0.15 | 8.50x10^-19^ | 1.21x10^-16^ | 0.31 | 7.68x10^+06^ |
| Gln/Val | 2.37 | 0.45 | neg. | -0.16 | 1.88x10^-18^ | 2.69x10^-16^ | 0.21 | 1.09x10^+07^ |
| His/xLeu | 0.49 | 0.10 | neg. | -0.13 | 2.16x10^-18^ | 3.09x10^-16^ | 0.52 | 2.20x10^+02^ |
| Ser/Tyr | 1.60 | 0.40 | neg. | -0.15 | 3.97x10^-16^ | 5.68x10^-14^ | 0.19 | 2.99x10^+08^ |
| Gly/Phe | 5.28 | 1.64 | neg. | -0.15 | 2.67x10^-15^ | 3.82x10^-13^ | 0.21 | 2.44x10^+03^ |
| Trp/Val | 0.31 | 0.06 | neg. | -0.12 | 3.34x10^-14^ | 4.77x10^-12^ | 0.43 | 6.15x10^+02^ |
| Gln/Tyr | 7.64 | 1.50 | neg. | -0.14 | 1.20x10^-12^ | 1.72x10^-10^ | 0.12 | 9.88x10^+04^ |
| Met/Tyr | 0.38 | 0.06 | neg. | -0.13 | 6.70x10^-12^ | 9.58x10^-10^ | 0.21 | 1.77x10^+04^ |
| Phe/Ser | 0.48 | 0.10 | pos. | 0.13 | 1.90x10^-11^ | 2.72x10^-09^ | 0.14 | 8.65x10^+05^ |
| Orn/Ser | 0.64 | 0.17 | pos. | 0.12 | 2.12x10^-11^ | 3.03x10^-09^ | 0.22 | 7.78x10^+05^ |
| Trp/Tyr | 1.01 | 0.16 | neg. | -0.12 | 5.93x10^-10^ | 8.48x10^-08^ | 0.13 | 2.00x10^+02^ |
| Gln/Orn | 8.00 | 1.72 | neg. | -0.11 | 1.21x10^-08^ | 1.73x10^-06^ | 0.06 | 9.65x10^+03^ |
| Gln/Phe | 10.44 | 1.79 | neg. | -0.10 | 2.50x10^-07^ | 3.57x10^-05^ | 0.13 | 4.18x10^+03^ |
| ΣBCAAs/ Σglucogenic AAs | 1.14 | 0.34 | pos. | 0.17 | 2.68x10^-27^ | 3.83x10^-25^ | 0.43 |  |
| C8:1 | 0.09 | 0.04 | pos. | 0.11 | 1.39x10^-08^ | 1.98x10^-06^ | 0.07 |  |
| C9 | 0.05 | 0.02 | neg. | -0.08 | 2.37x10^-05^ | 3.40x10^-03^ | 0.06 |  |
| C3 | 0.38 | 0.12 | pos. | 0.08 | 3.41x10^-05^ | 4.88x10^-03^ | 0.14 |  |
| C5 | 0.11 | 0.04 | pos. | 0.08 | 5.85x10^-05^ | 8.36x10^-03^ | 0.17 |  |
| C0 | 35.05 | 7.36 | pos. | 0.07 | 1.17x10^-04^ | 1.68x10^-02^ | 0.13 |  |
| C8:1/C9 | 1.91 | 1.20 | pos. | 0.15 | 2.23x10^-14^ | 3.18x10^-12^ | 0.07 | 6.23x10^+05^ |
| C12/C8:1 | 1.77 | 0.83 | neg. | -0.15 | 7.98x10^-14^ | 1.14x10^-11^ | 0.06 | 1.74x10^+05^ |
| C14:2/C8:1 | 0.41 | 0.19 | neg. | -0.14 | 3.38x10^-13^ | 4.84x10^-11^ | 0.08 | 4.10x10^+04^ |
| C7-DC/C8:1 | 0.61 | 0.28 | neg. | -0.14 | 6.24x10^-13^ | 8.92x10^-11^ | 0.09 | 2.22x10^+04^ |
| C10/C8:1 | 4.79 | 2.45 | neg. | -0.14 | 1.78x10^-12^ | 2.55x10^-10^ | 0.07 | 7.78x10^+03^ |
| C14:1-OH/C8:1 | 0.20 | 0.08 | neg. | -0.14 | 3.77x10^-12^ | 5.40x10^-10^ | 0.05 | 3.68x10^+03^ |
| C12:1/C8:1 | 1.97 | 0.85 | neg. | -0.14 | 6.67x10^-12^ | 9.53x10^-10^ | 0.06 | 2.08x10^+03^ |
| C14:1/C8:1 | 2.08 | 0.92 | neg. | -0.13 | 1.63x10^-11^ | 2.33x10^-09^ | 0.12 | 8.53x10^+02^ |
| C14:2-OH/C8:1 | 0.13 | 0.06 | neg. | -0.13 | 2.93x10^-11^ | 4.19x10^-09^ | 0.14 | 4.74x10^+02^ |
| C5/C9 | 2.56 | 1.17 | pos. | 0.13 | 3.11x10^-11^ | 4.44x10^-09^ | 0.15 | 7.64x10^+05^ |
| C14/C8:1 | 0.63 | 0.28 | neg. | -0.13 | 6.95x10^-11^ | 9.93x10^-09^ | 0.06 | 2.00x10^+02^ |
| C3/C9 | 8.46 | 4.00 | pos. | 0.13 | 7.71x10^-11^ | 1.10x10^-08^ | 0.11 | 3.08x10^+05^ |
| C0/C9 | 792.55 | 331.39 | pos. | 0.12 | 3.58x10^-09^ | 5.12x10^-07^ | 0.07 | 6.64x10^+03^ |
| C10/C6 (C4:1-DC) | 5.07 | 1.20 | neg. | -0.12 | 4.11x10^-09^ | 5.88x10^-07^ | 0.05 | 1.42x10^+06^ |
| C12/C5 | 1.23 | 0.55 | neg. | -0.11 | 1.52x10^-08^ | 2.17x10^-06^ | 0.13 | 3.85x10^+03^ |
| C14:2-OH/C5 | 0.09 | 0.03 | neg. | -0.10 | 3.05x10^-08^ | 4.36x10^-06^ | 0.23 | 1.92x10^+03^ |
| C5/C7-DC | 2.85 | 1.29 | pos. | 0.11 | 3.20x10^-08^ | 4.58x10^-06^ | 0.10 | 1.83x10^+03^ |
| C12/C3 | 0.37 | 0.17 | neg. | -0.11 | 6.35x10^-08^ | 9.07x10^-06^ | 0.07 | 5.38x10^+02^ |
| C14:1-OH/C5 | 0.14 | 0.05 | neg. | -0.10 | 9.97x10^-08^ | 1.43x10^-05^ | 0.13 | 5.87x10^+02^ |
| C12/C16 | 1.12 | 0.34 | neg. | -0.10 | 1.32x10^-07^ | 1.89x10^-05^ | 0.07 | 7.85x10^+03^ |
| C14/C16 | 0.39 | 0.06 | neg. | -0.09 | 1.86x10^-07^ | 2.66x10^-05^ | 0.27 | 3.57x10^+05^ |
| C12/C6 (C4:1-DC) | 1.92 | 0.52 | neg. | -0.10 | 2.23x10^-07^ | 3.18x10^-05^ | 0.03 | 4.66x10^+03^ |
| C10/C5 | 3.33 | 1.68 | neg. | -0.10 | 3.20x10^-07^ | 4.58x10^-05^ | 0.13 | 1.83x10^+02^ |
| C0/C12 | 300.33 | 117.20 | pos. | 0.10 | 3.40x10^-07^ | 4.87x10^-05^ | 0.07 | 3.45x10^+02^ |
| C12/C18:1 | 1.02 | 0.32 | neg. | -0.10 | 3.78x10^-07^ | 5.41x10^-05^ | 0.08 | 2.74x10^+03^ |
| C12/C2 | 0.02 | 0.01 | neg. | -0.10 | 7.38x10^-07^ | 1.06x10^-04^ | 0.04 | 1.40x10^+03^ |
| C10/C8 | 1.61 | 0.20 | neg. | -0.09 | 7.00x10^-06^ | 1.00x10^-03^ | 0.05 | 8.32x10^+02^ |
| C14:1-OH/C16 | 0.13 | 0.03 | neg. | -0.09 | 7.38x10^-06^ | 1.06x10^-03^ | 0.02 | 3.26x10^+03^ |
| C16/C7-DC | 2.89 | 0.95 | pos. | 0.08 | 1.37x10^-05^ | 1.96x10^-03^ | 0.10 | 4.52x10^+02^ |
| C18:1/C7-DC | 3.17 | 1.09 | pos. | 0.08 | 1.62x10^-05^ | 2.32x10^-03^ | 0.13 | 3.82x10^+02^ |
| C2/C7-DC | 192.69 | 63.81 | pos. | 0.08 | 1.90x10^-05^ | 2.71x10^-03^ | 0.12 | 3.27x10^+02^ |
| C10/C10:1 | 2.14 | 0.48 | neg. | -0.08 | 2.78x10^-05^ | 3.98x10^-03^ | 0.03 | 2.09x10^+02^ |
| C14:1-OH/C18:1 | 0.12 | 0.03 | neg. | -0.08 | 3.14x10^-05^ | 4.49x10^-03^ | 0.03 | 7.66x10^+02^ |
| C14:1/C18:1 | 1.20 | 0.31 | neg. | -0.07 | 8.34x10^-05^ | 1.19x10^-02^ | 0.35 | 7.25x10^+02^ |
| C14:2/C18:2 | 0.68 | 0.22 | neg. | -0.07 | 1.05x10^-04^ | 1.51x10^-02^ | 0.13 | 2.16x10^+02^ |
| C14:2/C18:1 | 0.24 | 0.08 | neg. | -0.08 | 1.09x10^-04^ | 1.56x10^-02^ | 0.10 | 2.09x10^+02^ |
| C12:1/C18:1 | 1.14 | 0.29 | neg. | -0.08 | 1.16x10^-04^ | 1.66x10^-02^ | 0.08 | 3.81x10^+02^ |
| C14/C18:1 | 0.36 | 0.07 | neg. | -0.07 | 1.26x10^-04^ | 1.80x10^-02^ | 0.20 | 5.30x10^+02^ |
| C12:1/C16 | 1.25 | 0.34 | neg. | -0.08 | 1.34x10^-04^ | 1.91x10^-02^ | 0.06 | 3.31x10^+02^ |
| C6 (C4:1-DC)/C8 | 0.33 | 0.06 | pos. | 0.08 | 1.36x10^-04^ | 1.95x10^-02^ | 0.01 | 1.03x10^+03^ |
| C16/C16:1-OH | 10.99 | 2.14 | pos. | 0.07 | 1.69x10^-04^ | 2.41x10^-02^ | 0.25 | 6.79x10^+02^ |
| C14:1/C16 | 1.31 | 0.34 | neg. | -0.06 | 1.95x10^-04^ | 2.78x10^-02^ | 0.30 | 3.11x10^+02^ |
| C16/C18 | 2.34 | 0.43 | pos. | 0.07 | 2.76x10^-04^ | 3.94x10^-02^ | 0.06 | 4.16x10^+02^ |
| Single PC |  |  |  |  |  |  |  |  |
| PC aa C42:0 | 0.61 | 0.17 | neg. | -0.15 | 9.14x10^-15^ | 1.31x10^-12^ | 0.11 |  |
| PC aa C42:1 | 0.30 | 0.08 | neg. | -0.14 | 8.48x10^-13^ | 1.21x10^-10^ | 0.08 |  |
| PC aa C38:3 | 53.25 | 12.82 | pos. | 0.14 | 3.20x10^-12^ | 4.58x10^-10^ | 0.11 |  |
| PC aa C42:2 | 0.22 | 0.06 | neg. | -0.11 | 3.22x10^-08^ | 4.60x10^-06^ | 0.05 |  |
| PC aa C38:4 | 117.39 | 28.78 | pos. | 0.10 | 1.86x10^-06^ | 2.66x10^-04^ | 0.05 |  |
| PC aa C40:1 | 0.47 | 0.10 | neg. | -0.09 | 4.32x10^-06^ | 6.18x10^-04^ | 0.09 |  |
| PC aa C40:4 | 4.13 | 1.16 | pos. | 0.08 | 5.63x10^-05^ | 8.05x10^-03^ | 0.02 |  |
| PC aa C42:6 | 0.63 | 0.14 | neg. | -0.08 | 1.01x10^-04^ | 1.45x10^-02^ | 0.03 |  |
| PC aa C40:2 | 0.36 | 0.10 | neg. | -0.08 | 1.37x10^-04^ | 1.96x10^-02^ | 0.04 |  |
| PC ae C42:3 | 0.90 | 0.20 | neg. | -0.16 | 1.00x10^-15^ | 1.44x10^-13^ | 0.10 |  |
| PC ae C42:4 | 1.04 | 0.24 | neg. | -0.15 | 2.27x10^-14^ | 3.25x10^-12^ | 0.12 |  |
| PC ae C44:4 | 0.44 | 0.11 | neg. | -0.14 | 7.20x10^-13^ | 1.03x10^-10^ | 0.10 |  |
| PC ae C44:6 | 1.40 | 0.37 | neg. | -0.14 | 7.50x10^-13^ | 1.07x10^-10^ | 0.08 |  |
| PC ae C42:5 | 2.40 | 0.49 | neg. | -0.14 | 2.84x10^-12^ | 4.07x10^-10^ | 0.10 |  |
| PC ae C36:2 | 15.72 | 4.00 | neg. | -0.13 | 3.67x10^-12^ | 5.24x10^-10^ | 0.20 |  |
| PC ae C40:5 | 3.62 | 0.65 | neg. | -0.13 | 2.70x10^-11^ | 3.86x10^-09^ | 0.08 |  |
| PC ae C44:5 | 2.16 | 0.54 | neg. | -0.13 | 1.47x10^-10^ | 2.10x10^-08^ | 0.06 |  |
| PC ae C40:6 | 5.16 | 1.31 | neg. | -0.12 | 2.10x10^-10^ | 3.00x10^-08^ | 0.13 |  |
| PC ae C30:0 | 0.48 | 0.14 | neg. | -0.12 | 3.05x10^-10^ | 4.37x10^-08^ | 0.20 |  |
| PC ae C38:2 | 2.20 | 0.51 | neg. | -0.12 | 1.76x10^-09^ | 2.52x10^-07^ | 0.11 |  |
| PC ae C40:3 | 1.16 | 0.23 | neg. | -0.11 | 3.29x10^-09^ | 4.70x10^-07^ | 0.19 |  |
| PC ae C44:3 | 0.11 | 0.03 | neg. | -0.12 | 4.64x10^-09^ | 6.63x10^-07^ | 0.05 |  |
| PC ae C34:0 | 1.77 | 0.46 | neg. | -0.11 | 9.30x10^-09^ | 1.33x10^-06^ | 0.14 |  |
| PC ae C42:2 | 0.69 | 0.15 | neg. | -0.11 | 5.43x10^-08^ | 7.77x10^-06^ | 0.11 |  |
| PC ae C36:1 | 8.51 | 1.96 | neg. | -0.10 | 8.57x10^-08^ | 1.22x10^-05^ | 0.14 |  |
| PC ae C34:1 | 10.83 | 2.39 | neg. | -0.10 | 1.47x10^-07^ | 2.11x10^-05^ | 0.18 |  |
| PC ae C34:3 | 8.74 | 2.45 | neg. | -0.10 | 4.66x10^-07^ | 6.66x10^-05^ | 0.07 |  |
| PC ae C40:4 | 2.62 | 0.48 | neg. | -0.10 | 9.74x10^-07^ | 1.39x10^-04^ | 0.07 |  |
| PC ae C32:2 | 0.77 | 0.17 | neg. | -0.09 | 1.20x10^-06^ | 1.71x10^-04^ | 0.15 |  |
| PC ae C40:2 | 2.12 | 0.48 | neg. | -0.09 | 1.70x10^-06^ | 2.43x10^-04^ | 0.16 |  |
| PC ae C40:1 | 1.72 | 0.40 | neg. | -0.10 | 2.00x10^-06^ | 2.86x10^-04^ | 0.03 |  |
| PC ae C32:1 | 2.95 | 0.62 | neg. | -0.09 | 4.72x10^-06^ | 6.74x10^-04^ | 0.13 |  |
| PC ae C34:2 | 13.16 | 3.39 | neg. | -0.08 | 1.06x10^-05^ | 1.52x10^-03^ | 0.13 |  |
| PC ae C38:0 | 2.55 | 0.78 | neg. | -0.08 | 5.20x10^-05^ | 7.43x10^-03^ | 0.05 |  |
| PC ae C40:0 | 10.31 | 1.66 | neg. | -0.07 | 1.83x10^-04^ | 2.62x10^-02^ | 0.13 |  |
| ΣPC ae | 199.62 | 34.61 | neg. | -0.08 | 3.29x10^-05^ | 4.71x10^-03^ | 0.11 |  |
| lysoPC a C17:0 | 1.81 | 0.52 | neg. | -0.15 | 1.28x10^-14^ | 1.84x10^-12^ | 0.10 |  |
| lysoPC a C18:1 | 20.26 | 5.87 | neg. | -0.13 | 1.64x10^-12^ | 2.35x10^-10^ | 0.16 |  |
| lysoPC a C18:2 | 29.29 | 9.85 | neg. | -0.13 | 2.36x10^-12^ | 3.37x10^-10^ | 0.19 |  |
| lysoPC a C28:1 | 0.63 | 0.22 | neg. | -0.08 | 2.48x10^-05^ | 3.55x10^-03^ | 0.06 |  |
| ΣlysoPC | 194.05 | 40.53 | neg. | -0.09 | 1.19x10^-06^ | 1.70x10^-04^ | 0.14 |  |
|  |  |  |  |  |  |  |  |  |
| PC aa/PC aa |  |  |  |  |  |  |  |  |
| PC aa C38:3/PC aa C42:6 | 85.92 | 19.67 | pos. | 0.21 | 3.67x10^-29^ | 5.25x10^-27^ | 0.19 | 8.71x10^+16^ |
| PC aa C38:3/PC aa C42:1 | 187.87 | 67.29 | pos. | 0.20 | 4.04x10^-24^ | 5.77x10^-22^ | 0.13 | 2.10x10^+11^ |
| PC aa C38:3/PC aa C42:0 | 95.48 | 37.12 | pos. | 0.20 | 4.59x10^-24^ | 6.56x10^-22^ | 0.14 | 1.99x10^+09^ |
| PC aa C38:4/PC aa C42:1 | 410.11 | 138.19 | pos. | 0.19 | 2.58x10^-21^ | 3.69x10^-19^ | 0.11 | 3.28x10^+08^ |
| PC aa C38:3/PC aa C42:2 | 263.42 | 87.32 | pos. | 0.19 | 4.50x10^-21^ | 6.43x10^-19^ | 0.10 | 7.11x10^+08^ |
| PC aa C38:3/PC aa C42:4 | 243.89 | 55.88 | pos. | 0.18 | 1.40x10^-20^ | 2.01x10^-18^ | 0.11 | 2.28x10^+08^ |
| PC aa C38:4/PC aa C42:0 | 209.21 | 80.15 | pos. | 0.18 | 1.84x10^-20^ | 2.63x10^-18^ | 0.12 | 4.98x10^+05^ |
| PC aa C36:3/PC aa C38:3 | 2.89 | 0.49 | neg. | -0.15 | 1.20x10^-19^ | 1.71x10^-17^ | 0.35 | 2.68x10^+07^ |
| PC aa C28:1/PC aa C38:3 | 0.07 | 0.02 | neg. | -0.18 | 1.87x10^-19^ | 2.68x10^-17^ | 0.11 | 1.71x10^+07^ |
| PC aa C30:0/PC aa C38:3 | 0.09 | 0.03 | neg. | -0.17 | 2.70x10^-19^ | 3.86x10^-17^ | 0.18 | 1.19x10^+07^ |
| PC aa C38:3/PC aa C40:1 | 116.93 | 35.70 | pos. | 0.17 | 2.69x10^-18^ | 3.85x10^-16^ | 0.15 | 1.19x10^+06^ |
| PC aa C32:0/PC aa C38:3 | 0.30 | 0.07 | neg. | -0.17 | 2.81x10^-18^ | 4.02x10^-16^ | 0.16 | 1.14x10^+06^ |
| PC aa C38:3/PC aa C40:2 | 155.08 | 45.98 | pos. | 0.17 | 3.44x10^-18^ | 4.93x10^-16^ | 0.10 | 9.29x10^+05^ |
| PC aa C38:3/PC aa C42:5 | 129.66 | 33.97 | pos. | 0.17 | 3.88x10^-18^ | 5.55x10^-16^ | 0.10 | 8.24x10^+05^ |
| PC aa C38:4/PC aa C42:6 | 189.31 | 43.84 | pos. | 0.17 | 4.26x10^-18^ | 6.09x10^-16^ | 0.11 | 4.36x10^+11^ |
| PC aa C36:1/PC aa C38:3 | 1.04 | 0.19 | neg. | -0.17 | 2.50x10^-17^ | 3.58x10^-15^ | 0.09 | 1.28x10^+05^ |
| PC aa C40:4/PC aa C42:6 | 6.62 | 1.51 | pos. | 0.17 | 6.51x10^-17^ | 9.31x10^-15^ | 0.10 | 8.65x10^+11^ |
| PC aa C32:3/PC aa C38:3 | 0.01 | 0.00 | neg. | -0.16 | 8.02x10^-17^ | 1.15x10^-14^ | 0.14 | 3.99x10^+04^ |
| PC aa C38:4/PC aa C42:2 | 578.15 | 187.71 | pos. | 0.16 | 3.79x10^-16^ | 5.42x10^-14^ | 0.07 | 8.49x10^+07^ |
| PC aa C34:3/PC aa C38:3 | 0.35 | 0.10 | neg. | -0.15 | 6.22x10^-16^ | 8.89x10^-14^ | 0.17 | 5.15x10^+03^ |
| PC aa C38:3/PC aa C38:5 | 0.87 | 0.20 | pos. | 0.16 | 6.62x10^-16^ | 9.47x10^-14^ | 0.14 | 4.83x10^+03^ |
| PC aa C40:4/PC aa C42:1 | 14.58 | 5.79 | pos. | 0.16 | 7.57x10^-16^ | 1.08x10^-13^ | 0.09 | 1.12x10^+03^ |
| PC aa C38:4/PC aa C38:5 | 1.89 | 0.32 | pos. | 0.15 | 2.79x10^-15^ | 3.99x10^-13^ | 0.13 | 6.65x10^+08^ |
| PC aa C36:2/PC aa C38:3 | 4.59 | 0.98 | neg. | -0.14 | 4.80x10^-15^ | 6.87x10^-13^ | 0.21 | 6.66x10^+02^ |
| PC aa C38:4/PC aa C42:4 | 534.83 | 111.92 | pos. | 0.16 | 6.55x10^-15^ | 9.36x10^-13^ | 0.07 | 2.84x10^+08^ |
| PC aa C40:5/PC aa C42:6 | 18.36 | 3.65 | pos. | 0.15 | 4.28x10^-14^ | 6.12x10^-12^ | 0.10 | 2.36x10^+09^ |
| PC aa C38:4/PC aa C40:3 | 181.57 | 42.79 | pos. | 0.15 | 5.40x10^-14^ | 7.73x10^-12^ | 0.07 | 3.44x10^+07^ |
| PC aa C38:4/PC aa C40:1 | 256.42 | 74.82 | pos. | 0.14 | 1.30x10^-13^ | 1.86x10^-11^ | 0.11 | 1.43x10^+07^ |
| PC aa C38:4/PC aa C40:2 | 339.98 | 94.47 | pos. | 0.15 | 1.65x10^-13^ | 2.36x10^-11^ | 0.07 | 1.13x10^+07^ |
| PC aa C38:6/PC aa C40:6 | 3.28 | 0.49 | neg. | -0.13 | 4.37x10^-13^ | 6.25x10^-11^ | 0.25 | 1.80x10^+11^ |
| PC aa C40:4/PC aa C42:2 | 20.46 | 7.50 | pos. | 0.14 | 1.66x10^-12^ | 2.37x10^-10^ | 0.05 | 1.94x10^+04^ |
| PC aa C38:4/PC aa C42:5 | 285.15 | 73.15 | pos. | 0.14 | 6.36x10^-12^ | 9.09x10^-10^ | 0.06 | 2.92x10^+05^ |
| PC aa C40:4/PC aa C42:4 | 18.76 | 4.34 | pos. | 0.14 | 6.99x10^-12^ | 1.00x10^-09^ | 0.07 | 8.05x10^+06^ |
| PC aa C40:3/PC aa C40:4 | 0.17 | 0.05 | neg. | -0.14 | 8.95x10^-12^ | 1.28x10^-09^ | 0.06 | 6.29x10^+06^ |
| PC aa C30:0/PC aa C40:4 | 1.21 | 0.39 | neg. | -0.13 | 3.65x10^-11^ | 5.23x10^-09^ | 0.13 | 1.54x10^+06^ |
| PC aa C40:6/PC aa C42:2 | 135.72 | 41.45 | pos. | 0.13 | 4.42x10^-11^ | 6.32x10^-09^ | 0.08 | 7.28x10^+02^ |
| PC aa C36:4/PC aa C42:2 | 1074.77 | 332.11 | pos. | 0.13 | 6.30x10^-11^ | 9.01x10^-09^ | 0.10 | 5.11x10^+02^ |
| PC aa C32:0/PC aa C38:4 | 0.14 | 0.03 | neg. | -0.13 | 8.37x10^-11^ | 1.20x10^-08^ | 0.10 | 2.22x10^+04^ |
| PC aa C28:1/PC aa C38:4 | 0.03 | 0.01 | neg. | -0.13 | 8.53x10^-11^ | 1.22x10^-08^ | 0.08 | 2.18x10^+04^ |
| PC aa C40:4/PC aa C42:5 | 9.99 | 2.63 | pos. | 0.13 | 9.76x10^-11^ | 1.40x10^-08^ | 0.06 | 5.76x10^+05^ |
| PC aa C40:2/PC aa C40:4 | 0.09 | 0.03 | neg. | -0.13 | 1.17x10^-10^ | 1.68x10^-08^ | 0.05 | 4.79x10^+05^ |
| PC aa C38:0/PC aa C38:4 | 0.03 | 0.01 | neg. | -0.13 | 1.28x10^-10^ | 1.83x10^-08^ | 0.07 | 1.45x10^+04^ |
| PC aa C30:0/PC aa C38:4 | 0.04 | 0.01 | neg. | -0.12 | 2.60x10^-10^ | 3.71x10^-08^ | 0.11 | 7.15x10^+03^ |
| PC aa C40:1/PC aa C40:4 | 0.12 | 0.04 | neg. | -0.12 | 9.92x10^-10^ | 1.42x10^-07^ | 0.07 | 4.36x10^+03^ |
| PC aa C32:3/PC aa C38:4 | 0.00 | 0.00 | neg. | -0.12 | 1.68x10^-09^ | 2.41x10^-07^ | 0.11 | 1.10x10^+03^ |
| PC aa C38:5/PC aa C40:4 | 15.68 | 3.34 | neg. | -0.12 | 1.85x10^-09^ | 2.65x10^-07^ | 0.06 | 3.03x10^+04^ |
| PC aa C32:0/PC aa C40:4 | 3.89 | 0.96 | neg. | -0.12 | 2.09x10^-09^ | 2.99x10^-07^ | 0.08 | 2.69x10^+04^ |
| PC aa C36:4/PC aa C42:6 | 353.04 | 80.73 | pos. | 0.12 | 2.42x10^-09^ | 3.46x10^-07^ | 0.09 | 4.18x10^+04^ |
| PC aa C28:1/PC aa C40:4 | 0.88 | 0.26 | neg. | -0.11 | 5.64x10^-09^ | 8.07x10^-07^ | 0.10 | 9.97x10^+03^ |
| PC aa C40:5/PC aa C42:5 | 27.62 | 6.19 | pos. | 0.12 | 6.12x10^-09^ | 8.75x10^-07^ | 0.05 | 2.05x10^+06^ |
| PC aa C36:0/PC aa C38:4 | 0.02 | 0.01 | neg. | -0.11 | 6.83x10^-09^ | 9.77x10^-07^ | 0.08 | 2.72x10^+02^ |
| PC aa C38:4/PC aa C38:6 | 1.36 | 0.39 | pos. | 0.12 | 7.67x10^-09^ | 1.10x10^-06^ | 0.06 | 2.42x10^+02^ |
| PC aa C40:3/PC aa C40:5 | 0.06 | 0.02 | neg. | -0.11 | 2.11x10^-08^ | 3.01x10^-06^ | 0.04 | 5.79x10^+04^ |
| PC aa C36:3/PC aa C42:6 | 244.39 | 51.71 | pos. | 0.11 | 5.69x10^-08^ | 8.13x10^-06^ | 0.06 | 1.78x10^+03^ |
| PC aa C40:2/PC aa C40:5 | 0.03 | 0.01 | neg. | -0.11 | 6.11x10^-08^ | 8.74x10^-06^ | 0.03 | 2.24x10^+03^ |
| PC aa C32:3/PC aa C40:4 | 0.13 | 0.04 | neg. | -0.10 | 6.75x10^-08^ | 9.66x10^-06^ | 0.13 | 8.33x10^+02^ |
| PC aa C40:6/PC aa C42:6 | 44.99 | 11.65 | pos. | 0.10 | 7.36x10^-08^ | 1.05x10^-05^ | 0.15 | 1.37x10^+03^ |
| PC aa C36:4/PC aa C40:2 | 633.22 | 172.28 | pos. | 0.11 | 9.52x10^-08^ | 1.36x10^-05^ | 0.09 | 1.44x10^+03^ |
| PC aa C34:3/PC aa C40:4 | 4.62 | 1.36 | neg. | -0.10 | 1.07x10^-07^ | 1.52x10^-05^ | 0.11 | 5.28x10^+02^ |
| PC aa C38:0/PC aa C40:6 | 0.12 | 0.03 | neg. | -0.10 | 1.29x10^-07^ | 1.85x10^-05^ | 0.11 | 1.11x10^+04^ |
| PC aa C30:0/PC aa C40:5 | 0.43 | 0.14 | neg. | -0.10 | 1.35x10^-07^ | 1.93x10^-05^ | 0.11 | 1.59x10^+04^ |
| PC aa C30:0/PC aa C36:3 | 0.03 | 0.01 | neg. | -0.10 | 2.06x10^-07^ | 2.94x10^-05^ | 0.05 | 1.04x10^+04^ |
| PC aa C30:0/PC aa C36:4 | 0.02 | 0.01 | neg. | -0.10 | 2.85x10^-07^ | 4.07x10^-05^ | 0.06 | 7.54x10^+03^ |
| PC aa C32:0/PC aa C36:4 | 0.07 | 0.01 | neg. | -0.10 | 3.07x10^-07^ | 4.39x10^-05^ | 0.10 | 8.52x10^+04^ |
| PC aa C36:4/PC aa C42:4 | 998.53 | 211.75 | pos. | 0.10 | 3.74x10^-07^ | 5.35x10^-05^ | 0.16 | 4.53x10^+04^ |
| PC aa C36:4/PC aa C40:3 | 338.74 | 79.41 | pos. | 0.10 | 5.07x10^-07^ | 7.25x10^-05^ | 0.10 | 2.41x10^+03^ |
| PC aa C38:5/PC aa C40:5 | 5.56 | 0.89 | neg. | -0.10 | 8.94x10^-07^ | 1.28x10^-04^ | 0.07 | 1.57x10^+04^ |
| PC aa C36:6/PC aa C40:6 | 0.04 | 0.01 | neg. | -0.10 | 1.12x10^-06^ | 1.61x10^-04^ | 0.06 | 1.02x10^+04^ |
| PC aa C40:5/PC aa C42:4 | 52.43 | 12.16 | pos. | 0.10 | 1.29x10^-06^ | 1.85x10^-04^ | 0.03 | 1.09x10^+04^ |
| PC aa C30:0/PC aa C32:1 | 0.25 | 0.07 | neg. | -0.09 | 1.73x10^-06^ | 2.48x10^-04^ | 0.06 | 1.24x10^+03^ |
| PC aa C36:6/PC aa C40:5 | 0.10 | 0.04 | neg. | -0.09 | 3.42x10^-06^ | 4.89x10^-04^ | 0.06 | 3.36x10^+03^ |
| PC aa C30:0/PC aa C34:4 | 2.22 | 0.57 | neg. | -0.09 | 3.69x10^-06^ | 5.28x10^-04^ | 0.10 | 5.82x10^+02^ |
| PC aa C36:0/PC aa C40:6 | 0.10 | 0.03 | neg. | -0.09 | 3.81x10^-06^ | 5.45x10^-04^ | 0.14 | 3.19x10^+03^ |
| PC aa C40:6/PC aa C42:5 | 67.18 | 16.98 | pos. | 0.09 | 3.83x10^-06^ | 5.47x10^-04^ | 0.08 | 3.28x10^+03^ |
| PC aa C28:1/PC aa C36:3 | 0.02 | 0.01 | neg. | -0.09 | 4.02x10^-06^ | 5.75x10^-04^ | 0.15 | 4.21x10^+02^ |
| PC aa C28:1/PC aa C40:5 | 0.31 | 0.09 | neg. | -0.09 | 4.04x10^-06^ | 5.78x10^-04^ | 0.07 | 4.19x10^+02^ |
| PC aa C36:3/PC aa C40:3 | 234.08 | 48.61 | pos. | 0.09 | 4.65x10^-06^ | 6.65x10^-04^ | 0.08 | 2.62x10^+02^ |
| PC aa C28:1/PC aa C36:4 | 0.02 | 0.00 | neg. | -0.09 | 5.82x10^-06^ | 8.33x10^-04^ | 0.15 | 2.91x10^+02^ |
| PC aa C36:4/PC aa C38:0 | 69.93 | 21.54 | pos. | 0.09 | 8.88x10^-06^ | 1.27x10^-03^ | 0.05 | 1.61x10^+02^ |
| PC aa C36:4/PC aa C42:5 | 533.12 | 140.27 | pos. | 0.09 | 9.54x10^-06^ | 1.36x10^-03^ | 0.08 | 1.32x10^+03^ |
| PC aa C36:4/PC aa C38:5 | 3.52 | 0.54 | pos. | 0.09 | 9.81x10^-06^ | 1.40x10^-03^ | 0.11 | 2.66x10^+03^ |
| PC aa C36:4/PC aa C36:6 | 213.15 | 79.58 | pos. | 0.09 | 1.11x10^-05^ | 1.58x10^-03^ | 0.11 | 1.04x10^+03^ |
| PC aa C34:4/PC aa C36:6 | 2.09 | 0.54 | pos. | 0.09 | 1.30x10^-05^ | 1.86x10^-03^ | 0.03 | 8.82x10^+02^ |
| PC aa C32:0/PC aa C40:5 | 1.39 | 0.33 | neg. | -0.09 | 1.36x10^-05^ | 1.95x10^-03^ | 0.06 | 1.03x10^+03^ |
| PC aa C34:3/PC aa C36:3 | 0.12 | 0.02 | neg. | -0.08 | 1.63x10^-05^ | 2.33x10^-03^ | 0.09 | 4.82x10^+03^ |
| PC aa C32:3/PC aa C36:4 | 0.00 | 0.00 | neg. | -0.08 | 2.65x10^-05^ | 3.79x10^-03^ | 0.20 | 6.37x10^+02^ |
| PC aa C32:3/PC aa C36:3 | 0.00 | 0.00 | neg. | -0.08 | 2.78x10^-05^ | 3.97x10^-03^ | 0.20 | 6.08x10^+02^ |
| PC aa C32:0/PC aa C36:3 | 0.10 | 0.02 | neg. | -0.08 | 2.98x10^-05^ | 4.26x10^-03^ | 0.06 | 2.31x10^+03^ |
| PC aa C36:0/PC aa C36:4 | 0.01 | 0.00 | neg. | -0.08 | 3.04x10^-05^ | 4.34x10^-03^ | 0.03 | 3.99x10^+02^ |
| PC aa C32:3/PC aa C40:5 | 0.04 | 0.01 | neg. | -0.08 | 3.29x10^-05^ | 4.70x10^-03^ | 0.12 | 4.27x10^+02^ |
| PC aa C36:3/PC aa C42:4 | 693.57 | 146.97 | pos. | 0.08 | 6.76x10^-05^ | 9.67x10^-03^ | 0.10 | 2.50x10^+02^ |
| PC aa C36:4/PC aa C38:6 | 2.53 | 0.68 | pos. | 0.08 | 1.10x10^-04^ | 1.57x10^-02^ | 0.07 | 2.38x10^+02^ |
| PC aa C32:2/PC aa C34:4 | 1.78 | 0.50 | neg. | -0.07 | 2.05x10^-04^ | 2.93x10^-02^ | 0.21 | 9.21x10^+02^ |
|  |  |  |  |  |  |  |  |  |
| PC ae/PC ae |  |  |  |  |  |  |  |  |
| PC ae C36:4/PC ae C44:6 | 15.71 | 5.02 | pos. | 0.16 | 1.10x10^-15^ | 1.58x10^-13^ | 0.12 | 6.80x10^+02^ |
| PC ae C36:4/PC ae C42:5 | 8.95 | 2.36 | pos. | 0.16 | 2.13x10^-15^ | 3.05x10^-13^ | 0.11 | 1.33x10^+03^ |
| PC ae C36:4/PC ae C40:6 | 4.24 | 1.20 | pos. | 0.15 | 1.61x10^-14^ | 2.31x10^-12^ | 0.14 | 1.30x10^+04^ |
| PC ae C34:2/PC ae C36:4 | 0.64 | 0.15 | neg. | -0.14 | 4.48x10^-14^ | 6.41x10^-12^ | 0.18 | 2.36x10^+08^ |
| PC ae C36:4/PC ae C44:5 | 10.14 | 3.12 | pos. | 0.15 | 7.90x10^-14^ | 1.13x10^-11^ | 0.08 | 1.86x10^+03^ |
| PC ae C38:6/PC ae C40:6 | 1.73 | 0.29 | pos. | 0.14 | 1.30x10^-13^ | 1.86x10^-11^ | 0.18 | 1.62x10^+03^ |
| PC ae C34:3/PC ae C36:4 | 0.43 | 0.12 | neg. | -0.14 | 7.63x10^-13^ | 1.09x10^-10^ | 0.14 | 6.10x10^+05^ |
| PC ae C36:4/PC ae C40:4 | 8.11 | 1.88 | pos. | 0.14 | 1.61x10^-12^ | 2.31x10^-10^ | 0.15 | 6.04x10^+05^ |
| PC ae C36:4/PC ae C38:5 | 1.04 | 0.10 | pos. | 0.13 | 1.30x10^-11^ | 1.86x10^-09^ | 0.08 | 2.55x10^+09^ |
| PC ae C36:3/PC ae C36:4 | 0.43 | 0.08 | neg. | -0.12 | 1.09x10^-10^ | 1.56x10^-08^ | 0.14 | 3.67x10^+07^ |
| PC ae C36:4/PC ae C40:1 | 12.62 | 3.46 | pos. | 0.13 | 1.21x10^-10^ | 1.73x10^-08^ | 0.07 | 1.65x10^+04^ |
| PC ae C36:4/PC ae C42:2 | 31.47 | 8.87 | pos. | 0.13 | 2.24x10^-10^ | 3.20x10^-08^ | 0.07 | 2.43x10^+02^ |
| PC ae C34:3/PC ae C36:5 | 0.64 | 0.15 | neg. | -0.12 | 3.43x10^-10^ | 4.90x10^-08^ | 0.15 | 1.36x10^+03^ |
| PC ae C36:4/PC ae C38:4 | 1.33 | 0.22 | pos. | 0.12 | 6.24x10^-10^ | 8.92x10^-08^ | 0.13 | 5.32x10^+07^ |
| PC ae C34:1/PC ae C36:4 | 0.54 | 0.15 | neg. | -0.12 | 8.22x10^-10^ | 1.18x10^-07^ | 0.13 | 1.79x10^+02^ |
| PC ae C32:1/PC ae C36:4 | 0.15 | 0.04 | neg. | -0.12 | 8.80x10^-10^ | 1.26x10^-07^ | 0.10 | 5.36x10^+03^ |
| PC ae C32:2/PC ae C36:4 | 0.04 | 0.01 | neg. | -0.11 | 4.64x10^-09^ | 6.64x10^-07^ | 0.16 | 2.58x10^+02^ |
| PC ae C36:4/PC ae C38:6 | 2.43 | 0.49 | pos. | 0.09 | 9.05x10^-07^ | 1.29x10^-04^ | 0.17 | 3.66x10^+04^ |
|  |  |  |  |  |  |  |  |  |
| lysoPC/lysoPC |  |  |  |  |  |  |  |  |
| lysoPC a C18:1/lysoPC a C20:3 | 8.41 | 1.74 | neg. | -0.18 | 1.47x10^-19^ | 2.10x10^-17^ | 0.09 | 1.12x10^+07^ |
|  |  |  |  |  |  |  |  |  |
| PC aa/PC ae |  |  |  |  |  |  |  |  |
| PC aa C38:3/PC ae C42:3 | 61.98 | 19.51 | pos. | 0.22 | 1.09x10^-31^ | 1.55x10^-29^ | 0.18 | 9.25x10^+15^ |
| PC aa C38:3/PC ae C40:3 | 47.15 | 12.34 | pos. | 0.22 | 9.19x10^-31^ | 1.31x10^-28^ | 0.21 | 3.48x10^+18^ |
| PC aa C38:3/PC ae C36:1 | 6.45 | 1.72 | pos. | 0.22 | 4.41x10^-30^ | 6.31x10^-28^ | 0.16 | 7.25x10^+17^ |
| PC aa C38:3/PC ae C38:3 | 12.44 | 2.77 | pos. | 0.21 | 1.83x10^-29^ | 2.62x10^-27^ | 0.19 | 1.75x10^+17^ |
| PC aa C38:3/PC ae C38:2 | 25.07 | 6.88 | pos. | 0.22 | 1.90x10^-29^ | 2.71x10^-27^ | 0.17 | 1.69x10^+17^ |
| PC aa C38:3/PC ae C42:2 | 79.82 | 21.99 | pos. | 0.21 | 3.58x10^-29^ | 5.12x10^-27^ | 0.23 | 8.95x10^+16^ |
| PC aa C38:3/PC ae C44:4 | 128.33 | 43.91 | pos. | 0.21 | 5.73x10^-29^ | 8.20x10^-27^ | 0.20 | 1.25x10^+16^ |
| PC aa C38:3/PC ae C40:5 | 15.04 | 4.11 | pos. | 0.22 | 3.05x10^-28^ | 4.36x10^-26^ | 0.14 | 1.05x10^+16^ |
| PC aa C38:3/PC ae C42:4 | 53.97 | 18.28 | pos. | 0.21 | 3.54x10^-28^ | 5.06x10^-26^ | 0.20 | 6.42x10^+13^ |
| PC aa C38:3/PC ae C34:1 | 5.08 | 1.40 | pos. | 0.20 | 4.06x10^-28^ | 5.81x10^-26^ | 0.24 | 7.88x10^+15^ |
| PC aa C38:3/PC ae C36:2 | 3.60 | 1.31 | pos. | 0.20 | 2.47x10^-27^ | 3.53x10^-25^ | 0.21 | 1.29x10^+15^ |
| PC aa C38:3/PC ae C34:0 | 31.53 | 9.70 | pos. | 0.20 | 5.34x10^-27^ | 7.64x10^-25^ | 0.19 | 5.99x10^+14^ |
| PC aa C38:3/PC ae C44:3 | 494.02 | 150.03 | pos. | 0.20 | 1.18x10^-26^ | 1.69x10^-24^ | 0.17 | 2.71x10^+14^ |
| PC aa C38:4/PC ae C40:5 | 32.85 | 7.81 | pos. | 0.21 | 2.24x10^-26^ | 3.20x10^-24^ | 0.12 | 1.21x10^+15^ |
| PC aa C38:3/PC ae C40:1 | 32.11 | 9.03 | pos. | 0.21 | 3.73x10^-26^ | 5.33x10^-24^ | 0.15 | 8.58x10^+13^ |
| PC aa C38:4/PC ae C40:1 | 69.92 | 15.87 | pos. | 0.20 | 1.76x10^-25^ | 2.51x10^-23^ | 0.13 | 1.06x10^+19^ |
| PC aa C38:3/PC aa C40:3 | 82.26 | 18.55 | pos. | 0.20 | 5.11x10^-25^ | 7.30x10^-23^ | 0.14 | 6.27x10^+12^ |
| PC aa C38:3/PC ae C42:5 | 23.09 | 7.35 | pos. | 0.19 | 2.10x10^-24^ | 3.01x10^-22^ | 0.17 | 1.35x10^+12^ |
| PC aa C38:3/PC ae C40:4 | 20.79 | 5.57 | pos. | 0.20 | 2.72x10^-24^ | 3.89x10^-22^ | 0.16 | 1.18x10^+12^ |
| PC aa C38:4/PC ae C42:3 | 136.35 | 42.29 | pos. | 0.20 | 5.01x10^-24^ | 7.16x10^-22^ | 0.14 | 2.01x10^+08^ |
| PC aa C38:3/PC ae C30:0 | 119.06 | 43.89 | pos. | 0.18 | 6.82x10^-24^ | 9.75x10^-22^ | 0.25 | 4.69x10^+11^ |
| PC aa C38:3/PC ae C40:6 | 10.95 | 3.83 | pos. | 0.19 | 1.56x10^-23^ | 2.22x10^-21^ | 0.15 | 2.06x10^+11^ |
| PC aa C38:3/PC ae C44:5 | 26.19 | 9.39 | pos. | 0.19 | 9.10x10^-23^ | 1.30x10^-20^ | 0.15 | 3.52x10^+10^ |
| PC aa C38:3/PC ae C44:6 | 40.69 | 15.40 | pos. | 0.19 | 9.65x10^-23^ | 1.38x10^-20^ | 0.13 | 7.76x10^+09^ |
| PC aa C36:2/PC ae C36:2 | 15.60 | 3.46 | pos. | 0.18 | 2.99x10^-22^ | 4.28x10^-20^ | 0.20 | 1.23x10^+10^ |
| PC aa C38:3/PC ae C40:0 | 5.24 | 1.29 | pos. | 0.18 | 7.03x10^-22^ | 1.01x10^-19^ | 0.20 | 4.55x10^+09^ |
| PC aa C38:4/PC ae C42:5 | 50.50 | 15.17 | pos. | 0.18 | 1.81x10^-21^ | 2.58x10^-19^ | 0.15 | 1.57x10^+09^ |
| PC aa C38:4/PC ae C42:4 | 118.88 | 41.13 | pos. | 0.18 | 2.49x10^-21^ | 3.56x10^-19^ | 0.15 | 9.11x10^+06^ |
| PC aa C38:3/PC ae C40:2 | 26.13 | 7.85 | pos. | 0.19 | 5.27x10^-21^ | 7.53x10^-19^ | 0.11 | 6.08x10^+08^ |
| PC aa C38:3/PC ae C32:2 | 72.26 | 22.14 | pos. | 0.18 | 6.54x10^-21^ | 9.35x10^-19^ | 0.15 | 4.89x10^+08^ |
| PC aa C38:3/PC ae C32:1 | 18.71 | 5.60 | pos. | 0.18 | 1.43x10^-20^ | 2.04x10^-18^ | 0.17 | 2.24x10^+08^ |
| PC aa C38:4/PC ae C40:4 | 45.49 | 11.00 | pos. | 0.18 | 1.52x10^-20^ | 2.17x10^-18^ | 0.13 | 6.43x10^+13^ |
| PC aa C38:4/PC ae C40:6 | 23.87 | 7.46 | pos. | 0.18 | 1.71x10^-20^ | 2.45x10^-18^ | 0.14 | 1.22x10^+10^ |
| PC aa C38:4/PC ae C44:6 | 88.79 | 32.11 | pos. | 0.18 | 2.17x10^-20^ | 3.11x10^-18^ | 0.12 | 3.45x10^+07^ |
| PC aa C36:2/PC ae C38:2 | 109.88 | 18.12 | pos. | 0.18 | 3.02x10^-20^ | 4.32x10^-18^ | 0.18 | 5.83x10^+10^ |
| PC aa C38:3/PC ae C38:0 | 22.23 | 6.96 | pos. | 0.18 | 3.52x10^-20^ | 5.03x10^-18^ | 0.10 | 9.09x10^+07^ |
| PC aa C38:3/PC ae C42:1 | 144.58 | 33.32 | pos. | 0.17 | 8.59x10^-20^ | 1.23x10^-17^ | 0.15 | 3.73x10^+07^ |
| PC aa C38:4/PC ae C42:2 | 175.65 | 47.68 | pos. | 0.17 | 9.18x10^-20^ | 1.31x10^-17^ | 0.17 | 5.92x10^+11^ |
| PC aa C36:3/PC ae C36:2 | 10.05 | 2.86 | pos. | 0.17 | 2.12x10^-19^ | 3.03x10^-17^ | 0.17 | 1.73x10^+07^ |
| PC aa C38:4/PC ae C44:5 | 57.29 | 19.78 | pos. | 0.17 | 3.76x10^-19^ | 5.38x10^-17^ | 0.12 | 3.91x10^+08^ |
| PC aa C38:4/PC ae C44:4 | 284.94 | 107.16 | pos. | 0.17 | 6.67x10^-19^ | 9.54x10^-17^ | 0.13 | 1.08x10^+06^ |
| PC aa C40:4/PC ae C40:5 | 1.16 | 0.34 | pos. | 0.18 | 1.11x10^-18^ | 1.59x10^-16^ | 0.10 | 2.43x10^+07^ |
| PC aa C40:4/PC ae C42:4 | 4.18 | 1.63 | pos. | 0.17 | 1.18x10^-18^ | 1.68x10^-16^ | 0.13 | 1.93x10^+04^ |
| PC aa C38:4/PC ae C36:2 | 7.97 | 3.01 | pos. | 0.17 | 1.60x10^-18^ | 2.29x10^-16^ | 0.16 | 2.29x10^+06^ |
| PC aa C40:4/PC ae C42:3 | 4.82 | 1.73 | pos. | 0.17 | 2.17x10^-18^ | 3.10x10^-16^ | 0.11 | 4.64x10^+02^ |
| PC aa C34:2/PC ae C36:2 | 26.32 | 6.53 | pos. | 0.14 | 2.57x10^-18^ | 3.68x10^-16^ | 0.38 | 1.42x10^+06^ |
| PC aa C40:6/PC ae C40:6 | 5.58 | 1.57 | pos. | 0.16 | 4.10x10^-18^ | 5.87x10^-16^ | 0.19 | 5.11x10^+07^ |
| PC aa C36:3/PC ae C42:3 | 174.42 | 45.25 | pos. | 0.17 | 5.62x10^-18^ | 8.03x10^-16^ | 0.10 | 1.79x10^+02^ |
| PC aa C36:2/PC ae C42:3 | 272.07 | 61.81 | pos. | 0.17 | 6.60x10^-18^ | 9.44x10^-16^ | 0.10 | 1.52x10^+02^ |
| PC aa C40:4/PC ae C44:4 | 9.98 | 4.04 | pos. | 0.17 | 8.47x10^-18^ | 1.21x10^-15^ | 0.12 | 8.49x10^+04^ |
| PC aa C38:4/PC ae C40:3 | 104.47 | 29.93 | pos. | 0.17 | 8.54x10^-18^ | 1.22x10^-15^ | 0.14 | 3.85x10^+08^ |
| PC aa C36:3/PC ae C38:2 | 70.54 | 15.38 | pos. | 0.16 | 9.88x10^-18^ | 1.41x10^-15^ | 0.16 | 1.78x10^+08^ |
| PC aa C36:4/PC ae C40:1 | 129.94 | 27.23 | pos. | 0.16 | 1.33x10^-17^ | 1.90x10^-15^ | 0.15 | 1.50x10^+11^ |
| PC aa C38:3/PC ae C34:2 | 4.32 | 1.60 | pos. | 0.16 | 1.51x10^-17^ | 2.16x10^-15^ | 0.17 | 2.12x10^+05^ |
| PC aa C38:4/PC ae C44:3 | 1091.87 | 351.39 | pos. | 0.17 | 2.13x10^-17^ | 3.04x10^-15^ | 0.11 | 2.18x10^+08^ |
| PC aa C38:3/PC ae C36:0 | 52.49 | 15.87 | pos. | 0.16 | 2.16x10^-17^ | 3.09x10^-15^ | 0.14 | 1.48x10^+05^ |
| PC aa C38:3/PC ae C34:3 | 6.58 | 2.54 | pos. | 0.17 | 2.38x10^-17^ | 3.41x10^-15^ | 0.11 | 1.34x10^+05^ |
| PC aa C40:4/PC ae C42:2 | 6.16 | 1.80 | pos. | 0.16 | 3.46x10^-17^ | 4.95x10^-15^ | 0.14 | 1.57x10^+09^ |
| PC aa C38:4/PC ae C38:2 | 55.70 | 17.18 | pos. | 0.17 | 4.37x10^-17^ | 6.25x10^-15^ | 0.11 | 4.03x10^+07^ |
| PC aa C38:3/PC ae C36:3 | 6.30 | 1.93 | pos. | 0.16 | 5.16x10^-17^ | 7.38x10^-15^ | 0.20 | 6.20x10^+04^ |
| PC aa C38:4/PC ae C34:0 | 69.86 | 22.89 | pos. | 0.16 | 5.83x10^-17^ | 8.34x10^-15^ | 0.13 | 1.60x10^+08^ |
| PC aa C40:4/PC ae C36:2 | 0.28 | 0.12 | pos. | 0.16 | 6.42x10^-17^ | 9.17x10^-15^ | 0.16 | 5.71x10^+04^ |
| PC aa C38:4/PC ae C36:1 | 14.32 | 4.26 | pos. | 0.16 | 1.09x10^-16^ | 1.55x10^-14^ | 0.10 | 7.88x10^+08^ |
| PC ae C36:4/PC ae C40:5 | 5.87 | 1.39 | pos. | 0.16 | 1.38x10^-16^ | 1.97x10^-14^ | 0.18 | 1.96x10^+05^ |
| PC aa C40:4/PC ae C38:2 | 1.95 | 0.64 | pos. | 0.16 | 1.52x10^-16^ | 2.18x10^-14^ | 0.12 | 1.16x10^+07^ |
| PC aa C40:4/PC ae C40:4 | 1.60 | 0.47 | pos. | 0.16 | 2.65x10^-16^ | 3.79x10^-14^ | 0.11 | 3.68x10^+09^ |
| PC aa C38:3/PC ae C42:0 | 107.87 | 30.84 | pos. | 0.15 | 2.71x10^-16^ | 3.87x10^-14^ | 0.19 | 1.18x10^+04^ |
| PC aa C38:4/PC ae C38:4 | 7.52 | 1.73 | pos. | 0.15 | 3.51x10^-16^ | 5.01x10^-14^ | 0.20 | 5.30x10^+09^ |
| PC aa C40:4/PC ae C36:1 | 0.50 | 0.16 | pos. | 0.16 | 3.61x10^-16^ | 5.16x10^-14^ | 0.13 | 2.38x10^+08^ |
| PC aa C40:4/PC ae C34:1 | 0.39 | 0.12 | pos. | 0.15 | 4.01x10^-16^ | 5.74x10^-14^ | 0.17 | 3.67x10^+08^ |
| PC aa C40:4/PC ae C34:0 | 2.45 | 0.85 | pos. | 0.16 | 4.35x10^-16^ | 6.22x10^-14^ | 0.14 | 2.14x10^+07^ |
| PC aa C40:4/PC ae C40:1 | 2.47 | 0.71 | pos. | 0.16 | 5.93x10^-16^ | 8.49x10^-14^ | 0.07 | 3.37x10^+09^ |
| PC aa C38:3/PC ae C38:4 | 3.46 | 0.99 | pos. | 0.15 | 8.80x10^-16^ | 1.26x10^-13^ | 0.19 | 3.64x10^+03^ |
| PC aa C40:4/PC ae C42:5 | 1.78 | 0.63 | pos. | 0.16 | 1.13x10^-15^ | 1.61x10^-13^ | 0.11 | 2.52x10^+03^ |
| PC aa C40:4/PC ae C44:3 | 38.28 | 12.76 | pos. | 0.16 | 1.23x10^-15^ | 1.77x10^-13^ | 0.09 | 3.76x10^+06^ |
| PC aa C34:2/PC ae C38:2 | 186.71 | 44.17 | pos. | 0.12 | 1.78x10^-15^ | 2.55x10^-13^ | 0.46 | 9.89x10^+05^ |
| PC aa C38:4/PC ae C34:1 | 11.27 | 3.44 | pos. | 0.15 | 2.11x10^-15^ | 3.02x10^-13^ | 0.15 | 6.97x10^+07^ |
| PC aa C40:4/PC ae C40:3 | 3.67 | 1.19 | pos. | 0.15 | 2.53x10^-15^ | 3.62x10^-13^ | 0.15 | 1.30x10^+06^ |
| PC aa C42:1/PC ae C36:4 | 0.02 | 0.00 | neg. | -0.15 | 2.54x10^-15^ | 3.63x10^-13^ | 0.12 | 3.34x10^+02^ |
| PC aa C36:3/PC ae C44:4 | 360.79 | 104.54 | pos. | 0.16 | 2.68x10^-15^ | 3.83x10^-13^ | 0.09 | 2.69x10^+02^ |
| PC aa C40:4/PC ae C44:6 | 3.15 | 1.33 | pos. | 0.16 | 2.86x10^-15^ | 4.09x10^-13^ | 0.09 | 2.62x10^+02^ |
| PC aa C36:4/PC ae C40:5 | 61.46 | 15.62 | pos. | 0.15 | 3.15x10^-15^ | 4.51x10^-13^ | 0.18 | 8.57x10^+03^ |
| PC aa C40:4/PC ae C30:0 | 9.27 | 3.93 | pos. | 0.15 | 3.33x10^-15^ | 4.76x10^-13^ | 0.18 | 9.18x10^+04^ |
| PC aa C36:3/PC ae C34:1 | 14.24 | 2.82 | pos. | 0.15 | 5.47x10^-15^ | 7.83x10^-13^ | 0.14 | 2.69x10^+07^ |
| PC aa C38:4/PC ae C38:0 | 48.82 | 14.76 | pos. | 0.16 | 7.17x10^-15^ | 1.03x10^-12^ | 0.08 | 2.59x10^+08^ |
| PC aa C36:3/PC ae C36:1 | 18.24 | 4.14 | pos. | 0.14 | 8.26x10^-15^ | 1.18x10^-12^ | 0.22 | 1.04x10^+07^ |
| PC aa C38:3/PC ae C38:1 | 70.46 | 26.94 | pos. | 0.16 | 9.26x10^-15^ | 1.32x10^-12^ | 0.07 | 3.46x10^+02^ |
| PC aa C40:4/PC ae C44:5 | 2.02 | 0.80 | pos. | 0.15 | 9.37x10^-15^ | 1.34x10^-12^ | 0.09 | 1.57x10^+04^ |
| PC aa C38:4/PC ae C30:0 | 264.99 | 107.03 | pos. | 0.15 | 9.92x10^-15^ | 1.42x10^-12^ | 0.17 | 3.08x10^+04^ |
| PC aa C38:4/PC ae C32:2 | 159.11 | 49.29 | pos. | 0.15 | 1.06x10^-14^ | 1.51x10^-12^ | 0.12 | 1.13x10^+08^ |
| PC aa C38:4/PC ae C40:0 | 11.50 | 2.65 | pos. | 0.15 | 1.22x10^-14^ | 1.75x10^-12^ | 0.14 | 1.52x10^+08^ |
| PC aa C38:4/PC ae C40:2 | 57.61 | 17.37 | pos. | 0.15 | 1.78x10^-14^ | 2.55x10^-12^ | 0.09 | 9.55x10^+07^ |
| PC aa C38:4/PC ae C32:1 | 41.25 | 12.65 | pos. | 0.15 | 2.22x10^-14^ | 3.18x10^-12^ | 0.13 | 8.36x10^+07^ |
| PC aa C40:5/PC ae C42:2 | 17.08 | 4.43 | pos. | 0.15 | 2.47x10^-14^ | 3.54x10^-12^ | 0.15 | 2.20x10^+06^ |
| PC aa C36:3/PC ae C34:0 | 88.54 | 21.38 | pos. | 0.15 | 3.18x10^-14^ | 4.55x10^-12^ | 0.11 | 2.92x10^+05^ |
| PC aa C40:4/PC ae C40:6 | 0.85 | 0.34 | pos. | 0.15 | 5.04x10^-14^ | 7.20x10^-12^ | 0.11 | 4.16x10^+03^ |
| PC aa C38:4/PC ae C42:1 | 316.84 | 65.17 | pos. | 0.15 | 6.00x10^-14^ | 8.58x10^-12^ | 0.10 | 3.10x10^+07^ |
| PC aa C38:4/PC ae C34:3 | 14.45 | 5.52 | pos. | 0.15 | 1.62x10^-13^ | 2.32x10^-11^ | 0.09 | 2.87x10^+06^ |
| PC aa C36:4/PC ae C40:6 | 44.41 | 13.52 | pos. | 0.14 | 1.74x10^-13^ | 2.49x10^-11^ | 0.11 | 1.20x10^+03^ |
| PC aa C34:1/PC ae C34:1 | 22.79 | 4.59 | pos. | 0.13 | 2.55x10^-13^ | 3.65x10^-11^ | 0.22 | 5.77x10^+05^ |
| PC aa C36:3/PC ae C42:2 | 224.74 | 48.80 | pos. | 0.15 | 3.17x10^-13^ | 4.54x10^-11^ | 0.06 | 1.71x10^+05^ |
| PC aa C36:4/PC ae C42:2 | 325.38 | 79.48 | pos. | 0.15 | 3.33x10^-13^ | 4.76x10^-11^ | 0.08 | 1.63x10^+05^ |
| PC aa C36:4/PC ae C34:0 | 128.93 | 37.64 | pos. | 0.14 | 4.17x10^-13^ | 5.96x10^-11^ | 0.12 | 2.23x10^+04^ |
| PC aa C36:3/PC ae C30:0 | 332.08 | 98.14 | pos. | 0.14 | 4.49x10^-13^ | 6.42x10^-11^ | 0.11 | 6.80x10^+02^ |
| PC aa C36:3/PC ae C40:3 | 133.46 | 30.09 | pos. | 0.14 | 5.82x10^-13^ | 8.33x10^-11^ | 0.18 | 5.65x10^+03^ |
| PC aa C40:5/PC ae C40:1 | 6.87 | 1.77 | pos. | 0.14 | 1.12x10^-12^ | 1.60x10^-10^ | 0.07 | 1.79x10^+06^ |
| PC aa C40:5/PC ae C40:6 | 2.35 | 0.82 | pos. | 0.14 | 1.27x10^-12^ | 1.81x10^-10^ | 0.10 | 1.66x10^+02^ |
| PC aa C40:5/PC ae C34:0 | 6.79 | 2.21 | pos. | 0.14 | 1.27x10^-12^ | 1.82x10^-10^ | 0.12 | 7.31x10^+03^ |
| PC aa C40:6/PC ae C38:0 | 11.37 | 2.82 | pos. | 0.14 | 2.06x10^-12^ | 2.95x10^-10^ | 0.11 | 2.52x10^+07^ |
| PC aa C40:4/PC ae C32:1 | 1.45 | 0.50 | pos. | 0.14 | 2.32x10^-12^ | 3.31x10^-10^ | 0.11 | 2.03x10^+06^ |
| PC aa C36:1/PC ae C36:1 | 6.54 | 1.57 | pos. | 0.13 | 2.36x10^-12^ | 3.37x10^-10^ | 0.14 | 3.63x10^+04^ |
| PC aa C38:4/PC ae C34:2 | 9.51 | 3.57 | pos. | 0.14 | 3.02x10^-12^ | 4.32x10^-10^ | 0.13 | 6.15x10^+05^ |
| PC aa C40:5/PC ae C44:3 | 106.36 | 33.39 | pos. | 0.14 | 3.31x10^-12^ | 4.73x10^-10^ | 0.09 | 1.40x10^+03^ |
| PC aa C34:2/PC ae C34:0 | 233.74 | 58.43 | pos. | 0.12 | 8.33x10^-12^ | 1.19x10^-09^ | 0.31 | 1.12x10^+03^ |
| PC aa C40:4/PC ae C42:1 | 11.08 | 2.34 | pos. | 0.14 | 1.22x10^-11^ | 1.74x10^-09^ | 0.07 | 4.62x10^+06^ |
| PC aa C36:3/PC ae C44:3 | 1398.09 | 384.63 | pos. | 0.13 | 1.52x10^-11^ | 2.18x10^-09^ | 0.07 | 3.04x10^+02^ |
| PC aa C40:5/PC ae C36:1 | 1.40 | 0.42 | pos. | 0.13 | 1.69x10^-11^ | 2.41x10^-09^ | 0.11 | 5.08x10^+03^ |
| PC aa C40:4/PC ae C32:2 | 5.62 | 2.01 | pos. | 0.13 | 1.82x10^-11^ | 2.60x10^-09^ | 0.12 | 6.59x10^+04^ |
| PC aa C36:1/PC ae C34:0 | 31.89 | 8.56 | pos. | 0.12 | 1.93x10^-11^ | 2.77x10^-09^ | 0.20 | 4.81x10^+02^ |
| PC aa C36:3/PC ae C40:1 | 90.72 | 21.49 | pos. | 0.13 | 2.20x10^-11^ | 3.15x10^-09^ | 0.08 | 9.09x10^+04^ |
| PC aa C36:4/PC ae C34:1 | 20.78 | 5.50 | pos. | 0.13 | 2.27x10^-11^ | 3.25x10^-09^ | 0.12 | 6.49x10^+03^ |
| PC aa C38:4/PC ae C36:0 | 115.73 | 35.30 | pos. | 0.13 | 2.28x10^-11^ | 3.27x10^-09^ | 0.09 | 8.13x10^+04^ |
| PC aa C36:4/PC ae C36:1 | 26.67 | 7.79 | pos. | 0.12 | 3.61x10^-11^ | 5.17x10^-09^ | 0.19 | 2.37x10^+03^ |
| PC aa C36:2/PC ae C34:0 | 138.87 | 31.74 | pos. | 0.13 | 3.89x10^-11^ | 5.56x10^-09^ | 0.08 | 2.39x10^+02^ |
| PC aa C34:1/PC ae C34:0 | 141.93 | 35.69 | pos. | 0.13 | 4.12x10^-11^ | 5.89x10^-09^ | 0.14 | 2.26x10^+02^ |
| PC aa C36:2/PC ae C42:2 | 351.86 | 69.37 | pos. | 0.13 | 4.31x10^-11^ | 6.17x10^-09^ | 0.05 | 1.26x10^+03^ |
| PC aa C40:4/PC ae C34:3 | 0.51 | 0.22 | pos. | 0.13 | 4.62x10^-11^ | 6.60x10^-09^ | 0.08 | 1.01x10^+04^ |
| PC aa C38:4/PC ae C38:3 | 27.78 | 7.79 | pos. | 0.13 | 5.18x10^-11^ | 7.40x10^-09^ | 0.09 | 3.59x10^+04^ |
| PC aa C40:4/PC ae C38:0 | 1.73 | 0.62 | pos. | 0.13 | 5.37x10^-11^ | 7.68x10^-09^ | 0.07 | 9.67x10^+05^ |
| PC aa C34:2/PC ae C34:1 | 37.62 | 7.99 | pos. | 0.11 | 6.10x10^-11^ | 8.72x10^-09^ | 0.36 | 2.41x10^+03^ |
| PC aa C36:2/PC ae C36:1 | 28.60 | 6.02 | pos. | 0.12 | 6.15x10^-11^ | 8.80x10^-09^ | 0.18 | 1.39x10^+03^ |
| PC aa C36:4/PC ae C40:4 | 84.85 | 20.53 | pos. | 0.13 | 7.00x10^-11^ | 1.00x10^-08^ | 0.13 | 1.39x10^+04^ |
| PC aa C36:1/PC ae C34:1 | 5.14 | 1.19 | pos. | 0.11 | 7.80x10^-11^ | 1.12x10^-08^ | 0.29 | 1.89x10^+03^ |
| PC aa C40:4/PC ae C34:2 | 0.34 | 0.14 | pos. | 0.13 | 8.37x10^-11^ | 1.20x10^-08^ | 0.11 | 1.27x10^+05^ |
| PC aa C40:5/PC ae C34:1 | 1.10 | 0.33 | pos. | 0.12 | 1.12x10^-10^ | 1.60x10^-08^ | 0.14 | 1.32x10^+03^ |
| PC aa C38:4/PC ae C42:0 | 237.08 | 66.26 | pos. | 0.12 | 1.46x10^-10^ | 2.09x10^-08^ | 0.13 | 1.27x10^+04^ |
| PC aa C40:4/PC ae C36:0 | 4.06 | 1.30 | pos. | 0.13 | 2.07x10^-10^ | 2.96x10^-08^ | 0.07 | 2.71x10^+05^ |
| PC aa C40:4/PC ae C38:4 | 0.27 | 0.08 | pos. | 0.12 | 2.36x10^-10^ | 3.38x10^-08^ | 0.12 | 2.38x10^+05^ |
| PC aa C34:1/PC ae C36:1 | 29.37 | 7.64 | pos. | 0.11 | 2.90x10^-10^ | 4.15x10^-08^ | 0.29 | 2.95x10^+02^ |
| PC aa C40:4/PC ae C38:3 | 0.97 | 0.30 | pos. | 0.12 | 3.42x10^-10^ | 4.90x10^-08^ | 0.13 | 1.64x10^+05^ |
| PC aa C38:4/PC ae C38:5 | 5.93 | 1.30 | pos. | 0.12 | 3.58x10^-10^ | 5.12x10^-08^ | 0.15 | 5.18x10^+03^ |
| PC aa C36:4/PC ae C32:1 | 76.06 | 20.02 | pos. | 0.12 | 3.60x10^-10^ | 5.15x10^-08^ | 0.08 | 1.31x10^+04^ |
| PC aa C40:4/PC ae C40:2 | 2.05 | 0.75 | pos. | 0.12 | 3.72x10^-10^ | 5.32x10^-08^ | 0.10 | 4.57x10^+03^ |
| PC aa C38:4/PC ae C36:3 | 13.93 | 4.52 | pos. | 0.12 | 3.87x10^-10^ | 5.54x10^-08^ | 0.13 | 4.80x10^+03^ |
| PC aa C40:5/PC ae C38:0 | 4.76 | 1.44 | pos. | 0.12 | 4.42x10^-10^ | 6.32x10^-08^ | 0.07 | 1.17x10^+05^ |
| PC aa C40:4/PC ae C40:0 | 0.41 | 0.11 | pos. | 0.12 | 5.26x10^-10^ | 7.52x10^-08^ | 0.09 | 1.07x10^+05^ |
| PC aa C36:4/PC ae C38:0 | 90.74 | 26.10 | pos. | 0.12 | 5.89x10^-10^ | 8.43x10^-08^ | 0.14 | 8.82x10^+04^ |
| PC aa C36:2/PC ae C34:1 | 22.38 | 4.35 | pos. | 0.12 | 6.07x10^-10^ | 8.69x10^-08^ | 0.09 | 2.42x10^+02^ |
| PC aa C38:4/PC ae C38:1 | 156.15 | 63.36 | pos. | 0.12 | 7.08x10^-10^ | 1.01x10^-07^ | 0.04 | 2.62x10^+03^ |
| PC aa C36:2/PC ae C34:3 | 28.47 | 7.42 | pos. | 0.12 | 1.18x10^-09^ | 1.68x10^-07^ | 0.07 | 3.96x10^+02^ |
| PC aa C38:5/PC ae C40:1 | 37.09 | 6.37 | pos. | 0.12 | 1.35x10^-09^ | 1.93x10^-07^ | 0.05 | 1.48x10^+03^ |
| PC aa C36:4/PC ae C32:2 | 295.14 | 84.94 | pos. | 0.11 | 1.42x10^-09^ | 2.03x10^-07^ | 0.15 | 8.43x10^+02^ |
| PC aa C38:4/PC ae C38:6 | 13.79 | 3.62 | pos. | 0.12 | 1.50x10^-09^ | 2.15x10^-07^ | 0.04 | 1.24x10^+03^ |
| PC aa C40:6/PC ae C40:1 | 16.77 | 4.89 | pos. | 0.12 | 2.14x10^-09^ | 3.05x10^-07^ | 0.13 | 9.37x10^+02^ |
| PC aa C36:4/PC ae C34:3 | 26.70 | 9.31 | pos. | 0.12 | 2.38x10^-09^ | 3.41x10^-07^ | 0.09 | 1.95x10^+02^ |
| PC aa C36:2/PC ae C40:1 | 142.13 | 31.74 | pos. | 0.12 | 2.42x10^-09^ | 3.47x10^-07^ | 0.08 | 8.25x10^+02^ |
| PC aa C40:4/PC ae C36:3 | 0.49 | 0.17 | pos. | 0.12 | 2.87x10^-09^ | 4.10x10^-07^ | 0.11 | 1.96x10^+04^ |
| PC aa C36:3/PC ae C32:1 | 52.49 | 11.97 | pos. | 0.12 | 3.48x10^-09^ | 4.98x10^-07^ | 0.05 | 1.35x10^+03^ |
| PC aa C40:5/PC ae C40:4 | 4.49 | 1.33 | pos. | 0.11 | 5.77x10^-09^ | 8.25x10^-07^ | 0.08 | 1.69x10^+02^ |
| PC aa C40:4/PC ae C38:1 | 5.47 | 2.25 | pos. | 0.12 | 7.38x10^-09^ | 1.06x10^-06^ | 0.04 | 7.63x10^+03^ |
| PC aa C36:4/PC ae C40:2 | 107.63 | 32.61 | pos. | 0.11 | 8.06x10^-09^ | 1.15x10^-06^ | 0.21 | 2.11x10^+02^ |
| PC aa C36:2/PC ae C34:2 | 18.66 | 4.22 | pos. | 0.11 | 9.04x10^-09^ | 1.29x10^-06^ | 0.08 | 1.17x10^+03^ |
| PC aa C36:3/PC ae C34:2 | 12.04 | 3.46 | pos. | 0.11 | 2.31x10^-08^ | 3.30x10^-06^ | 0.07 | 4.59x10^+02^ |
| PC aa C40:5/PC ae C32:1 | 4.04 | 1.36 | pos. | 0.11 | 2.92x10^-08^ | 4.17x10^-06^ | 0.10 | 1.62x10^+02^ |
| PC aa C40:4/PC ae C42:0 | 8.36 | 2.61 | pos. | 0.11 | 3.18x10^-08^ | 4.55x10^-06^ | 0.09 | 1.77x10^+03^ |
| PC aa C36:3/PC ae C38:0 | 63.00 | 17.90 | pos. | 0.11 | 5.51x10^-08^ | 7.88x10^-06^ | 0.09 | 9.42x10^+02^ |
| PC aa C38:0/PC ae C36:4 | 0.16 | 0.04 | neg. | -0.11 | 5.72x10^-08^ | 8.18x10^-06^ | 0.08 | 2.50x10^+04^ |
| PC aa C36:4/PC ae C34:2 | 17.54 | 5.88 | pos. | 0.11 | 5.80x10^-08^ | 8.30x10^-06^ | 0.07 | 1.83x10^+02^ |
| PC aa C36:4/PC ae C36:0 | 213.53 | 57.08 | pos. | 0.11 | 8.78x10^-08^ | 1.26x10^-05^ | 0.04 | 7.26x10^+03^ |
| PC aa C38:5/PC ae C38:0 | 25.56 | 5.29 | pos. | 0.10 | 9.73x10^-08^ | 1.39x10^-05^ | 0.09 | 5.34x10^+02^ |
| PC aa C40:5/PC ae C40:0 | 1.12 | 0.27 | pos. | 0.10 | 1.43x10^-07^ | 2.04x10^-05^ | 0.10 | 1.28x10^+03^ |
| PC aa C40:5/PC ae C36:0 | 11.29 | 3.44 | pos. | 0.10 | 1.87x10^-07^ | 2.67x10^-05^ | 0.06 | 3.41x10^+03^ |
| PC aa C36:4/PC ae C40:0 | 21.34 | 4.43 | pos. | 0.10 | 2.16x10^-07^ | 3.09x10^-05^ | 0.05 | 8.49x10^+02^ |
| PC aa C36:3/PC ae C38:3 | 35.36 | 7.00 | pos. | 0.09 | 2.36x10^-07^ | 3.38x10^-05^ | 0.24 | 6.27x10^+04^ |
| PC aa C36:4/PC ae C38:4 | 13.98 | 2.94 | pos. | 0.10 | 3.93x10^-07^ | 5.62x10^-05^ | 0.05 | 6.65x10^+04^ |
| PC aa C40:2/PC ae C36:4 | 0.02 | 0.01 | neg. | -0.10 | 8.13x10^-07^ | 1.16x10^-04^ | 0.06 | 1.68x10^+02^ |
| PC aa C40:5/PC ae C38:1 | 15.20 | 5.95 | pos. | 0.10 | 1.75x10^-06^ | 2.51x10^-04^ | 0.03 | 3.59x10^+02^ |
| PC aa C40:5/PC ae C42:1 | 31.02 | 6.78 | pos. | 0.09 | 3.24x10^-06^ | 4.64x10^-04^ | 0.05 | 4.33x10^+03^ |
| PC aa C36:3/PC ae C36:0 | 147.54 | 35.81 | pos. | 0.10 | 5.64x10^-07^ | 8.06x10^-05^ | 0.03 | 1.13x10^+03^ |
| PC aa C36:3/PC ae C36:3 | 17.63 | 4.00 | pos. | 0.09 | 4.29x10^-06^ | 6.13x10^-04^ | 0.04 | 9.33x10^+02^ |
| PC aa C40:5/PC ae C42:0 | 23.16 | 6.57 | pos. | 0.09 | 6.25x10^-06^ | 8.93x10^-04^ | 0.10 | 4.76x10^+02^ |
| PC aa C36:0/PC ae C36:4 | 0.13 | 0.04 | neg. | -0.09 | 9.53x10^-06^ | 1.36x10^-03^ | 0.05 | 1.27x10^+03^ |
| PC aa C40:5/PC ae C38:3 | 2.72 | 0.82 | pos. | 0.08 | 1.45x10^-05^ | 2.07x10^-03^ | 0.09 | 9.69x10^+02^ |
| PC aa C36:4/PC ae C36:3 | 25.72 | 7.39 | pos. | 0.09 | 1.53x10^-05^ | 2.18x10^-03^ | 0.04 | 2.62x10^+02^ |
| PC aa C36:4/PC ae C42:1 | 591.88 | 126.66 | pos. | 0.08 | 2.30x10^-05^ | 3.29x10^-03^ | 0.08 | 1.14x10^+03^ |
| PC aa C42:4/PC ae C36:4 | 0.01 | 0.00 | neg. | -0.08 | 2.77x10^-05^ | 3.96x10^-03^ | 0.10 | 6.11x10^+02^ |
| PC aa C36:4/PC ae C38:3 | 51.94 | 15.10 | pos. | 0.08 | 4.65x10^-05^ | 6.66x10^-03^ | 0.17 | 3.19x10^+02^ |
| PC aa C36:4/PC ae C38:6 | 25.75 | 6.77 | pos. | 0.07 | 1.69x10^-04^ | 2.42x10^-02^ | 0.12 | 1.55x10^+02^ |
|  |  |  |  |  |  |  |  |  |
| PC/lysoPC |  |  |  |  |  |  |  |  |
| PC aa C38:3/lysoPC a C17:0 | 32.40 | 14.58 | pos. | 0.20 | 3.41x10^-24^ | 4.87x10^-22^ | 0.12 | 3.77x10^+09^ |
| PC aa C38:3/lysoPC a C18:1 | 2.86 | 1.12 | pos. | 0.19 | 1.58x10^-23^ | 2.26x10^-21^ | 0.21 | 1.04x10^+11^ |
| PC aa C38:3/lysoPC a C18:2 | 2.07 | 0.97 | pos. | 0.17 | 3.61x10^-19^ | 5.16x10^-17^ | 0.21 | 6.54x10^+06^ |
| PC aa C38:4/lysoPC a C17:0 | 71.31 | 31.03 | pos. | 0.17 | 3.19x10^-18^ | 4.56x10^-16^ | 0.09 | 4.03x10^+03^ |
| PC aa C38:4/lysoPC a C18:1 | 6.28 | 2.45 | pos. | 0.16 | 7.03x10^-18^ | 1.00x10^-15^ | 0.16 | 2.34x10^+05^ |
| PC aa C40:4/lysoPC a C18:1 | 0.22 | 0.08 | pos. | 0.16 | 2.74x10^-17^ | 3.91x10^-15^ | 0.14 | 6.01x10^+04^ |
| PC aa C38:3/lysoPC a C16:0 | 0.57 | 0.17 | pos. | 0.15 | 8.84x10^-16^ | 1.26x10^-13^ | 0.16 | 3.62x10^+03^ |
| PC aa C38:4/lysoPC a C18:2 | 4.54 | 2.14 | pos. | 0.15 | 3.95x10^-15^ | 5.66x10^-13^ | 0.18 | 5.97x10^+02^ |
| PC aa C38:4/lysoPC a C20:4 | 18.05 | 5.67 | pos. | 0.12 | 1.29x10^-11^ | 1.84x10^-09^ | 0.27 | 1.44x10^+05^ |
| PC aa C38:4/lysoPC a C16:0 | 1.26 | 0.38 | pos. | 0.12 | 6.64x10^-10^ | 9.49x10^-08^ | 0.11 | 2.80x10^+03^ |
| PC aa C38:4/lysoPC a C28:1 | 206.89 | 85.78 | pos. | 0.12 | 1.23x10^-09^ | 1.76x10^-07^ | 0.06 | 1.51x10^+03^ |
| PC aa C38:4/lysoPC a C18:0 | 4.55 | 1.46 | pos. | 0.12 | 5.00x10^-09^ | 7.15x10^-07^ | 0.07 | 3.71x10^+02^ |
| PC aa C40:4/lysoPC a C16:0 | 0.04 | 0.01 | pos. | 0.11 | 6.95x10^-09^ | 9.95x10^-07^ | 0.08 | 8.09x10^+03^ |
| PC aa C40:4/lysoPC a C28:1 | 7.30 | 3.31 | pos. | 0.11 | 2.15x10^-08^ | 3.08x10^-06^ | 0.07 | 1.15x10^+03^ |
| PC aa C40:4/lysoPC a C18:0 | 0.16 | 0.05 | pos. | 0.11 | 9.06x10^-08^ | 1.30x10^-05^ | 0.04 | 6.21x10^+02^ |
| PC aa C40:4/lysoPC a C20:4 | 0.64 | 0.23 | pos. | 0.10 | 1.38x10^-07^ | 1.97x10^-05^ | 0.15 | 4.08x10^+02^ |
| PC aa C36:4/lysoPC a C20:4 | 33.77 | 11.69 | pos. | 0.08 | 5.25x10^-06^ | 7.50x10^-04^ | 0.22 | 3.95x10^+03^ |
| PC aa C40:5/lysoPC a C16:0 | 0.12 | 0.04 | pos. | 0.09 | 1.15x10^-05^ | 1.65x10^-03^ | 0.09 | 2.13x10^+02^ |
| PC aa C40:5/lysoPC a C18:0 | 0.44 | 0.14 | pos. | 0.08 | 7.43x10^-05^ | 1.06x10^-02^ | 0.04 | 1.89x10^+02^ |
| PC ae C36:4/lysoPC a C20:4 | 3.24 | 1.10 | pos. | 0.08 | 5.17x10^-05^ | 7.39x10^-03^ | 0.15 | 4.01x10^+02^ |
| SM (OH) C14:1 | 6.38 | 1.86 | neg. | -0.12 | 1.60x10^-11^ | 2.28x10^-09^ | 0.29 |  |
| SM C16:0 | 108.15 | 21.38 | neg. | -0.10 | 1.14x10^-08^ | 1.63x10^-06^ | 0.26 |  |
| SM (OH) C22:2 | 11.74 | 3.14 | neg. | -0.10 | 2.36x10^-08^ | 3.38x10^-06^ | 0.31 |  |
| SM (OH) C16:1 | 3.42 | 0.89 | neg. | -0.09 | 1.41x10^-07^ | 2.01x10^-05^ | 0.24 |  |
| SM C26:0 | 0.18 | 0.05 | neg. | -0.07 | 2.97x10^-04^ | 4.24x10^-02^ | 0.06 |  |
| SM (OH) C16:1/SM C18:1 | 0.31 | 0.06 | neg. | -0.18 | 8.49x10^-22^ | 1.21x10^-19^ | 0.16 | 1.66x10^+14^ |
| SM (OH) C14:1/SM C18:1 | 0.58 | 0.13 | neg. | -0.19 | 1.15x10^-21^ | 1.65x10^-19^ | 0.10 | 1.39x10^+10^ |
| SM (OH) C22:2/SM C18:1 | 1.06 | 0.19 | neg. | -0.19 | 1.44x10^-21^ | 2.06x10^-19^ | 0.12 | 1.64x10^+13^ |
| SM (OH) C16:1/SM C18:0 | 0.15 | 0.02 | neg. | -0.17 | 3.75x10^-19^ | 5.36x10^-17^ | 0.14 | 3.76x10^+11^ |
| SM C16:0/SM C16:1 | 6.81 | 0.75 | neg. | -0.16 | 5.19x10^-19^ | 7.42x10^-17^ | 0.23 | 2.20x10^+10^ |
| SM C16:0/SM C18:1 | 9.91 | 1.80 | neg. | -0.16 | 1.26x10^-18^ | 1.80x10^-16^ | 0.20 | 9.07x10^+09^ |
| SM (OH) C14:1/SM C18:0 | 0.28 | 0.06 | neg. | -0.17 | 6.21x10^-18^ | 8.88x10^-16^ | 0.14 | 2.57x10^+06^ |
| SM (OH) C14:1/SM C16:1 | 0.40 | 0.08 | neg. | -0.17 | 3.13x10^-17^ | 4.47x10^-15^ | 0.08 | 5.10x10^+05^ |
| SM (OH) C22:2/SM C16:1 | 0.73 | 0.11 | neg. | -0.15 | 1.25x10^-15^ | 1.78x10^-13^ | 0.15 | 1.90x10^+07^ |
| SM C16:0/SM C18:0 | 4.73 | 0.72 | neg. | -0.15 | 2.92x10^-15^ | 4.18x10^-13^ | 0.11 | 3.90x10^+06^ |
| SM (OH) C22:2/SM C18:0 | 0.51 | 0.10 | neg. | -0.15 | 4.15x10^-15^ | 5.94x10^-13^ | 0.17 | 5.70x10^+06^ |
| SM (OH) C16:1/SM C16:1 | 0.21 | 0.04 | neg. | -0.12 | 8.20x10^-10^ | 1.17x10^-07^ | 0.09 | 1.72x10^+02^ |
| SM C18:1/SM C26:1 | 28.13 | 7.96 | pos. | 0.11 | 9.08x10^-09^ | 1.30x10^-06^ | 0.13 | 6.29x10^+04^ |
| SM (OH) C22:1/SM C18:1 | 1.26 | 0.26 | neg. | -0.11 | 7.11x10^-08^ | 1.02x10^-05^ | 0.07 | 2.74x10^+04^ |
| SM C18:1/SM C26:0 | 66.47 | 24.74 | pos. | 0.09 | 1.58x10^-07^ | 2.26x10^-05^ | 0.24 | 1.87x10^+03^ |
| SM (OH) C24:1/SM C18:1 | 0.12 | 0.03 | neg. | -0.10 | 1.62x10^-07^ | 2.32x10^-05^ | 0.08 | 5.50x10^+03^ |
| SM C18:1/SM C24:1 | 0.21 | 0.04 | pos. | 0.09 | 6.49x10^-07^ | 9.28x10^-05^ | 0.18 | 3.81x10^+04^ |
| SM C18:0/SM C26:1 | 58.18 | 14.21 | pos. | 0.10 | 7.30x10^-07^ | 1.04x10^-04^ | 0.10 | 7.82x10^+02^ |
| SM (OH) C22:1/SM C24:0 | 0.63 | 0.09 | neg. | -0.09 | 2.79x10^-06^ | 3.99x10^-04^ | 0.22 | 6.97x10^+02^ |
| SM (OH) C22:1/SM C18:0 | 0.60 | 0.11 | neg. | -0.09 | 3.82x10^-06^ | 5.46x10^-04^ | 0.07 | 5.09x10^+02^ |
| SM (OH) C24:1/SM C18:0 | 0.06 | 0.01 | neg. | -0.09 | 4.93x10^-06^ | 7.05x10^-04^ | 0.07 | 1.81x10^+02^ |
| SM C16:1/SM C18:1 | 1.45 | 0.19 | neg. | -0.09 | 1.19x10^-05^ | 1.71x10^-03^ | 0.05 | 2.59x10^+03^ |
| H1 | 5005.34 | 670.72 | pos. | 0.10 | 3.34x10^-07^ | 4.77x10^-05^ | 0.13 |  |

^a^ Fat Free Mass Index; ^b^ direction of the association (positive or negative); ^c^ for multiple testing adjusted p-value; ^d^ adjusted R^2^ of the linear model; ^e^ p-gain, fold decrease in the P value of association for the pair of metabolites, compared to the lowest of two p values for the single metabolites; AAs amino acids; Σ aromatic amino acids is the sum of tyrosine, phenylalanine, and tryptophan; Σ BCAAs is the sum of valine and the combination of isoleucine and leucine; Σ glucogenic amino acids is the sum of glycine and serine.
