# Supplementary material for: Body Fat Free Mass Is Associated with the Serum Metabolite Profile in a Population-Based Study
Source: PLoS One. 2012 Jun 27;7(6):e40009. doi: 10.1371/journal.pone.0040009 (PMC3384624; doi:10.1371/journal.pone.0040009)
Supplement: Table S4 — List of selected SNP, which were significantly associated in genome wide association studies with anthropometric characteristic as outcomes and tested in the present study for associations with metabolomics data. (DOC) [file pone.0040009.s005.doc]

**Table S4:** List of selected SNP, which were significantly associated in genome wide association studies with anthropometric characteristic as outcomes and tested in the present study for associations with metabolomics data.

| rs16892496 | rs3888190 | rs10840077 | rs12970134 |
| --- | --- | --- | --- |
| rs11785269 | rs9816226 | rs206936 | rs11664883 |
| rs7832552 | rs3817334 | rs464553 | rs1350341 |
| rs7828207 | rs7928842 | rs9939609 | rs975918 |
| rs9491696 | rs543874 | rs11075990 | rs633265 |
| rs7766106 | rs506589 | rs6548238 | rs487720 |
| rs6905288 | rs987237 | rs7601028 | rs1077393 |
| rs984222 | rs734597 | rs17782313 | rs1052486 |
| rs984225 | rs7138803 | rs10871777 | rs2260000 |
| rs1055144 | rs7132908 | rs10938397 | rs2736172 |
| rs2893221 | rs10150332 | rs12641981 | rs2844479 |
| rs10195252 | rs10145154 | rs7498665 | rs2736176 |
| rs10184004 | rs713586 | rs8055982 | rs3101336 |
| rs4846567 | rs713587 | rs9931989 | rs2815752 |
| rs2820446 | rs12444979 | rs7189927 | rs3751812 |
| rs1011731 | rs12446554 | rs10838738 | rs11075989 |
| rs991790 | rs2241423 | rs4752857 | rs6499640 |
| rs718314 | rs4776375 | rs11084753 | rs7203521 |
| rs10842703 | rs2287019 | rs368794 | rs7190492 |
| rs1294421 | rs1800437 | rs2815752 | rs12446228 |
| rs1294420 | rs1514175 | rs1460943 | rs8044769 |
| rs1443512 | rs1514176 | rs2568958 | rs11075987 |
| rs1822438 | rs13107325 | rs3101336 | rs8050136 |
| rs6795735 | rs1813006 | rs10769908 | rs8051591 |
| rs7428936 | rs2112347 | rs1900273 | rs10913469 |
| rs4823006 | rs6874626 | rs2145270 | rs545608 |
| rs12321 | rs10968576 | rs6054427 | rs7647305 |
| rs6784615 | rs16912921 | rs12324805 | rs7635103 |
| rs11721286 | rs3810291 | rs7178753 | rs4788102 |
| rs6861681 | rs4804023 | rs29941 | rs7187333 |
| rs747472 | rs887912 | rs29942 | rs8049439 |
| rs2076529 | rs763712 | rs10501087 | rs7187776 |
| rs2076530 | rs13078807 | rs925947 | rs2867125 |
| rs7081678 | rs9826482 | rs10835211 | rs939582 |
| rs7085067 | rs11847697 | rs11030107 | rs4854344 |
| rs1558902 | rs2890652 | rs4074134 | rs13393304 |
| rs1421085 | rs1555543 | rs4923457 | rs7561317 |
| rs571312 | rs11165643 | rs4923461 | rs11127491 |
| rs663129 | rs4771122 | rs4923460 | rs987237 |
| rs10767664 | rs12016871 | rs6265 | rs2206277 |
| rs988748 | rs4836133 | rs2049045 | rs2605100 |
| rs7359397 | rs6864049 | rs925946 |  |
| rs4929949 | rs10767658 | rs2605097 |  |
